# Structural Constraint of Osteopontin Facilitates Efficient Binding to CD44

**DOI:** 10.3390/biom11060813

**Published:** 2021-05-30

**Authors:** Gulimirerouzi Fnu, Palak Agrawal, Gopal C. Kundu, Georg F. Weber

**Affiliations:** 1 College of Pharmacy, University of Cincinnati Academic Health Center, Cincinnati, OH 45229, USA; fnugi@mail.uc.edu; 2 National Centre for Cell Science, Pune 411007, Maharashtra, India; 92.palak@gmail.com (P.A.); gopalkundu@hotmail.com (G.C.K.)

**Keywords:** receptor, ligand, heparin, hyaluronate, protein complex, CD44, osteopontin, integrin

## Abstract

Since the original description in 1996, the interaction between the cytokine osteopontin (OPN) and the homing receptor CD44 has been extensively studied in cancer, inflammation, bone remodeling, and various other conditions. Alternative splicing and extensive posttranslational modifications by both binding partners, as well as the possibility for lateral recruitment of additional membrane receptors or soluble co-ligands into a complex have left the exact molecular requirements for high-affinity OPN-CD44 binding unresolved. We now report that there is a moderate engagement between the unmodified molecules, which results in curved double-reciprocal plots for OPN titration, suggesting the existence of two binding sites or two binding conformations. Structural constraint of OPN, by immobilization or by addition of heparin, is required for its strong ligation of CD44. Prior literature provides evidence that heparin binding to OPN prompts the unfolding of a core element in the protein. This conformational adjustment may be essential for efficient CD44 interaction. The integrin α_9_β_1_ seems to compete with the OPN-CD44 engagement, while the integrin α_V_β_3_ reflects additive binding, suggesting that the CD44 contact sites on OPN are downstream of the RGD motif but overlap with the SVVYGLR domain. Hyaluronate has no effect, placing the relevant domain on CD44 downstream of the N-terminus.

## 1. Introduction

Osteopontin (OPN) plays essential roles in tissue remodeling, cellular immune responses, and the calcium homeostasis of milk and urine. In pathophysiology, it contributes to the dissemination of multiple cancers. OPN accomplishes many of its tasks through interactions with various receptors. The central portion of the molecule—from the poly-aspartate sequence through the thrombin cleavage site—harbors several integrin binding domains [1]. The C-terminal portion of OPN has been tentatively mapped to engage the homing receptor CD44 [2,3].

CD44 was the first cell surface receptor to be functionally associated with cancer metastasis [4]. The identification of OPN, which independently had been linked to cancer progression [5], as a CD44 ligand offered implications for molecular oncology [6] and for cellular immune responses [7]. Although the initial report describing OPN effects to be exerted on cells via CD44 [8] has been amply corroborated (Supplementary Table S1), there have been conflicting observations regarding the CD44 splice variants that are permissive for OPN binding and regarding the possible contributions to the interaction by recruited integrins. Further, it is yet unknown whether the CD44 ligand hyaluronate can have synergistic or antagonistic functions, and whether a heparin bridge or a heparin-dependent conformational change in OPN can support a high affinity engagement. Elucidation of these binding characteristics is a necessity for realizing the potential to target the OPN-CD44 interaction in anti-cancer treatment.

The structural and functional versatility of the cell surface receptor CD44 is high. Besides giving rise to multiple splice variants and being extensively glycosylated, it can laterally recruit the β-chains of several integrins [3], and it may function as a co-receptor for growth factor pathways including cMET, EGFR, HER-2, and VEGFR [9]. Due to the ligand of interest, OPN, being subject to alternative splicing and to an abundant diversity of posttranslational modifications, the number of possible permutations that need to be tested for comprehensively explaining the nature of OPN and CD44 interactions, individually or in a complex with other molecules, is large. In this study, we venture into elucidating the binding characteristics between these important proteins.

## 2. Materials and Methods

### 2.1. Reagents

Recombinant human OPN (Ile17 through Asn287 of accession # P10451), labeled with N-terminal His-and GST-tags, was obtained from Wuhan USCN Business Co., (Wuhan, China) and used in the surface plasmon resonance experiments. Bacterial recombinant GST-OPNa (after deletion of the signal sequence [10]) was used for the ELISA and pull-down experiments; the results were also corroborated with a truncated GST-OPN (amino acids 59–300). The recombinant extracellular domain of CD44 (aa21–606), labeled with His-and SUMO-tags, came from Lifespan Biosciences. This CD44 variant (isoform 2, Epican) lacks an in-frame coding exon compared to variant 1 but contains the variant exons 3–10. The extracellular portions of the integrins α_9_β_1_ (Tyr30 through Tyr977 of α_9_, accession # Q13797, with a poly-histidine tail; Gln21 through Asp728 of β_1_, accession # P05556), α_4_β_1_ (Tyr34 through Gln970 of α_4_, accession # P13612, with a poly-histidine tail; Gln21 through Asp728 of β_1_, accession # P05556) and α_V_β_3_ (Phe31 through Val992 of α_V_, accession # NP002201; Gly27 through Asp718 of β_3_, accession # AAA52589) were purchased from R&D Systems. Hyaluronic acid sodium salt from *Streptococcus equi*, molecular weight 1200, as well as heparin came from Sigma-Aldrich (St. Louis, MO, USA).

### 2.2. Surface Plasmon Resonance

Binding was analyzed on a SPR-based biosensor BIACORE 2000 instrument (Biacore AB, Uppsala, Sweden), utilizing a CM5 Chip (GE Healthcare Life Sciences, Marlborough, MA, USA). About 8000 RUs (resonance units, 1 RU corresponds to approximately 1 pg/mm^2^) of each protein (GST-OPN, CD44v or integrin α_9_β_1_) was immobilized on test flow cells (Fc-2, Fc-3 or Fc-4) respectively through an amine coupling on a CM5 chip and Fc-1 immobilized with BSA served as control flow cell. Buffer exchange was performed for OPN and CD44v to eliminate Tris from the protein preparations. Binding was assessed in 10 mM HEPES buffer, 150 mM NaCl, pH 7.4 containing 0.05% Tween 20 at 25 °C. For every run, 0.4 mM MnCl_2_ was added. Proteins were then flowed at various concentrations over the CM5 chip at 50 μL/min for 120 s and was followed by additional dissociation time for 180 s. The chip was regenerated with 0.2 M sodium carbonate, pH 9.5. The data was evaluated using BIAevaluation software version 4.1 where the data from test flow cell was subtracted from the control flow cell.

### 2.3. Enzyme-Linked Immunosorbent Assay

Recombinant CD44v in 100 μL PBS was immobilized on a high-binding 96-well plate overnight at 4 °C. A standard curve was generated by immobilizing increasing amounts of GST-OPNa. The wells were then blocked with 5% BSA in PBS for 2 h at room temperature (blocking with 5% casein gave similar results, ELISA Ultrablock (BioRad, Hercules, CA, USA) suppressed the signal). Following 3 washes with binding buffer (10 mM HEPES buffer, pH 7.4, plus 150 mM NaCl, 2 mM MnCl_2_ and 0.05% Tween 20), various concentrations of GST-OPNa in the presence or absence of the indicated amounts of heparin were incubated for binding over 2 h at room temperature. After 3 washes, polyclonal HRP-anti-GST epitope tag (Novus Biologicals, Littleton, CO, USA) at 1:10,000 dilution was incubated for 1 h at room temperature. Consecutive to 3 final washes, 100 μL/well of the detection reagent TMB (3,3′,5,5′-tetramethylbenzidine; Surmodics BioFx, Eden Prairie, MN, USA) was provided, color development was monitored, and the reaction was terminated with stopping reagent (Surmodics TMB Stop Solution) at the appropriate time. The absorbance of each well was read at a wavelength of 450 nm.

### 2.4. Pull-Down

We performed binding assays by incubating the indicated amounts of GST-OPN [10] with the recombinant extracellular domain of CD44v (Lifespan Biosciences, Seattle, WA, USA) in 10 mM HEPES buffer, pH 7.4, plus 150 mM NaCl and 0.05% Tween 20. Heparin or divalent cations were added as indicated. A tube prepared in parallel contained 10% of the input. After 1 h at 4 °C, either GSH-Sepharose was added to pull down GST-OPNa, or nanoCLAMP resin (Nectagen, Kansas City, KS, USA) was added to pull down CD44v via its SUMO-tag. In select experiments, the order was reversed and the pull-down of the target molecule (1 h at 4 °C) preceded the addition of the binding partners. Following another hour of incubation at 4 °C, the resins were washed four times, and the bound fractions were released with reducing SDS-PAGE sample buffer and heating. The eluted proteins were resolved on a 10% polyacrylamide gel and detected via Western blotting with anti-OPN antibody O-17 (IBL America, Minneapolis, MN, USA) or anti-His-tag antibody (ThermoFisher, Waltham, MA, USA) and ECL visualization.

## 3. Results

### 3.1. OPN and CD44 Interact More Strongly under Structural Constraint of OPN

**Figure 1:**
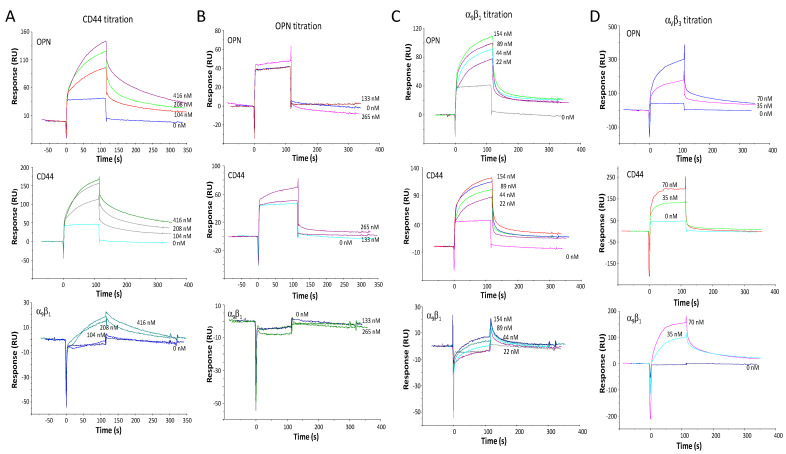
Surface plasmon resonance for the assessment of one-on-one binding between OPN, CD44 and integrin receptors. In a Bioacore 2000 instrument, the four channels (Fc-1, Fc-2, Fc-3, or Fc-4) of a CM5 chip were coated with bovine serum albumin (negative control), GST-OPN, CD44v, or integrin α_9_β_1_ respectively: (**A**) Binding of the CD44v extracellular domain (N-terminally tagged with 6XHis and SUMO) in the flow to immobilized OPN, to immobilized CD44v extracellular domain, or to immobilized α_9_β_1_ extracellular domain: (**B**) Binding of recombinant GST-OPN in the flow to immobilized OPN, to immobilized CD44v extracellular domain, or to immobilized α_9_β_1_ extracellular domain; (**C**) Binding of the integrin α_9_β_1_ in the flow to immobilized OPN, to immobilized CD44v extracellular domain, or to immobilized α_9_β_1_ extracellular domain; (**D**) Binding of the Integrin α_V_β_3_ in the flow to immobilized OPN, to immobilized CD44v extracellular domain, or to immobilized α_9_β_1_ extracellular domain.

Due to surface plasmon resonance being a method for the rapid assessment of molecular interactions, we first assessed binding with this tool. The extracellular portion of CD44v bound to immobilized OPN. Its binding to integrin α_9_β_1_ was low. CD44v in the flow also bound to immobilized CD44v (Figure 1A). This is consistent with a literature report that variant, but not standard CD44 can aggregate [11], which is consistent with these surface plasmon resonance results (see also Supplementary Figure S1). As was described in the original report [8], the interaction between OPN and CD44 is independent of glycosylation (both proteins used here are bacterial recombinant products). GST-OPN in the flow did not bind to immobilized GST-OPN or to immobilized α_9_β_1_. Unexpectedly, it showed only low-level binding to immobilized CD44v (Figure 1B). This suggested that OPN requires some structural constraint (as it is exerted by immobilization on the SPR chip) for engaging CD44. Separately, the integrins α_9_β_1_ or α_V_β_3_ in the flow were assessed for binding to immobilized OPN, immobilized CD44v extracellular domain, or immobilized α_9_β_1_ extracellular domain (Figure 1C,D, see description below).

**Figure 2:**
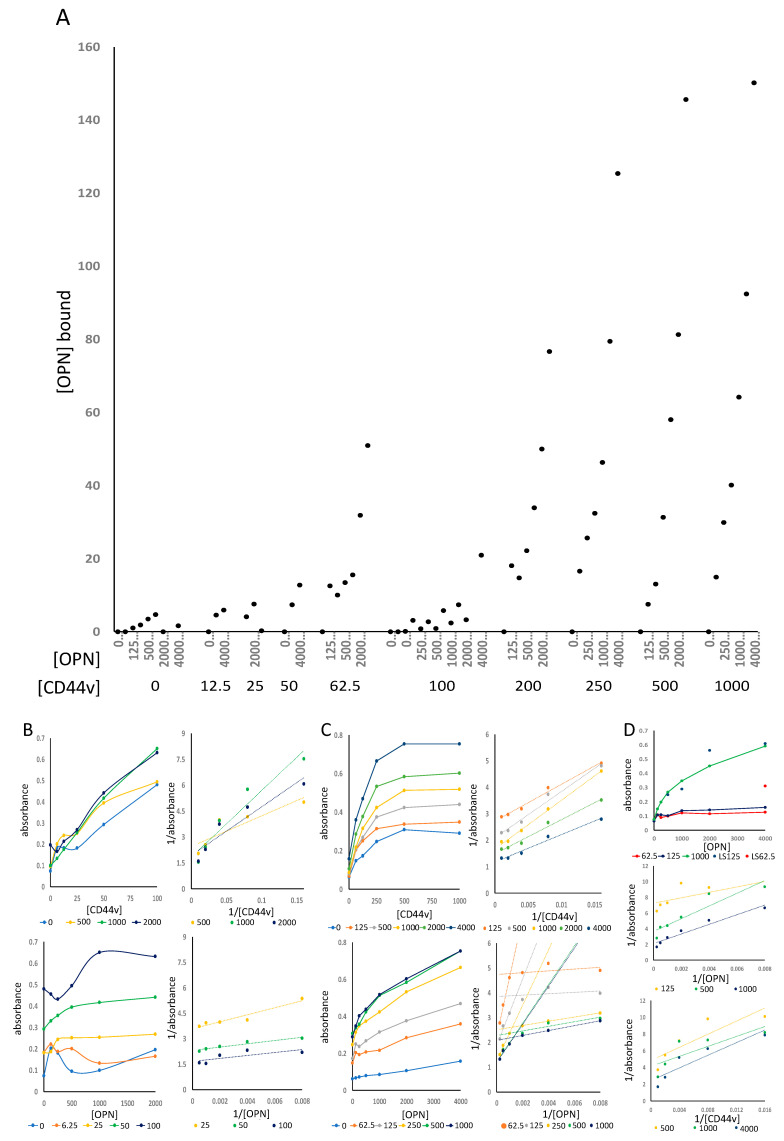
OPN binding to CD44 in ELISA: (**A**) Summary of several ELISA experiments titrating OPN (top row of x-axis label) and CD44v (bottom row of x-axis label). The y-axis shows nanograms of OPN bound per well (calculated from a standard curve of plated OPN), the x-axis displays the amount (ng) of CD44v and OPN per well; (**B**,**C**) Receptor-ligand ELISA with low (**B**) or high (**C**) concentration ranges of the interaction partners. The middle panel displays the absorbance versus [CD44v] for various concentrations of OPN in solution, followed by the corresponding double-reciprocal graph. The bottom panel displays the absorbance versus [OPN] for various concentrations of immobilized CD44v, followed by the corresponding double-reciprocal graph; (**B**) Titration of immobilized CD44v (0–100 ng/well) and soluble GST-OPNa for binding (0–2000 ng/well); (**C**) Titration of immobilized CD44v (0–1000 ng/well) and soluble GST-OPNa for binding (0–4000 ng/well); (**D**) OPNa binding to truncated CD44, dose-response titrations in ELISA; (**top**) titration of OPN at various amounts of truncated CD44v per well (lines and markers) and various amounts of full-length CD44v per well (markers), (**middle panel**) double reciprocal plot for OPN titration, (**bottom panel**) double reciprocal plot for truncated CD44v (labeled LS) titration.

We expanded the measurements to a receptor-ligand ELISA format, where CD44 was immobilized on a high-binding 96-well plate and GST-OPN was added at various concentrations, followed by detection with a HRP-anti-GST epitope tag antibody, which does not interfere with the interactions among the binding partners. The color development of the detection reagent TMB was assessed. The titration of both, OPN and CD44v, resulted in increasing absorbance (Figure 2A). At low concentrations (CD44v 0–100 ng/well, GST-OPNa 0–2000 ng/well), CD44v titrated dose-dependently whereas OPN displayed absorbance increases only at the more elevated doses in the chosen range (Figure 2B). At high concentrations (CD44v 0–1000 ng/well, GST-OPNa 0–4000 ng/well), CD44v titrated saturably (Figure 2C) and could be converted to linear double-reciprocal plots. OPN bound dose-dependently; remarkably the double-reciprocal plots and the Scatchard plots (not shown) consistently displayed curved lines, suggesting the possibility of either two binding sites with distinct affinities or two binding conformations. Notably, OPN binding to a truncated version of CD44v (MyBiosource, San Diego, CA, USA, alignment in Supplementary Figure S1) appeared to have about 8-fold lower affinity (overlapping dose-response curves for osteopontin binding to 125 ng/well LSBio CD44v and 1000 ng/well MyBiosource CD44v) suggesting that the binding domain on CD44 entails and extends beyond the truncated version (Figure 2D).

### 3.2. Heparin Enables a Strong OPN-CD44 Interaction

**Figure 3:**
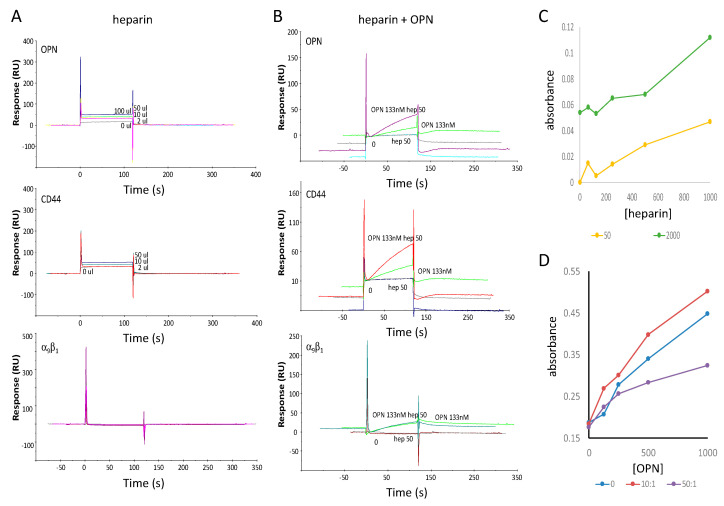
Heparin facilitates OPN binding to CD44 in surface plasmon resonance experiments. In a BIACORE 2000 instrument, the four channels of a CM5 chip were coated with bovine serum albumin (negative control), GST-OPN, CD44v, or Integrin α_9_β_1_: (**A**) Low levels of heparin binding alone to immobilized OPN (**top**), CD44v (**middle**), or α_9_β_1_ (**bottom**); (**B**) Binding of heparin, OPN or both in the flow to immobilized OPN (**top**), CD44v (**middle**), or α_9_β_1_ (**bottom**); (**C**) Heparin effect on OPN-CD44v binding in ELISA; (**D**) OPN binding to CD44v in ELISA, following preincubation with heparin for 2 h at the indicated ratios.

Binding of the intrinsically disordered protein OPN to the polysaccharide heparin is accompanied by thermodynamically compensating structural adaptations, which is reflected in an expansion of the OPN core segment upon interaction. This unfolding is governed primarily via electrostatic forces between heparin and charged patches along the protein backbone, and it balances for the entropic losses encountered through ligand engagement [12]. Upstream of the primary heparin binding site (amino acids 150-165), a preformed compensatory element is located that masks the domain in the absence of heparin, but leaves it exposed to solvent in the bound state [13]. The OPN interaction with heparin leads to a reduction of correlated long-range motions and thus reflects a loosening of structural compaction. Heparin binding to the primary site causes a rigidification of the region, exchange broadening, or a combination of both. Upon complex formation, the conformational coupling in the apo-state is lost for most of the segments [14]. The addition of heparin to OPN in the surface plasmon resonance flow was sufficient for an increase in the affinity for binding between flowing OPN and immobilized CD44v (Figure 3A,B), suggesting that heparin provided the required structural constraint of OPN. Heparin also seemed to facilitate some level of OPN-OPN interaction. In ELISA, direct addition or preincubation of OPN and heparin before addition to the CD44v-coated plates caused enhanced binding at a ratio of 10:1 on a mass basis. Consistent with the pull-down assay results below, a high amount of heparin (ratio 50:1) resulted in inhibition (Figure 3C,D).

**Figure 4:**
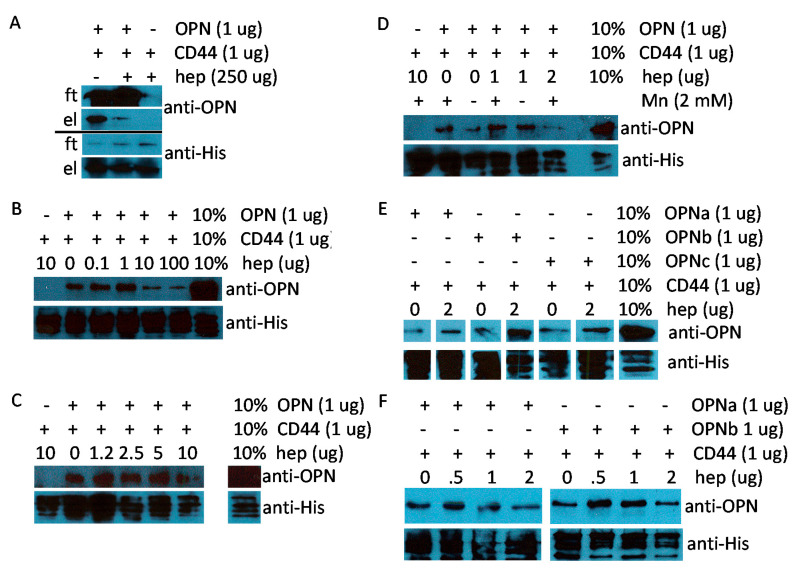

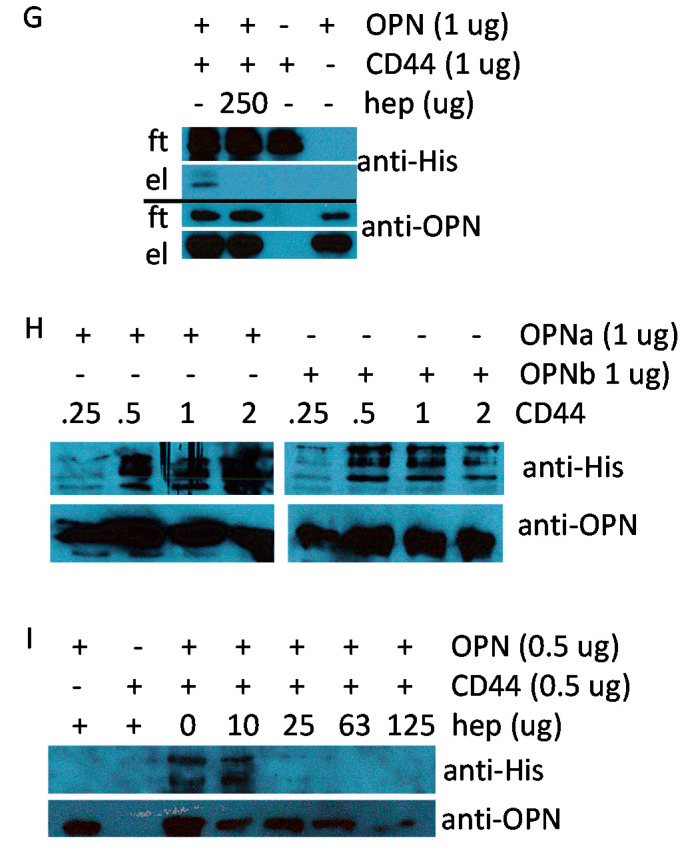
Pull-down of OPN or CD44 after binding. Binding was assessed between GST-OPN and the extracellular domain of CD44v. Following resolution of the proteins on 10% SDS-PAGE and Western blotting, the signal for OPN was detected by probing with antibody O-17, and the signal for CD44v was detected by probing with anti-His-tag antibody. Each subfigure shows the results from one experiment and one gel: (**A**–**F**) Pull-down of the extracellular CD44v domain with nanoCLAMP resin; (**A**) Flow-through and eluate of OPN and CD44v in the presence or absence of 250 μM heparin. ft = flow-through, el = elution. One representative experiment of two (the densitometry results are shown in Supplementary Figure S4); (**B**) OPN and CD44v binding under titration of heparin (0–100 μM). One representative experiment of two; (**C**) OPN and CD44v binding under titration of heparin (0–10 μM); (**D**) Heparin at 0, 1, or 2 μg in the presence or absence of 2 mM manganese chloride (Mn); (**E**,**F**) Binding of OPN splice variants (GST-OPNa, GST-OPNb, GST-OPNc) to CD44v in the presence or absence of heparin (0–2 μM); (**G**–**I**) Pull-down of OPN with GSH-resin; (**G**) Flow-through and eluate of OPN and CD44v in the presence or absence of 250 μM heparin. ft = flow-through, el = elution; (**H**) CD44v titration (0.25–2 μg) to GST-OPNa or GST-OPNb (1 μg) in the absence of heparin. One representative experiment of two; (**I**) OPN and CD44v binding under titration of heparin (0–125 μM).

We sought to corroborate the surface plasmon resonance and ELISA results with pull-down assays. When using nanoCLAMP to target CD44, low-level binding was seen between CD44v and OPN. When the proteins were used at 1 μg each, the presence of 10–250 μM heparin interfered with the interaction. However, 1.25–5 μg heparin enhanced the engagement (Figure 4A–C). We applied a standard concentration of 2 mM manganese as a provider of divalent cations in our binding buffers. Its omission led to a small reduction in binding, suggesting that manganese contributes to, but is not required for the interaction (Figure 4D). Of note, in the ELISA format, the replacement of manganese with 2 mM magnesium plus 2 mM calcium prevented OPN binding to CD44v but not to integrin α_V_β_3_ (Supplementary Figure S3). All OPN splice variants, OPNa, OPNb, and OPNc, interacted with CD44v, and their binding was supported by 1–2 μg heparin (Figure 4E,F). It was feasible to reverse the pull-down set-up by capturing OPN with GSH-sepharose (Figure 4G). Extending what had been seen in the surface plasmon resonance, CD44v bound dose-dependently to immobilized OPNa or OPNb (Figure 4H). At elevated concentrations, heparin inhibited this interaction (25–250 μg in the 100 μL assay volume) (Figure 4G–I). Hence, OPN modification by immobilization or heparin binding is required for strong CD44 engagement. Heparin at low concentration is supportive of the OPN-CD44 interaction, whereas it becomes inhibitory at high concentration.

### 3.3. The OPN-CD44 Binding Has No Requirement for Integrin Receptors or Hyaluronate

**Figure 5:**
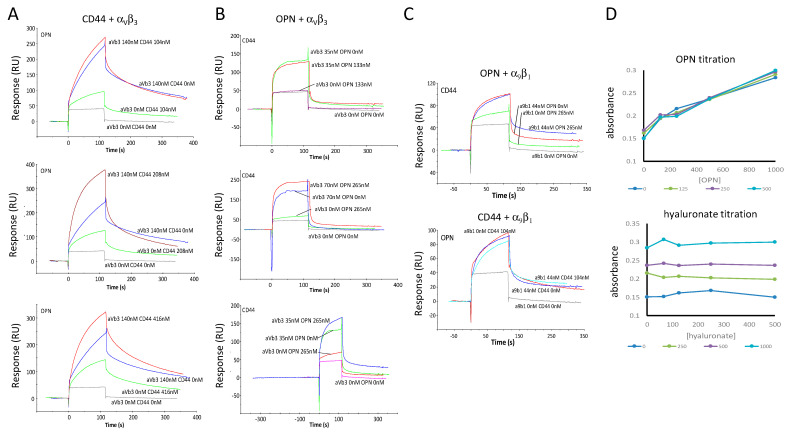
Multiple binding interactions among OPN, CD44, integrins and hyaluronate. In a BIACORE 2000 instrument, the four channels of a CM5 chip were coated with bovine serum albumin (negative control), GST-OPN, CD44v, or integrin α_9_β_1_: (**A**) Co-titration of CD44v and α_V_β_3_ in the flow to bound OPN; (**B**) Co-titration of OPN and α_V_β_3_ to immobilized CD44v; (**C**) Co-titration of OPN and α_9_β_1_ to bound CD44v (**top panel**) and co-titration of CD44v and α_9_β_1_ to bound OPN (**bottom panel**); (**D**) Co-titration of OPN and hyaluronate in CD44v-binding ELISA; (**top panel**) the y-axis displays absorbance units, the x-axis shows increasing amounts of OPN, each line refers to a specific nanogram amount of hyaluronate as indicated in the legend; (**bottom panel**) the y-axis displays absorbance units, the x-axis shows increasing amounts of hyaluronate, each line refers to a specific nanogram amount of OPN as indicated in the legend.

The multitude of ligands for various forms of CD44, various receptors for OPN, and the possibility of receptor interactions within the cell membrane raised the possibility that the bilateral interaction between OPN and CD44 could be influenced—positively or negatively—by other players. In this regard, integrins are candidate membrane structures, while heparin (investigated above) and hyaluronate are candidate soluble molecules. Being thoroughly studied OPN receptors, the flowing integrins α_9_β_1_ and α_V_β_3_ bound dose-dependently to immobilized OPN in surface plasmon resonance. They also bound to CD44v, consistent with observations that CD44 can laterally recruit integrins to form receptor complexes in the cell membrane. Integrin α_9_β_1_ in the flow bound more avidly to OPN and to CD44v than amine-coupled, immobilized integrin α_9_β_1_ bound to the same binding partners in the mobile phase (see Figure 1), possibly reflecting an adverse impact on its structural integrity by the binding to the surface plasmon resonance membrane. It is also conceivable that the structural constraint of OPN immobilization can mimic the physiologic requirement of OPN phosphorylation for integrin α_9_β_1_ binding, so that flowing bacterial recombinant OPN shows no interaction with this integrin, whereas immobilized OPN with integrin α_9_β_1_ in the flow does. Flowing integrin α_V_β_3_ did bind to immobilized α_9_β_1_, which may have a physiologic correlate in integrin clustering (see Figure 1C,D). Flowing α_V_β_3_ together with either OPN (in the channel with immobilized CD44v) or CD44v (in the channel with immobilized OPN) indicated additive binding (Figure 5A,B), implying that the binding sites for this integrin on OPN as well as on CD44 are distinct from the interaction domains between CD44 and OPN. (The lack of competition by integrin α_V_β_3_ (0–250 ng/well) was confirmed in ELISA with 200 ng plated CD44v and OPN titration (0–1000 ng/well) (data not shown).) When co-flowing integrin α_9_β_1_, by contrast, it seemed to compete with CD44v-OPN binding (Figure 5C). The implication of these findings is that the CD44 binding domain on OPN is C-terminal from the RGD motif, but encroaches on the α_9_β_1_ engagement site SVVYGLR, which localizes immediately downstream.

Hyaluronate is known to bind to the far N-terminal domain of CD44 [15,16]. In the ELISA format (manganese-containing buffer), low molecular weight hyaluronate (0–500 ng) had no effect on the interaction between plated CD44v (200 ng) and OPN in solution (0–1000 ng) (Figure 5D). This is in keeping with the model that these two ligands bind to distinct receptor domains (in the original report, the antibody IM7 that recognizes the hyaluronate-binding N-terminus of CD44 did not affect the OPN-CD44 engagement) and exert differential effects on CD44v-bearing cells [8].

### 3.4. OPN Binding to CD44 Has Distinct Kinetics from OPN Binding to Integrin α_V_β_3_

**Figure 6:**
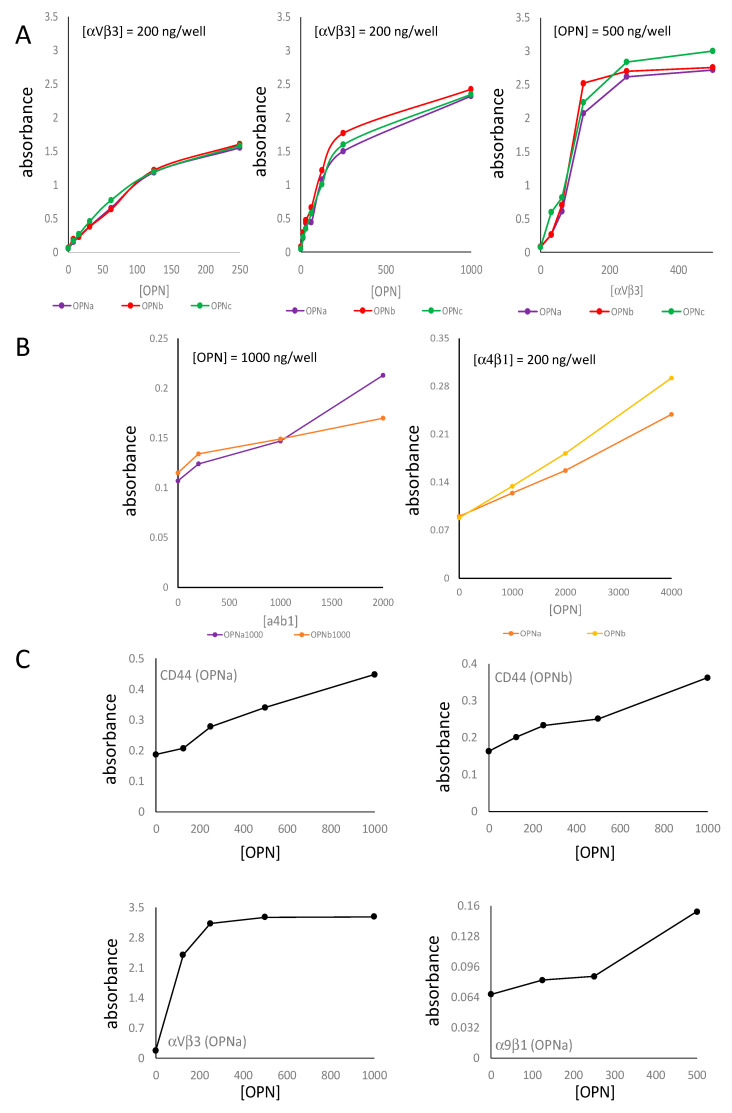
Comparison of CD44 binding to integrin binding by OPN. In receptor-ligand ELISA, 200 ng of receptors were plated and OPN was titrated at 200–1000 or 200–4000 ng (plus a 0 ng OPN control): (**A**) Integrin binding by the OPN splice variants; (**left**) low-dose titration of OPN at 200 ng/well integrin α_V_β_3_ (**middle**) high-dose titration of OPN at 200 ng/well integrin α_V_β_3_ (**right**) titration of integrin α_V_β_3_ at 500 ng/well OPN; (**B**) Integrin α_4_β_1_ binding by the splice variants OPNa or OPNb at 2 mM manganese; (**left**) with OPN at 1000 ng/well, titration of integrin α_4_β_1_ (**right**) titration of OPN at 200 ng/well integrin α_4_β_1_; (**C**) (**top left**) plated CD44v and bound OPNa (one representative experiment of >10); (**top right**) plated CD44v and bound OPNb; (**bottom left**) plated integrin α_V_β_3_ and bound OPNa (one representative experiment of 6); (**bottom right**) plated integrin α_9_β_1_ and bound OPNa (note the different scales of the y-axes).

Finally, we compared OPN-CD44v binding to OPN-integrin binding in ELISA. All three splice forms of OPN bind comparably to integrin α_V_β_3_ (Figure 6A). Integrin α_4_β_1_ has been reported to bind to OPN independently of posttranslational modifications [17]. This integrin can be activated by manganese. OPNa and OPNb displayed low-level binding to integrin α_4_β_1_ at 2 mM MnCl_2_ in the buffer (Figure 6B). Titration of manganese over 0–8 mM showed little effect on the interaction (not shown). With immobilized receptor at 200 ng per well, OPN up to 1000 ng saturated the engagement of integrin α_V_β_3_, whereas only minimal binding occurred to integrin α_9_β_1_. The interaction with CD44v by either OPNa or OPNb was intermediate, but it had not yet saturated in this range of reactants (Figure 6C). From the double-reciprocal plots, we calculated half-maximal and maximal OPN bound to its receptors, using the monophasic sections where necessary (Table 1).

## 4. Discussion

We find that all three splice variants of GST-OPN moderately engage the extracellular domain of CD44v. They do so with curved double-reciprocal plots. OPN immobilization or intermediate concentrations of heparin support strong interaction (high-concentration heparin inhibits). Manganese has a mildly supportive, albeit not required function, whereas magnesium plus calcium, at 2 mM each, interfere with binding. As both proteins used here are bacterial recombinant, phosphorylation or glycosylation do not seem to play substantive roles. This is relevant, as a substantial fraction of the native molecular weights for OPN as well as CD44 are accounted for by glycosylation. Further, OPN has a large number of phosphorylation sites.

These experiments narrow down the binding domains on both molecules. Hyaluronate (which binds to the far N-terminus of CD44) does not affect the OPN-CD44 interaction. We have not tested other forms of CD44, such as CD44s, which lacks variant exons, but we have found partial binding by a truncated CD44v. Therefore, the required CD44 domain is downstream of the N-terminus and likely covers several variant exons (the form predominantly used in this study is transcriptional variant 2). While the lateral recruitment of integrin α_V_β_3_, which can occur in the cell membrane, appears additive with no detectable impact on OPN-CD44 affinity, its binding in a complex could alter the signal transduction resulting from OPN-CD44 engagement. By contrast, integrin α_9_β_1_ competes with the OPN-CD44 interaction. By inference, the required OPN structure likely is located downstream of the RGD motif and starts upstream of the central heparin binding site (consistently, the shorter GST-OPN, starting at amino acid 59, displays no reduction in binding).

The high flexibility resulting from the largely unstructured conformation of OPN may enable the relatively small glycoprotein to rapidly associate with a number of diverse binding partners [18]. Although OPN does not fold into a single defined structure, its conformational flexibility significantly deviates from random coil-like behavior. Its backbone does not only exhibit characteristics of an extended and flexible polypeptide, but also characteristics of a globular protein. Both conformations, extended and cooperatively folded ones, are assumed simultaneously by OPN in its apo-state [19]. While a defined overall structure has not been discernible, OPN comprises distinct local secondary structure elements with reduced conformational flexibility. It substantially populates a compact region displaying tertiary contacts. Such regional structural preformation leads to a reduction of the accessible structural space. The compacted domains of OPN encompass the binding sites for integrin a_V_β_3_ and heparin [20,21,22,23]. OPN exhibits a long-range intramolecular communication between the N- and C-terminal regions. Functional connections with its receptors, including integrins and CD44, may be modulated by such intramolecular interactions [24].

The OPN structure can be constrained by immobilization, as is the case with amine coupling on a metal film (the surface plasmon resonance experiments of this study) or inside micro-emulsion droplets [25]. Yet, it is important to correlate these artificial in vitro conditions with physiologic environments. Our findings suggest that the efficient interaction with the extracellular domain of CD44 is contingent with accessibility to the compacted core regions of OPN, which physiologically may be achieved via heparin binding.

The structural flexibility of OPN possibly affects two of its important biological functions: the property as an extracellular matrix component and the role as a cytokine.

- OPN can work by bridging two proteins of fixed configurations into a biologically active complex [26]. The low conformational constraints may also allow multiple binding geometries in the adsorption of the acidic protein to calcium-rich crystal faces of biominerals, which is largely governed by the sequential formation of ionic bonds with the crystal surface [27,28]. Binding to crystalline hydroxyapatite may induce a small increase of β-sheet in OPN, which, in solution, exhibits a predominantly random coil structure, mostly unaffected by the addition of dissolved calcium [29].
- OPN is secreted and exerts differential effects on designated target cells when presented either in solution or after immobilization [30]. Cross-linking to the matrix may occur via transglutamination. Three candidate transglutamination sites are located on the far N-terminus (after Thrombin cleavage, only the integrin-binding N-terminal fragment is subject to transglutamination). Although we find that coupling of OPN to the metal film of the surface plasmon resonance chip facilitates the interaction with CD44, we suspect heparin binding but not immobilization to be the primary physiologic mechanism. This notion is derived from results with CD44-expressing and integrin-expressing cells. OPN can induce chemotaxis via CD44 and haptotaxis via integrin receptors [2], the latter of which is contingent with immobilization of OPN or its N-terminal fragment.

Biologically relevant interactions between OPN and heparin have been described in the literature. Thrombin cleavage is a critical determinant for OPN function. It is inhibitable by unfractionated heparin [31]. Heparin acts as an inhibitor of factor-independent protein kinase (FIPK) activity and can block the phosphorylation of OPN by microsomal kinases [32,33]. OPN forms rapid and tight complexes with complement Factor H [34], which also binds tightly to heparin [35]. OPN specifically binds to IGFBP-5 with high affinity. Free heparin and heparan sulfate compete with the interaction, and it may be important for concentrating intact IGFBP-5 in the extracellular matrix [36]. Further, we have previously suggested that heparin binding to OPN could enable the formation of a bridge to cognate receptors, such as variant exon 3 on CD44.

In vivo, both OPN and CD44 are subject to constant modification by kinases, proteases, glycosidases or glycosyl transferases, and others. This can lead to differential receptor-ligand interactions. Therefore, a linear extrapolation from the test tube results obtained here to the in situ interactions would be premature. We did test eukaryotically produced OPN (obtained from RayBiotech and BioLegend respectively). Neither of them displayed interactions with CD44 or integrin α_V_β_3_ (not shown). While the underlying reasons for this are not clear, it is conceivable that glycosylation of OPN could prevent its interaction with CD44.

Besides the wide diversity of OPN and CD44 proteins (generated by splicing, glycosylation, phosphorylation and others), their potential shared interaction partner heparin, as well as hyaluronate and the integrin α_V_β_3_ generate numerous possibilities for interactions. While it is attractive to hypothesize that distinct combinations could trigger quantitatively or qualitatively unique signals to recipient cells, further experimental work is required to elucidate such possibilities.

## Figures and Tables

**Table 1:** Binding characteristics for OPN interactions with its receptors. CD44 or integrin α_V_β_3_ were immobilized on the ELISA plate at the indicated concentrations, and GST-OPNa was titrated for binding as specified. Plated GST-OPN served to generate a standard curve, which allowed the conversion of absorbance values into ng OPN bound. From double-reciprocal plots, the half-maximal binding concentrations and maximal binding were calculated.

[aVb3] (ng)	[CD44] (ng)	[OPN] (ng)	Half-Maximal (ng)	Maximal (ng)	
	0–1000	125	95.48	99.01	
	0–1000	250	60.93	83.33	
	0–1000	500	107.08	116.28	
	0–1000	1000	150.83	156.25	
	0–1000	2000	122.91	181.82	
	0–1000	4000	138.46	256.41	
	65	125–500	0.77	42.37	
	125	125–500	8.09	55.87	
	250	125–500	40.50	91.74	
	500	125–500	50.78	104.17	
	1000	125–500	60.28	116.28	
200		125–1000	156.25	416.67	
200		7.8–500	433.3	526.32	(OPNa)
			501.6	588.24	(OPNb)
			314	454.55	(OPNc)
31.5–500		500	317.16	400	(OPNa)

## Data Availability

Data are available upon request.

## Supplementary Materials

The following are available online at https://www.mdpi.com/article/10.3390/biom11060813/s1, Supplement Figure S1: CD44 aggregation [37]. Supplement Figure S2: Schematic of surface plasmon resonance. Supplement Figure S3: Salt dependence of binding. Supplement Figure S4: Densitometry of the Pull-down experiments. Supplement Table S1: Literature evidence for interactions between OPN and CD44.
