# Supplementary material for: Structural Constraint of Osteopontin Facilitates Efficient Binding to CD44
Source: Biomolecules. 2021 May 30;11(6):813. doi: 10.3390/biom11060813 (PMC8228714; doi:10.3390/biom11060813)
Supplement: Supplementary file 1 [file biomolecules-11-00813-s001.zip › biomolecules-1220867-SI.pdf]

Supplement Figure S1: CD44 aggregation. A) Alignment of CD44 sequences. LSBio represents the protein used in these experiments. It aligns with the extracellular portion of transcript variant 2. MBS indicates the shorter version of the receptor that was obtained from MyBiosource. The reference sequences (accession number, followed by transcript variant in parentheses) were retrieved from NCBI nucleotide. The hyaluronate binding sites were previously mapped to aa 21-45 (critical residue R41) and aa 144-169 (critical residues K158 and R162) [37]. OPN binding likely occurs downstream. The transmembrane and cytosolic sequence entails WLIILASLLALALILAVCIAVNSRRRCGQKKKLVINSGNGAVEDRKPSGLNGEASKSQEMVHLVNKESSET PDQFMTADETRNLQNVDMKIGV. It is not of relevance here. **B)** CD44v aggregates upon storage. (left) anti-His-tag Western blot of aggregated CD44v, (middle) anti-His-tag Western blot of CD44v, (right) Coomassie stained gel.

Figure S1A

```
CLUSTAL O(1.2.4) multiple sequence alignment

NM_001202556.2(v7)      MDKFWWHAAWGLCLVPLSLAQIDLNITCRFAGVFHVEKNGRYSISRTEAADLCKAFNSTL   60
NM_001001391.2(v4)      MDKFWWHAAWGLCLVPLSLAQIDLNITCRFAGVFHVEKNGRYSISRTEAADLCKAFNSTL   60
NM_001202557.2(v8)      MDKFWWHAAWGLCLVPLSLAQIDLNITCRFAGVFHVEKNGRYSISRTEAADLCKAFNSTL   60
NM_001001390.2(v3)      MDKFWWHAAWGLCLVPLSLAQIDLNITCRFAGVFHVEKNGRYSISRTEAADLCKAFNSTL   60
NM_001202555.2(v6)      MDKFWWHAAWGLCLVPLSLAQIDLNITCRFAGVFHVEKNGRYSISRTEAADLCKAFNSTL   60
NM_000610.4(v1)         MDKFWWHAAWGLCLVPLSLAQIDLNITCRFAGVFHVEKNGRYSISRTEAADLCKAFNSTL   60
NM_001001389.2(v2)      MDKFWWHAAWGLCLVPLSLAQIDLNITCRFAGVFHVEKNGRYSISRTEAADLCKAFNSTL   60
LSBio                   --------------------QIDLNITCRFAGVFHVEKNGRYSISRTEAADLCKAFNSTL   40
MBS                     --------------------QIDLNITCRFAGVFHVEKNGRYSISRTEAADLCKAFNSTL   40
                                            ****************************************

NM_001202556.2(v7)      PTMAQMEKALSIGFETCRYGFIEGHVVIPRIHPNSICAANNTGVYILTSNTSQYDTYCFN   120
NM_001001391.2(v4)      PTMAQMEKALSIGFETCRYGFIEGHVVIPRIHPNSICAANNTGVYILTSNTSQYDTYCFN   120
NM_001202557.2(v8)      PTMAQMEKALSIGFETCRYGFIEGHVVIPRIHPNSICAANNTGVYILTSNTSQYDTYCFN   120
NM_001001390.2(v3)      PTMAQMEKALSIGFETCRYGFIEGHVVIPRIHPNSICAANNTGVYILTSNTSQYDTYCFN   120
NM_001202555.2(v6)      PTMAQMEKALSIGFETCRYGFIEGHVVIPRIHPNSICAANNTGVYILTSNTSQYDTYCFN   120
NM_000610.4(v1)         PTMAQMEKALSIGFETCRYGFIEGHVVIPRIHPNSICAANNTGVYILTSNTSQYDTYCFN   120
NM_001001389.2(v2)      PTMAQMEKALSIGFETCRYGFIEGHVVIPRIHPNSICAANNTGVYILTSNTSQYDTYCFN   120
LSBio                   PTMAQMEKALSIGFETCRYGFIEGHVVIPRIHPNSICAANNTGVYILTSNTSQYDTYCFN   100
MBS                     PTMAQMEKALSIGFETCRYGFIEGHVVIPRIHPNSICAANNTGVYILTSNTSQYDTYCFN   100
                        ************************************************************

NM_001202556.2(v7)      ASAPPEEDCTSVTDLPNAFDGPITITIVNRDGTRYVQKGEYRTNPEDIYPSNPTDDDVSS   180
NM_001001391.2(v4)      ASAPPEEDCTSVTDLPNAFDGPITITIVNRDGTRYVQKGEYRTNPEDIYPSNPTDDDVSS   180
NM_001202557.2(v8)      ASAPPEEDCTSVTDLPNAFDGPITITIVNRDGTRYVQKGEYRTNPEDIYPSNPTDDDVSS   180
NM_001001390.2(v3)      ASAPPEEDCTSVTDLPNAFDGPITITIVNRDGTRYVQKGEYRTNPEDIYPSNPTDDDVSS   180
NM_001202555.2(v6)      ASAPPEEDCTSVTDLPNAFDGPITITIVNRDGTRYVQKGEYRTNPEDIYPSNPTDDDVSS   180
NM_000610.4(v1)         ASAPPEEDCTSVTDLPNAFDGPITITIVNRDGTRYVQKGEYRTNPEDIYPSNPTDDDVSS   180
NM_001001389.2(v2)      ASAPPEEDCTSVTDLPNAFDGPITITIVNRDGTRYVQKGEYRTNPEDIYPSNPTDDDVSS   180
LSBio                   ASAPPEEDCTSVTDLPNAFDGPITITIVNRDGTRYVQKGEYRTNPEDIYPSNPTDDDVSS   160
MBS                     ASAPPEEDCTSVTDLPNAFDGPITITIVNRDGTRYVQKGEYRTNPEDIYPSNPTDDDVSS   160
                        ************************************************************
```

```
NM_001202556.2(v7)      GSSSERSSTSGGYIFYTFSTVHPIPDEDSPWITDSTDRIPATR-----------------  222
NM_001001391.2(v4)      GSSSERSSTSGGYIFYTFSTVHPIPDEDSPWITDSTDRIPAT------------------  222
NM_001202557.2(v8)      GSSSERSSTSGGYIFYTFSTVHPIPDEDSPWITDSTDRIPAT------------------  222
NM_001001390.2(v3)      GSSSERSSTSGGYIFYTFSTVHPIPDEDSPWITDSTDRIPATN-----------------  223
NM_001202555.2(v6)      GSSSERSSTSGGYIFYTFSTVHPIPDEDSPWITDSTDRIPAT------------------  222
NM_000610.4(v1)         GSSSERSSTSGGYIFYTFSTVHPIPDEDSPWITDSTDRIPATTLMSTSATATETATKRQE  240
NM_001001389.2(v2)      GSSSERSSTSGGYIFYTFSTVHPIPDEDSPWITDSTDRIPATS-----------------  223
LSBio                   GSSSERSSTSGGYIFYTFSTVHPIPDEDSPWITDSTDRIPATS-----------------  203
MBS                     GSSSERSSTSGGYIFYTFSTVHPIPDEDSPWITDSTDRIPVD------------------  202
                        ****************************************.

NM_001202556.2(v7)      ------------------------------------------------------------  222
NM_001001391.2(v4)      ------------------------------------------------------------  222
NM_001202557.2(v8)      ------------------------------------------------------------  222
NM_001001390.2(v3)      ------------------------------------------------------------  223
NM_001202555.2(v6)      ------------------------------------------------------------  222
NM_000610.4(v1)         TWDWFSWLFLPSESKNHLHTTTQMAGTSSNTISAGWEPNEENEDERDRHLSFSGSGIDDD  300
NM_001001389.2(v2)      --------------------------TSSNTISAGWEPNEENEDERDRHLSFSGSGIDDD  257
LSBio                   --------------------------TSSNTISAGWEPNEENEDERDRHLSFSGSGIDDD  237
MBS                     ------------------------------------------------------------  202

NM_001202556.2(v7)      ------------------------------------------------------------  222
NM_001001391.2(v4)      ------------------------------------------------------------  222
NM_001202557.2(v8)      ------------------------------------------------------------  222
NM_001001390.2(v3)      ------------------------------------------------------------  223
NM_001202555.2(v6)      ------------------------------------------------------------  222
NM_000610.4(v1)         EDFISSTISTTPRAFDHTKQNQDWTQWNPSHSNPEVLLQTTTRMTDVDRNGTTAYEGNWN  360
NM_001001389.2(v2)      EDFISSTISTTPRAFDHTKQNQDWTQWNPSHSNPEVLLQTTTRMTDVDRNGTTAYEGNWN  317
LSBio                   EDFISSTISTTPRAFDHTKQNQDWTQWNPSHSNPEVLLQTTTRMTDVDRNGTTAYEGNWN  297
MBS                     ------------------------------------------------------------  202

NM_001202556.2(v7)      ------------------------------------------------------------  222
NM_001001391.2(v4)      ------------------------------------------------------------  222
NM_001202557.2(v8)      ------------------------------------------------------------  222
NM_001001390.2(v3)      ------------------------------------------------------------  223
NM_001202555.2(v6)      ------------------------------------------------------------  222
NM_000610.4(v1)         PEAHPPLIHHEHHEEEETPHSTSTIQATPSSTTEETATQKEQWFGNRWHEGYRQTPKEDS  420
NM_001001389.2(v2)      PEAHPPLIHHEHHEEEETPHSTSTIQATPSSTTEETATQKEQWFGNRWHEGYRQTPKEDS  377
LSBio                   PEAHPPLIHHEHHEEEETPHSTSTIQATPSSTTEETATQKEQWFGNRWHEGYRQTPREDS  357
MBS                     ------------------------------------------------------------  202

NM_001202556.2(v7)      ------------------------------------------------------------  222
NM_001001391.2(v4)      ------------------------------------------------------------  222
NM_001202557.2(v8)      ------------------------------------------------------------  222
NM_001001390.2(v3)      ----------------------------------------------------MDSSHSIT  231
NM_001202555.2(v6)      ------------------------------------------------------------  222
NM_000610.4(v1)         HSTTGTAAASAHTSHPMQGRTTPSPEDSSWTDFFNPISHPMGRGHQAGRRMDMDSSHSIT  480
NM_001001389.2(v2)      HSTTGTAAASAHTSHPMQGRTTPSPEDSSWTDFFNPISHPMGRGHQAGRRMDMDSSHSIT  437
LSBio                   HSTTGTAAASAHTSHPMQGRTTPSPEDSSWTDFFNPISHPMGRGHQAGRRMDMDSSHSTT  417
MBS                     ------------------------------------------------------------  202

NM_001202556.2(v7)      ------------------------------------------------------------  222
NM_001001391.2(v4)      ------------------------------------------------------------  222
NM_001202557.2(v8)      ------------------------------------------------------------  222
NM_001001390.2(v3)      LQPTANPNTGLVEDLDRTGPLSMTTQQSNSQSFSTSHEGLEEDKDHPTTSTLTSSNRNDV  291
NM_001202555.2(v6)      -------------------------------------------------------NRNDV  227
NM_000610.4(v1)         LQPTANPNTGLVEDLDRTGPLSMTTQQSNSQSFSTSHEGLEEDKDHPTTSTLTSSNRNDV  540
NM_001001389.2(v2)      LQPTANPNTGLVEDLDRTGPLSMTTQQSNSQSFSTSHEGLEEDKDHPTTSTLTSSNRNDV  497
LSBio                   LQPTANPNTGLVEDLDRTGPLSMTTQQSNSQSFSTSHEGLEEDKDHPTTSTLTSSNRNDV  477
MBS                     ------------------------------------------------------------  202
```

```
NM_001202556.2(v7)      ------------------------------------------------------------   222
NM_001001391.2(v4)      ------------------------------------------------------------   222
NM_001202557.2(v8)      ------------------------------------------------------------   222
NM_001001390.2(v3)      TGGRRDPNHSEGSTTLLEGYTSHYPHTKESRTFIPVTSAKTGSFGVTAVTVGDSNSNVNR   351
NM_001202555.2(v6)      TGGRRDPNHSEGSTTLLEGYTSHYPHTKESRTFIPVTSAKTGSFGVTAVTVGDSNSNVNR   287
NM_000610.4(v1)         TGGRRDPNHSEGSTTLLEGYTSHYPHTKESRTFIPVTSAKTGSFGVTAVTVGDSNSNVNR   600
NM_001001389.2(v2)      TGGRRDPNHSEGSTTLLEGYTSHYPHTKESRTFIPVTSAKTGSFGVTAVTVGDSNSNVNR   557
LSBio                   TGGRRDPNHSEGSTTLLEGYTSHYPHTKESRTFIPVTSAKTGSFGVTAVTVGDSNSNVNR   537
MBS                     ------------------------------------------------------------   202


NM_001202556.2(v7)      -------------------------HSHGSQEGGANTTSGPIRTPQIPEWLIIILASLLAL   258
NM_001001391.2(v4)      ---RDQDTFHPSGGSHTTHGSESDGHSHGSQEGGANTTSGPIRTPQIPEWLIIILASLLAL   279
NM_001202557.2(v8)      ---RDQDTFHPSGGSHTTHGSESDGHSHGSQEGGANTTSGPIRTPQIPEWLIIILASLLAL   279
NM_001001390.2(v3)      SLSGDQDTFHPSGGSHTTHGSESDGHSHGSQEGGANTTSGPIRTPQIPEWLIIILASLLAL   411
NM_001202555.2(v6)      SLSGDQDTFHPSGGSHTTHGSESDGHSHGSQEGGANTTSGPIRTPQIPEWLIIILASLLAL   347
NM_000610.4(v1)         SLSGDQDTFHPSGGSHTTHGSESDGHSHGSQEGGANTTSGPIRTPQIPEWLIIILASLLAL   660
NM_001001389.2(v2)      SLSGDQDTFHPSGGSHTTHGSESDGHSHGSQEGGANTTSGPIRTPQIPEWLIIILASLLAL   617
LSBio                   SLSGDQDTFHPSGGSHTTHGSESDGHSHGSQEGGANTTSGPIRTPQIPE------------   586
MBS                     ------------------------------------------------------------   202


NM_001202556.2(v7)      ALILAVCIAVNSRRRCGQKKKLVINSGNGAVEDRKPSGLNGEASKSQEMVHLVNKESSET   318
NM_001001391.2(v4)      ALILAVCIAVNSRRRCGQKKKLVINSGNGAVEDRKPSGLNGEASKSQEMVHLVNKESSET   339
NM_001202557.2(v8)      ALILAVCIAVNSRRS---------------------------------------------   294
NM_001001390.2(v3)      ALILAVCIAVNSRRRCGQKKKLVINSGNGAVEDRKPSGLNGEASKSQEMVHLVNKESSET   471
NM_001202555.2(v6)      ALILAVCIAVNSRRRCGQKKKLVINSGNGAVEDRKPSGLNGEASKSQEMVHLVNKESSET   407
NM_000610.4(v1)         ALILAVCIAVNSRRRCGQKKKLVINSGNGAVEDRKPSGLNGEASKSQEMVHLVNKESSET   720
NM_001001389.2(v2)      ALILAVCIAVNSRRRCGQKKKLVINSGNGAVEDRKPSGLNGEASKSQEMVHLVNKESSET   677
LSBio                   ------------------------------------------------------------   586
MBS                     ------------------------------------------------------------   202


NM_001202556.2(v7)      PDQFMTADETRNLQNVDMKIGV 340
NM_001001391.2(v4)      PDQFMTADETRNLQNVDMKIGV 361
NM_001202557.2(v8)      ---------------------- 294
NM_001001390.2(v3)      PDQFMTADETRNLQNVDMKIGV 493
NM_001202555.2(v6)      PDQFMTADETRNLQNVDMKIGV 429
NM_000610.4(v1)         PDQFMTADETRNLQNVDMKIGV 742
NM_001001389.2(v2)      PDQFMTADETRNLQNVDMKIGV 699
LSBio                   ---------------------- 586
MBS                     ---------------------- 202
```

Figure S1B

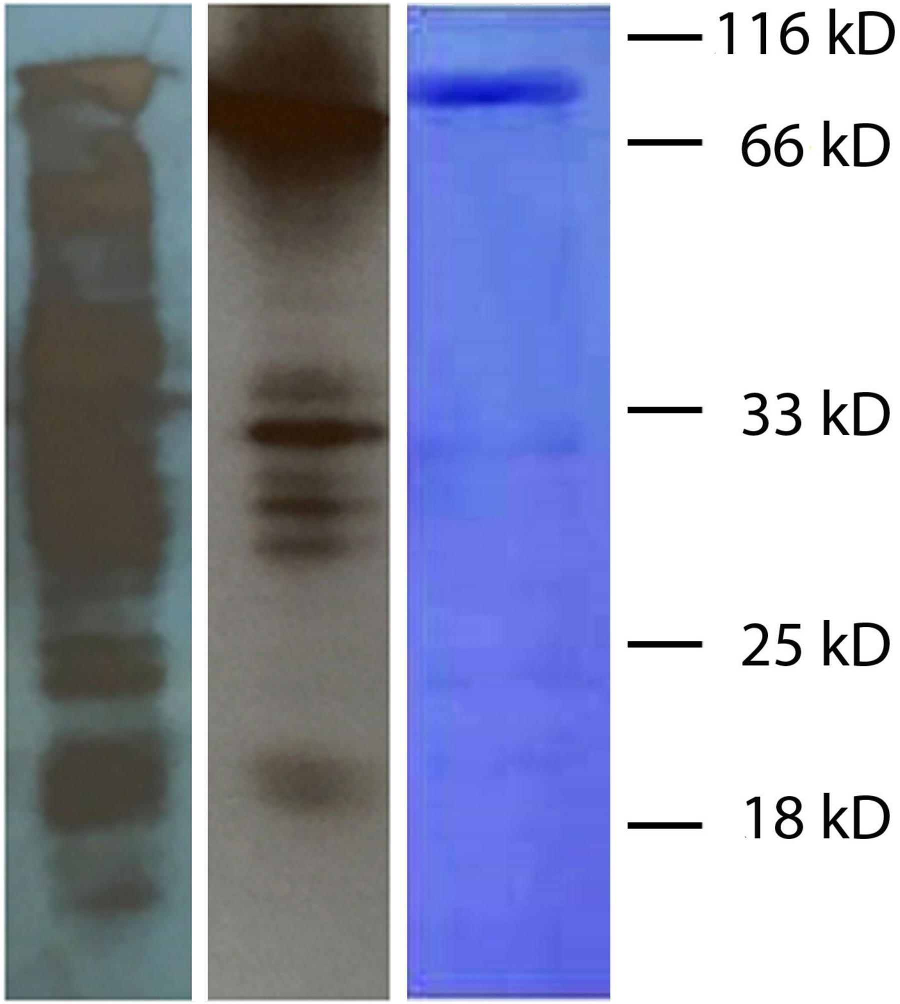

Figure S2

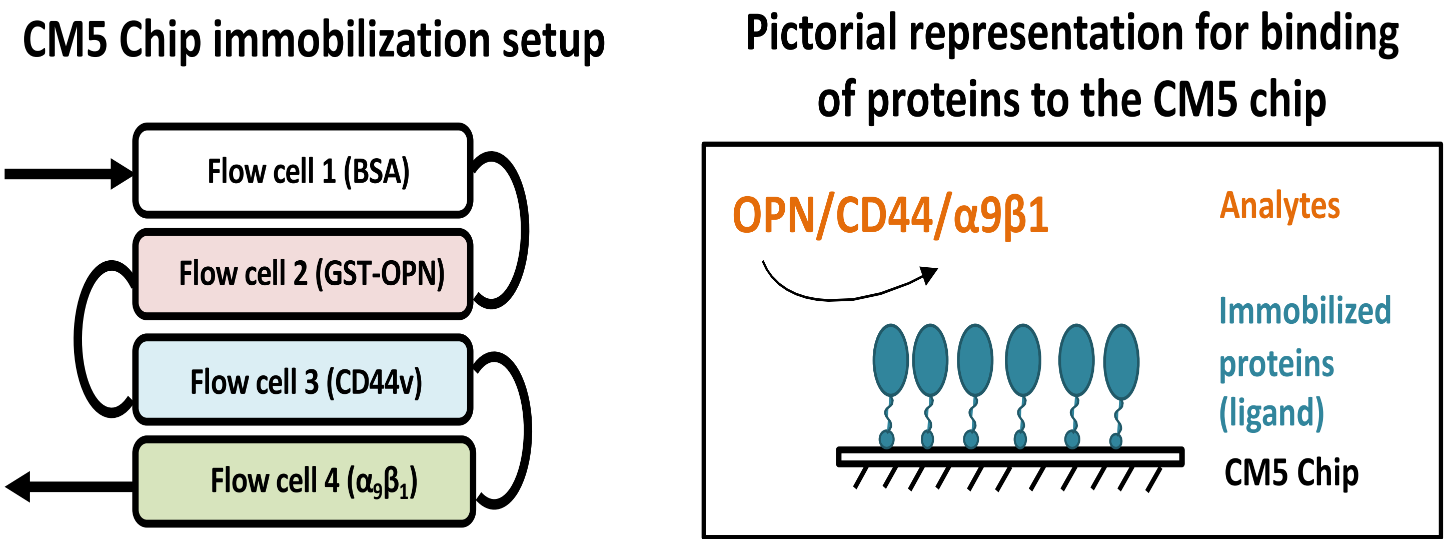

Supplement Figure S2: Schematic of surface plasmon resonance. (left) chip setup with four flow cells/channels (right) permutations of immobilized and flowing binding partners.

Figure S3

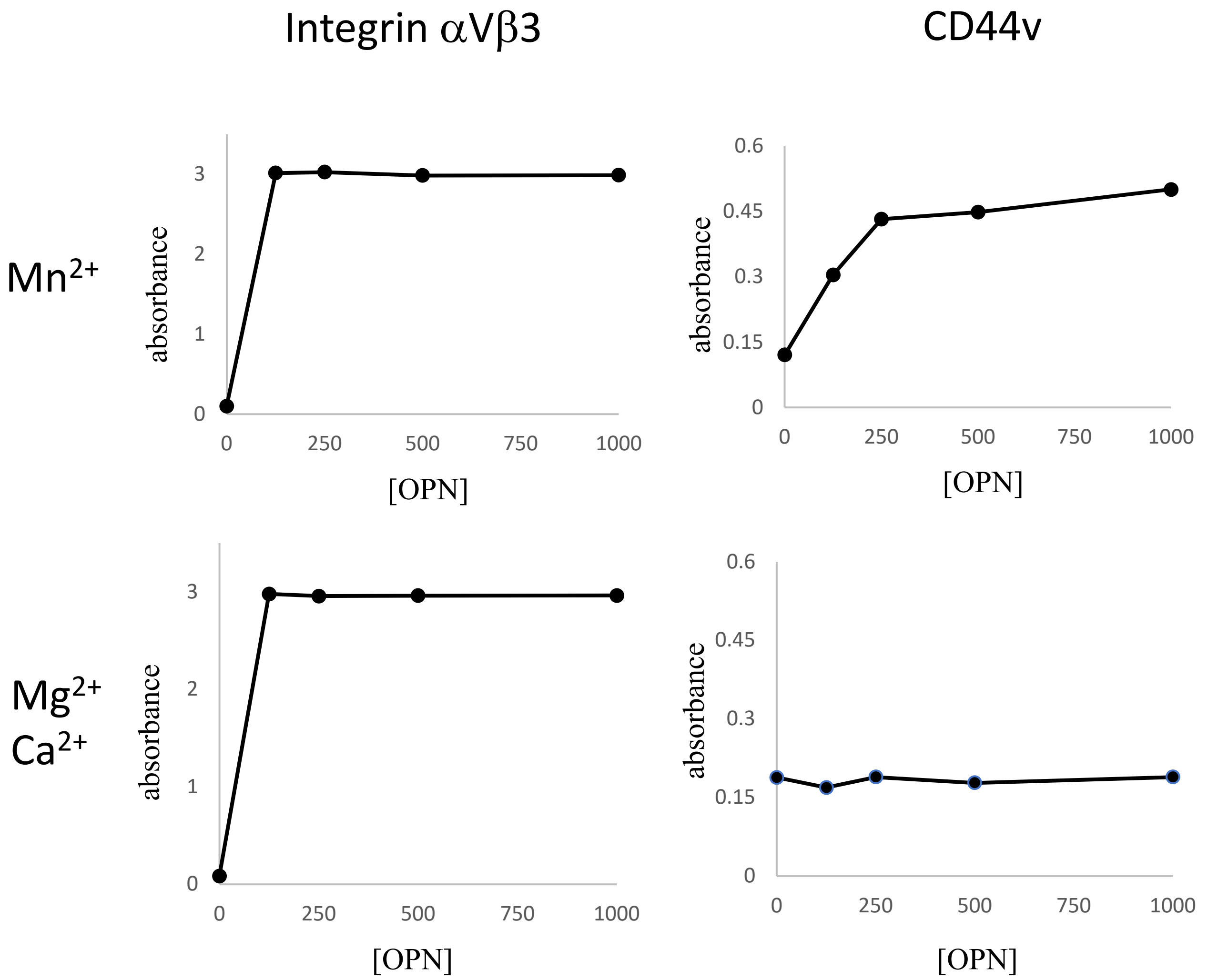

Supplement Figure S3: Salt dependence of binding. Shown are ELISA results for the
interactions between OPN and either Integrin $\alpha_V\beta_3$ or CD44v. Whereas the OPN interaction with the Integrin receptor is inert to the type of divalent cation present in the assay, the interaction with CD44v is supported by 2 mM $MnCl_2$, but not by 2 mM $MgCl_2$ plus 2 mM $CaCl_2$.

B

1	1.2
2	3053.447
3	5000.368
4	6223.589
5	1802.941
6	1645.113
7	20367.29

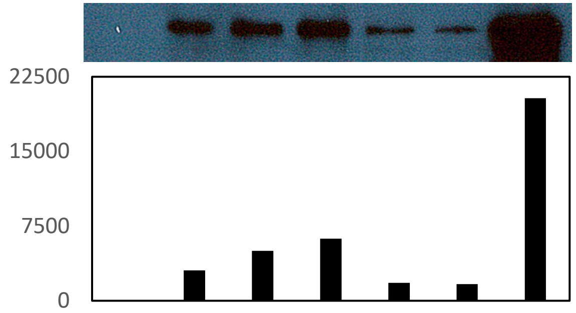

C

1	83.607
2	1559.276
3	1335.640
4	1640.376
5	1526.912
6	1064.912
7	
8	4234.212

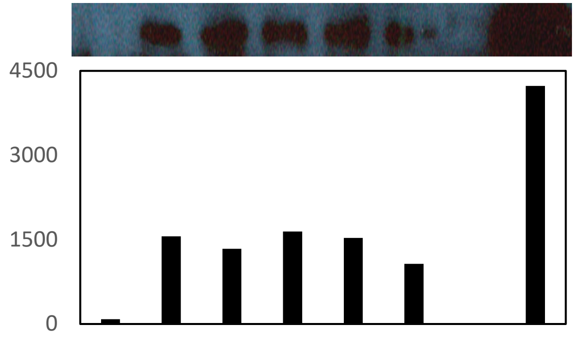

D

1	28.536
2	1656.740
3	1036.719
4	1678.205
5	2012.912
6	690.770
7	
8	3214.024

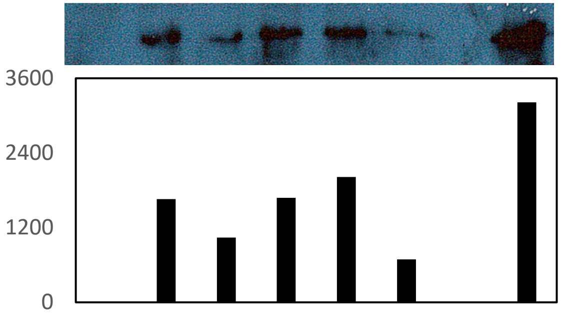

E

1	813.991
2	1352.820
3	1648.083
4	2299.518
5	1792.376
6	2539.690
7	4485.945

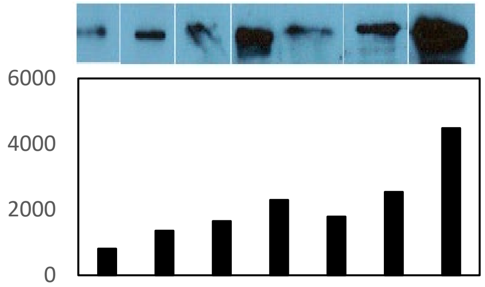

F

1	5540.740
2	6924.640
3	5761.125
4	4527.376
5	5727.811
6	9868.317
7	8484.468
8	4194.619

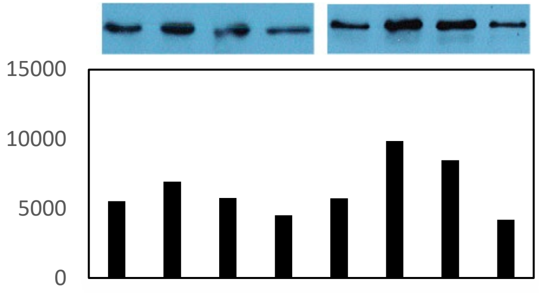

Supplement Figure S4: Densitometry of the Pull-down experiments. Quantitative analysis of Figure 4B-F. Densitometry was performed using ImageJ software.

Supplement Table S1: Literature evidence for interactions between OPN and CD44. A PubMed search for the key words “osteopontin” and “cd44” yielded 416 hits from 1996 through 2019. The papers were reviewed for those that reported **A)** Direct interactions between the ligand-receptor pair, **B)** Co-expression of OPN and CD44 implying functional convergence, **C)** Induction of CD44 expression in target cells by OPN, regardless of their receptor-ligand binding. The tables reflect identification of the source publication, characterization of OPN, characterization of CD44, the cell lines and species of the study, the biological effect induced and its underlying mechanism, and the condition under investigation. Table A) also contains information on identified OPN domains and on Integrin involvement where available.

Table S1A

author	title	journal	OPN	OPN domain	CD44	integrin	cells	species	properties	effect	condition
Zohar R, Cheifetz S, McCulloch CA, Sodek J.	Analysis of intracellular osteopontin as a marker of osteoblastic cell differentiation and mesenchymal cell migration	Eur J Oral Sci. 1998 Jan;106 Suppl 1:401-7	expressed during differentiation, secreted and intracellular		expressed during differentiation		fetal calvarial cells	rat	intracellular, perimembraneous OPN colocalizes with CD44	osteogenic differentiation	bone development
Kusuyama J, Bandow K, Ohnishi T, Hisadome M, Shima K, Semba I, Matsuguchi T.	Osteopontin inhibits osteoblast responsiveness through the down-regulation of focal adhesion kinase mediated by the induction of low-molecular weight protein tyrosine phosphatase	Mol Biol Cell. 2017 May 15;28(10):1326-1336	overexpression, recombinant OPN; OPN-specific siRNA, OPN antibody		suppression by anti-CD44	no effect by cyclic RGD peptide or anti-CD29	MC3T3-E1 cells, primary osteoblasts	mouse	CD44 was a specific OPN receptor for LWW-PTP induction	inhibition of osteoblast responsiveness	bone lesions
Chellaiah MA, Hruska KA.	The integrin alpha(v)beta(3) and CD44 regulate the actions of osteopontin on osteoclast motility.	Calcif Tissue Int. 2003 Mar;72(3):197-205.	secreted from the basolateral surfaces		anti-CD44 blocked stimulation of motility	OPN effect was mimicked by RGD peptides and blocked by anti-αvβ3 antibody	osteoclast	chicken		migration	bone remodeling
Wang M, Guo Y, Zhou Y, Yuan W, Li H, Xiong S, Wang K, Zeng G, Huang Q.	Secreted-Osteopontin Contributes to Brown Adipogenesis In Vitro via a CD44-Dependent Pathway.	Horm Metab Res. 2019 Nov;51(11):741-748.	sOPN, not rhOPN increases brown adipogenesis	bacterial recombinant is ineffective	neutralization of CD44 partially abrogates the effects of sOPN	not mediated by integrin αvβ3	3T3-L1 cells	mouse		accumulation of lipid droplets	adipogenesis, brown
Weber GF, Ashkar S, Glimcher MJ, Cantor H.	Receptor-ligand interaction between CD44 and osteopontin (Eta-1).	Science. 1996 Jan 26;271(5248):509-12; erratum: Science 9 October 1998: Vol. 282 no. 5387 p. 239.	osteosarcoma-secreted, GST-OPN		CD44v3-v6	inhibited by CD44 antibody but not by integrin β3 antibody or GRGDS peptides	transfected fibroblast A31	mouse	calcium-independent, not reversed by chondroitinase treatment of cells	adhesion	cancer
Fedarko NS, Fohr B, Robey PG, Young MF, Fisher LW.	Factor H binding to bone sialoprotein and osteopontin enables tumor cell evasion of complement-mediated attack.	J Biol Chem. 2000 Jun 2;275(22):16666-72.	rOPN complexed with Factor H		endogenous		erythroleukemia cells, breast cancer cells, myeloma cells	mouse, human	pretreatment with hyaluronan or anti-CD44 antibody reduced the protective effect of rOPN	protection from complement attack	cancer
Teramoto H, Castellone MD, Malek RL, Letwin N, Frank B, Gutkind JS, Lee NH.	Autocrine activation of an osteopontin-CD44-Rac pathway enhances invasion and transformation by H-RasV12.	Oncogene. 2005 Jan 13;24(3):489-501.	induced by RAS mutants		upregulated	RGD-independent activity	NIH3T3	mouse	inhibited by the CD44 blocking antibody	foci formation, cell invasion and Rac activity	cancer
Yang G, Zhang S, Gao F, Liu Z, Lu M, Peng S, Zhang T, Zhang F.	Osteopontin enhances the expression of HOTAIR in cancer cells via IRF1.	Biochim Biophys Acta. 2014 Sep;1839(9):837-48.	recombinant human OPN		reversal by CD44 siRNA		Sk-Hep-1, SGC7901, Hela, A549, HCT116, UACC903, PC3, CNE2	homo sapiens	OPN-->CD44-->PI-3K/AKT-->IRF attenuation-->HOTAIR	invasion and metastasis	cancer
Luo X, Ruhland MK, Pazolli E, Lind AC, Stewart SA.	Osteopontin stimulates preneoplastic cellular proliferation through activation of the MAPK pathway.	Mol Cancer Res. 2011 Aug;9(8):1018-29.	recombinant human OPN		inhibition by anti-CD44	no effect by RGD peptide	cancer-associated and senescent fibroblasts, BJ, HaCAT	homo sapiens	activation of the MAPK pathway	preneoplastic keratinocyte cellular proliferation and cell survival	cancer,early transformation
Ahmed M, Sottnik JL, Dancik GM, Sahu D, Hansel DE, Theodorescu D, Schwartz MA.	An Osteopontin/CD44 Axis in RhoGDI2-Mediated Metastasis Suppression.	Cancer Cell. 2016 Sep 12;30(3):432-443.	macrophage-secreted; correlates with tumor aggressiveness, poor outcome		CD44s on the tumor cells		UMUC3	homo sapiens	OPN-CD44-TIAM1-Rac1 axis is a RhoGDI2-sensitive pathway	invasion, growth	cancer, bladder
Jijiwa M, Demir H, Gupta S, Leung C, Joshi K, Orozco N, Huang T, Yildiz VO, Shibahara I, de Jesus JA, Yong WH, Mischel PS, Fernandez S, Kornblum HI, Nakano I.	CD44v6 regulates growth of brain tumor stem cells partially through the AKT-mediated pathway.	PLoS One. 2011;6(9):e24217.	exogenous		poor prognosis associated with high CD44v6		cancer stem cells	mouse, human	OPN increased phosphorylated AKT in CD44(high) GBM, but not in CD44(low) GBM	growth	cancer, brain
Kijewska M, Kocyk M, Kloss M, Stepniak K, Korwek Z, Polakowska R, Dabrowski M, Gieryng A, Wojtas B, Ciechomska IA, Kaminska B.	The embryonic type of SPP1 transcriptional regulation is re-activated in glioblastoma.	Oncotarget. 2017 Mar 7;8(10):16340-16355.	expression of splice variants, siRNA		endogenous		T98G, LN18, LN229, U87 MG, U373	rat, human	reversal of OPN effect by CD44 knockdown, loss in OPN C-terminal truncation	sphere formation	cancer, brain, glioblastoma
Pietras A, Katz AM, Ekström EJ, Wee B, Halliday JJ, Pitter KL, Werbeck JL, Amankulor NM, Huse JT, Holland EC.	Osteopontin-CD44 signaling in the glioma perivascular niche enhances cancer stem cell phenotypes and promotes aggressive tumor growth.	Cell Stem Cell. 2014 Mar 6;14(3):357-69.	endogenous, exogenous		effects were mediated via the γ-secretase-regulated intracellular CD44 domain		DF-1 glioma, U251, T98G	mouse	signaling via CBP/p300-dependent enhancement of HIF-2α activity	cancer stem cell phenotype and aggressive tumor growth in vivo	cancer, brain, glioma
Gao C, Mi Z, Guo H, Kuo PC.	Osteopontin regulates ubiquitin-dependent degradation of Stat1 in murine mammary epithelial tumor cells.	Neoplasia. 2007 Sep;9(9):699-706.	siRNA, transfection		endogenous		4T1	mouse	blocking antibody to CD44 reversed the effect	OPN regulated Ubiquitin-dependent Stat1 degradation through CD44	cancer, breast
Mi Z, Guo H, Russell MB, Liu Y, Sullenger BA, Kuo PC.	RNA aptamer blockade of osteopontin inhibits growth and metastasis of MDA-MB231 breast cancer cells.	Mol Ther. 2009 Jan;17(1):153-61.	aptamer inhibition		endogenous, transfected CD44s	OPN-R3 ablated OPN binding to its receptors CD44 and αvβ3 integrin	MDA-MB-231	homo sapiens	inhibition by OPN aptamer, co-localization of CD44/OPN by FRET	adhesion, migration, invasion	cancer, breast
Chu JE, Xia Y, Chin-Yee B, Goodale D, Croker AK, Allan AL.	Lung-derived factors mediate breast cancer cell migration through CD44 receptor-ligand interactions in a novel ex vivo system for analysis of organ-specific soluble proteins.	Neoplasia. 2014 Feb;16(2):180-91.	in lung-conditioned medium		on cancer cells		MDA-MB-231,MDA-MB-468, SUM149, SUM159	human	pretreatment of cells with a CD44-blocking antibody abrogated migration	migration and proliferation	cancer, breast

Sharon Y, Raz Y, Cohen N, Ben-Shmuel A, Schwartz H, Geiger T, Erez N.	Tumor-derived osteopontin reprograms normal mammary fibroblasts to promote inflammation and tumor growth in breast cancer.	Cancer Res. 2015 Mar 15;75(6):963-73.	shOPN		endogenous, reversal by anti-CD44	secreted osteopontin relied on its receptors CD44 and αVβ3 integrin.	Met-1, MCF7, MCF10A, HCC1143, MDA-MB-231, MDA-MB-468	mouse, human	tumor cell-secreted osteopontin activated CAF phenotypes in normal mammary fibroblasts	fibroblast conversion to CAF	cancer, breast
Hu J, Li G, Zhang P, Zhuang X, Hu G.	A CD44v+ subpopulation of breast cancer stem-like cells with enhanced lung metastasis capacity.	Cell Death Dis. 2017 Mar 16;8(3):e2679.	recombinant OPN		CD44v displays higher capacity of lung metastasis than that CD44s		cancer stem cells, MCF10	homo sapiens	CD44v, but not CD44s, responds to OPN in the lung environment	enhanced cancer cell invasiveness, promotion of lung metastasis	cancer, breast
Pio GM, Xia Y, Piaseczny MM, Chu JE, Allan AL.	Soluble bone-derived osteopontin promotes migration and stem-like behavior of breast cancer cells.	PLoS One. 2017 May 12;12(5):e0177640.	bone-derived osteopontin		endogenous, blocking with anti-CD44	CD44 and RGD-dependent integrins facilitated the response			ALDHhiCD44+CD24- breast cancer cells interact with factors in the bone secondary organ microenvironment to facilitate metastasis	migration, tumorsphere-forming ability, colony-forming ability	cancer, breast
Kumar V, Behera R, Lohite K, Karnik S, Kundu GC.	p38 kinase is crucial for osteopontin-induced furin expression that supports cervical cancer progression.	Cancer Res. 2010 Dec 15;70(24):10381-91.	OPN from breast milk		reversal by CD44 siRNA		Hela, SiHa	homo sapiens	Via CD44, OPN regulated p38 phosphorylation that induced NF-κB activation and expression of furin (implicated in HPV processing)	cervical cell motility is induced	cancer, cervix
Harada N, Mizoi T, Kinouchi M, Hoshi K, Ishii S, Shiiba K, Sasaki I, Matsuno S.	Introduction of antisense CD44S CDNA down-regulates expression of overall CD44 isoforms and inhibits tumor growth and metastasis in highly metastatic colon carcinoma cells.	Int J Cancer. 2001 Jan 1;91(1):67-75.	reduced ability of CD44s antisense transfectants to bind OPN		endogenous		colon adenocarcinoma cell line LS174T	homo sapiens	antisense CD44s inhibited the expression of CD44 variants and reduced the ability to bind hyaluronate and osteopontin	tumor growth, metastasis	cancer, colon
Lee JL, Wang MJ, Sudhir PR, Chen GD, Chi CW, Chen JY.	Osteopontin promotes integrin activation through outside-in and inside-out mechanisms: OPN-CD44V interaction enhances survival in gastrointestinal cancer cells.	Cancer Res. 2007 Mar 1;67(5):2089-97.	overexpression		overexpression of CD44v	dependent on CD44 exon v6 or v7, independent of RGD	HT29, AZ521 gastric cancer	hp	mediated via inside-out signaling to integrin activation	increased survival	cancer, gastric
Hsu KH, Tsai HW, Lin PW, Hsu YS, Shan YS, Lu PJ.	Clinical implication and mitotic effect of CD44 cleavage in relation to osteopontin/CD44 interaction and dysregulated cell cycle protein in gastrointestinal stromal tumor.	Ann Surg Oncol. 2010 Aug;17(8):2199-212.			association of CD44 cleavage with mitosis, tumor size, recurrence, high-risk status,poor survival			homo sapiens	OPN and OPN/CD44 interactions were significantly associated with CD44 cleavage		cancer, gastrointestinal stromal tumor
Hsu KH, Tsai HW, Lin PW, Hsu YS, Shan YS, Lu PJ.	Osteopontin expression is an independent adverse prognostic factor in resectable gastrointestinal stromal tumor and its interaction with CD44 promotes tumor proliferation.	Ann Surg Oncol. 2010 Nov;17(11):3043-52.			endogenous		GIST cell lines	homo sapiens	association between OPN and CD44 (proximity ligation assay)	mitosis	cancer, gastrointestinal stromal tumor
Chien CY, Tsai HT, Su LJ, Chuang HC, Shiu LY, Huang CC, Fang FM, Yu CC, Su HT, Chen CH.	Aurora-A signaling is activated in advanced stage of squamous cell carcinoma of head and neck cancer and requires osteopontin to stimulate invasive behavior.	J Orthop Res. 2014 Sep;32(9):1161-6.	osteopontin signaling induced Aurora-A		endogenous		FaDu, SCC4	homo sapiens	anti-CD44 antibodies caused a decrease of mRNA/protein of Aurora-A and ERK1/2 activity, also affected was Aurora-A-elicited cell motility	invasion	cancer, head and neck
Chen CH, Shiu LY, Su LJ, Huang CY, Huang SC, Huang CC, Yin YF, Wang WS, Tsai HT, Fang FM, Chuang WC, Kang HC, Hwang CF.	FLJ10540 is associated with tumor progression in nasopharyngeal carcinomas and contributes to nasopharyngeal cell proliferation, and metastasis via osteopontin/CD44 pathway.	J Transl Med. 2012 May 16;10:93.	OPN overexpressed, correlated with stage T and stage N, had poor 5-year survival		endogenous		NPC parental cells	homo sapiens	OPN-induced induction of FLJ10540 was inhibitable by anti-CD44	growth and motility	cancer, nasopharyngeal carcinoma
Celetti A, Testa D, Staibano S, Merolla F, Guarino V, Castellone MD, Iovine R, Mansueto G, Somma P, De Rosa G, Galli V, Melillo RM, Santoro M.	Overexpression of the cytokine osteopontin identifies aggressive laryngeal squamous cell carcinomas and enhances carcinoma cell proliferation and invasiveness.	Clin Cancer Res. 2005 Nov 15;11(22):8019-27.	expression elevated in all invasive carcinomas		highly expressed		squamous carcinoma cells	homo sapiens	inhibitable by OPN knockout, by anti-CD44	increased proliferation and matrigel invasion	cancer, laryngeal squamous cell carcinoma
Takafuji V, Forgues M, Unsworth E, Goldsmith P, Wang XW.	An osteopontin fragment is essential for tumor cell invasion in hepatocellular carcinoma.	Oncogene. 2007 Sep 27;26(44):6361-71.	OPN splicing (OPN-c) promoted extracellular cleavage by MMP-9, releasing a region of OPN essential for HCC cellular invasion	OPN-5 kDa (residues 167–210)	endogenous	blocking anti-integrin α5β3 had no effect	hepatocellular carcinoma, HEK293, SMMC-7721	homo sapiens	blocked by the addition of small peptides within the region of OPN-5 kD; a blocking anti-CD44 antibody inhibited OPN-5 kDa-induced invasion	invasion	cancer, hepatocellular carcinoma
Yoo BK, Gredler R, Chen D, Santhekadur PK, Fisher PB, Sarkar D.	c-Met activation through a novel pathway involving osteopontin mediates oncogenesis by the transcription factor LSF.	J Hepatol. 2011 Dec;55(6):1317-24.	induced by LSF		endogenous	blockage of CD44, but not integrin αvβ3 inhibited c-Met activation; anti-c-Met antibody immunoprecipitated c-Met and	HepG3, QGY-7703	homo sapiens	under serum-free conditions, treatment of HepG3 cells with OPN increased the association between CD44 and phospho-c-Met	activation of c-MET	cancer, hepatocellular carcinoma
Zhang R, Pan X, Huang Z, Weber GF, Zhang G.	Osteopontin enhances the expression and activity of MMP-2 via the SDF-1/CXCR4 axis in hepatocellular carcinoma cell lines.	PLoS One. 2011;6(8):e23831.	recombinant human osteopontin		endogenous	OPN binding to integrin $\alpha V\beta_3$ and CD44v6	SMMC7721, HepG2	homo sapiens	via the SDF-1/CXCR4 axis	expression and activity of MMP-2	cancer, hepatocellular carcinoma
Phillips RJ, Helbig KJ, Van der Hoek KH, Seth D, Beard MR.	Osteopontin increases hepatocellular carcinoma cell growth in a CD44 dependant manner.	World J Gastroenterol. 2012 Jul 14;18(26):3389-99.	paracrine modulation of proliferation by all OPN variants		endogenous		Huh-7, HepG2	homo sapiens	only CD44 positive cell lines responded; siRNA knockdown of CD44 blocked response	proliferation	cancer, hepatocellular carcinoma

Medico E, Gentile A, Lo Celso C, Williams TA, Gambarotta G, Trusolino L, Comoglio PM.	Osteopontin is an autocrine mediator of hepatocyte growth factor-induced invasive growth.	Cancer Res. 2001 Aug 1;61(15):5861-8.	major transcriptional target for HGF; function inhibited by anti-OPN		endogenous		embryonic liver cells	mouse	HGF, but not EGF, promoted cell adhesion to OPN via CD44; inhibition by antibodies to OPN and CD44	cell scattering	cancer, liver
Shirasaki T, Honda M, Yamashita T, Nio K, Shimakami T, Shimizu R, Nakasyo S, Murai K, Shirasaki N, Okada H, Sakai Y, Sato T, Suzuki T, Yoshioka K	The osteopontin-CD44 axis in hepatic cancer stem cells regulates IFN signaling and HCV replication.	Sci Rep. 2018 Sep 3;8(1):13143.	enhanced HCV replication and suppressed IFN-stimulated gene expression in EpCAM+/CD44+ cancer stem cells		stem cell marker		cancer stem cells, Huh-7	homo sapiens	no OPN effect on EpCAM-/CD44- cells	regulation of IFN signaling and HCV replication	cancer, liver
Takahashi K, Takahashi F, Hirama M, Tanabe KK, Fukuchi Y.	Restoration of CD44S in non-small cell lung cancer cells enhanced their susceptibility to the macrophage cytotoxicity.	Lung Cancer. 2003 Aug;41(2):145-53.	prodcued by macrophages		CD44s expression down-regulated in NSCLC tissue		non-small cell lung cancer	homo sapiens	completely blocked by either anti-OPN or anti-CD44 antibody	macrophage cytotoxicity	cancer, lung
Sun SJ, Wu CC, Sheu GT, Chang HY, Chen MY, Lin YY, Chuang CY, Hsu SL, Chang JT.	Integrin β3 and CD44 levels determine the effects of the OPN-a splicing variant on lung cancer cell growth.	Oncotarget. 2016 Aug 23;7(34):55572-55584.	OPN-a most a bundant in lung cancer		endogenous	OPN-a inhibited growth of cells with high integrin β3, increased growth via CD44 in cells with low integrin β3 levels	CL1-5 cells, A549 cells	homo sapiens	OPN-a-induced increase in growth appeared to result from activation of the CD44/NFκB pathway	cell growth	cancer, lung
Chiou J, Chang YC, Tsai HF, Lin YF, Huang MS, Yang CJ, Hsiao M.	Follistatin-like Protein 1 Inhibits Lung Cancer Metastasis by Preventing Proteolytic Activation of Osteopontin.	Cancer Res. 2019 Dec 15;79(24):6113-6125.	upregulated	proteolytically cleaved	CD44-specific antibodies inhibit migration	antibodies to integrin $\alpha_V\beta_3$ or to CD44 inhibit the increase in migration/invasion	A549, H1299, PC13, PC14	homo sapiens, mouse	FSTL1 binds to uncleaved osteopontin, restraining its proteolytic activation, leading to inactivation of integrin/CD44-associated signaling	rearrangement of the actin cytoskeleton	cancer, lung
Caers J, Günthert U, De Raeve H, Van Valckenborgh E, Menu E, Van Riet I, Van Camp B, Vanderkerken K.	The involvement of osteopontin and its receptors in multiple myeloma cell survival, migration and invasion in the murine 5T33MM model.	Br J Haematol. 2006 Feb;132(4):469-77.	expressed by multiple myeloma cells		expression of CD44v by multiple myeloma cells	proliferation inhibitable by anti-CD44v6 antibodies, cell migration inhibitable by anti-CD44v and RGD peptides	5T33MM	mouse		proliferation, migration	cancer, multiple myeloma
Katagiri YU, Sleeman J, Fujii H, Herrlich P, Hotta H, Tanaka K, Chikuma S, Yagita H, Okumura K, Murakami M, Saiki I, Chambers AF, Uede T.	CD44 variants but not CD44s cooperate with beta1-containing integrins to permit cells to bind to osteopontin independently of arginine-glycine-aspartic acid, thereby stimulating cell motility and chemotaxis.	Cancer Res. 1999 Jan 1;59(1):219-26.	GST-OPN (murine)	binding to both the amino- and carboxy-terminal portions of OPN	CD44v6 or CD44v7 but not CD44s	RGD-independent; cooperation required with integrin β1, but not intergin β3	pancreatic cancer cell line	rat		motility is induced	cancer, pancreas
Castellone MD, Celetti A, Guarino V, Cirafici AM, Basolo F, Giannini R, Medico E, Kruhoffer M, Orntoft TF, Curcio F, Fusco A, Melillo RM, Santoro M.	Autocrine stimulation by osteopontin plays a pivotal role in the expression of the mitogenic and invasive phenotype of RET/PTC-transformed thyroid cells.	Oncogene. 2004 Mar 18;23(12):2188-96.	induced by the oncogene RET-PTC		overexpression induced by the oncogene RET-PTC		thyroid follicular cells	mouse	impaired by OPN- and CD44-blocking antibodies	proliferation, Matrigel invasion, spreading in collagen gels	cancer, papillary thyroid carcinoma
Robertson BW, Bonsal L, Chellaiah MA.	Regulation of Erk1/2 activation by osteopontin in PC3 human prostate cancer cells.	Mol Cancer. 2010 Sep 26;9:260.	transfection/overexpression		endogenous	OPN induced activation of Akt through both αVβ3 integrins and CD44	PC-3	human			cancer, prostate
Erb U, Megaptche AP, Gu X, Büchler MW, Zöller M	CD44 standard and CD44v10 isoform expression on leukemia cells distinctly influences niche embedding of hematopoietic stem cells.	J Hematol Oncol. 2014 Mar 31;7:29.	EL4-v10 catches OPN		CD44v10		EL-4	mouse	blocking CD44v10-stimulated osteopontin expression that could drive HSC out of the niche	migration to marrow stroma	cancer, thymoma
Megaptche AP, Erb U, Büchler MW, Zöller M.	CD44v10, osteopontin and lymphoma growth retardation by a CD44v10-specific antibody.	Immunol Cell Biol. 2014 Sep;92(8):709-20.			CD44v10		EL-4	mouse	CD44v10 binding of OPN drives leukocyte recruitment and activation	leukocyte recruitment and activation	cancer, thymoma
Guarino V, Faviana P, Salvatore G, Castellone MD, Cirafici AM, De Falco V, Celetti A, Giannini R, Basolo F, Melillo RM	Osteopontin is overexpressed in human papillary thyroid carcinomas and enhances thyroid carcinoma cell invasiveness.	J Clin Endocrinol Metab. 2005 Sep;90(9):5270-8.	overexpressed in most cases; correlated with lymph node metastases and tumor size		overexpressed in most papillary thyroid carcinomas				blockage with anti-CD44 antibodies prevented OPN-induced matrigel invasion	invasiveness	cancer, thyroid carcinoma
Kim EK, Jeon I, Seo H, Park YJ, Song B, Lee KA, Jang Y, Chung Y, Kang CY.	Tumor-derived osteopontin suppresses antitumor immunity by promoting extramedullary myelopoiesis.	Cancer Res. 2014 Nov 15;74(22):6705-16.	recombinant mOPN, OPN expressed by tumor cells		endogenous		myeloid-derived cells	mouse	OPN enhances extramedullary myelopoiesis in a CD44-dependent manner	extramedullary myelopoiesis	immunosuppression, tumor-dependent
Klement JD, Paschall AV, Redd PS, Ibrahim ML, Lu C, Yang D, Celis E, Abrams SI, Ozato K, Liu K.	An osteopontin/CD44 immune checkpoint controls CD8+ T cell activation and tumor immune evasion.	J Clin Invest. 2018 Dec 3;128(12):5549-5560.	IRF8 functioned as a repressor of OPN in myeloid cells		IRF8 deficiency increases CD44hiCD8+ memory T cells		T-lymphocytes	mouse, human	IRF8-deficient mice are deficient in generation of antigen-specific CD8+ T cells		immunosuppression, tumor-dependent
Lin YH, Huang CJ, Chao JR, Chen ST, Lee SF, Yen JJ, Yang-Yen HF.	Coupling of osteopontin and its cell surface receptor CD44 to the cell survival response elicited by interleukin-3 or granulocyte-macrophage colony-stimulating factor.	Mol Cell Biol. 2000 Apr;20(8):2734-42; erratum: Mol Cell Biol 2001 Mar;21(6):2248.	wild-type OPN	loss of function in a deletion mutant lacking aa79-140	endogenous		pro-B-lymphocyte	mouse	response blocked by anti-CD44	survival response elicited by IL-3 or GM-CSF	immune system, survival

Lin YH, Yang-Yen HF.	The osteopontin-CD44 survival signal involves activation of the phosphatidylinositol 3-kinase/Akt signaling pathway.	J Biol Chem. 2001 Dec 7;276(49):46024-30.	wild type, mutant	CD44-binding domain contains aa 121-140, threonine 137 and serine 147 are essential	endogenous		pro-B-lymphocyte Ba/F3	mouse	wild-type OPN but not the inactive mutant induced activation of PI 3-kinase and Akt	survival response elicited by IL-3 or GM-CSF	immune system, survival
Sterling H, Saginario C, Vignery A.	CD44 occupancy prevents macrophage multinucleation.	J Cell Biol. 1998 Nov 2;143(3):837-47.	exogenous OPN		CD44 elevated during fusion		macrophage	rat	CD44 ligands (hyaluronic acid, chondroitin sulfates, osteopontin) prevent multinucleation	macrophage fusion	immune system
Weiss JM, Renkl AC, Maier CS, Kimmig M, Liaw L, Ahrens T, Kon S, Maeda M, Hotta H, Uede T. Simon	Osteopontin is involved in the initiation of cutaneous contact hypersensitivity by inducing Langerhans and dendritic cell migration to lymph nodes.	J Exp Med. 2001 Nov 5;194(9):1219-29.	upregulated OPN expression, GST-OPN		endogenous		Langerhans cells/dendritic cells	mouse	independent of divalent cations, inhibition by anti-CD44 antibody	chemotaxis	allergiy, cutaneous contact hypersensitivity
Guan H, Nagarkatti PS, Nagarkatti M.	CD44 Reciprocally regulates the differentiation of encephalitogenic Th1/Th17 and Th2/regulatory T cells through epigenetic modulation involving DNA methylation of cytokine gene promoters, thereby controlling the development of experimental autoimmune	J Immunol. 2011 Jun 15;186(12):6955-64.	osteopontin, but not hyaluronic acid, promoted Th1/Th17 differentiation		CD44 inhibited Th1/Th17 differentiation while enhancing Th2/regulatory T-cell differentiation				signaling through CD44, in encephalitogenic T-cells, triggers Th cell differentiation through regulating DNA methylation of Th1/Th17 and Th2 cytokine genes	Th2/regulatory T-cell differentiation	autoimmune encephalomyelitis
Wittig BM, Sabat R, Holzlöhner P, Witte-Händel E, Heilmann K, Witte K, Triebus J, Tzankov A, Laman JD, Bokemeyer B, Terracciano L, Schwärzler C, Kohler H, Volkmer R, Loddenkemper C, Wolk K, Hoffmann U, Günthert U.	Absence of specific alternatively spliced exon of CD44 in macrophages prevents colitis.	Mucosal Immunol. 2018 May;11(3):846-860.	ligand for CD44v7; levels were elevated in Crohn's disease		CD44v7 absence on macrophages in recipient mice prevented colitis		macrophages	mouse, human		induction of IL-6 in monocytes, a cytokine also increased in these patients	autoimmunity, colitis
Murugaiyan G, Mittal A, Weiner HL.	Increased osteopontin expression in dendritic cells amplifies IL-17 production by CD4+ T cells in experimental autoimmune encephalomyelitis and in multiple sclerosis.	J Immunol. 2008 Dec 1;181(11):7480-8.	polyclonal anti-OPN, recombinant OPN		endogenous	Opn induced IL-17 production by CD4+ T cells via integrin β3; Opn inhibited IL-10 production via the CD44 receptor	dendritic cells	mouse, human		inhibition of IL-10 secretion	autoimmunity
Kim JS, Bashir MM, Werth VP.	Gottron's papules exhibit dermal accumulation of CD44 variant 7 (CD44v7) and its binding partner osteopontin: a unique molecular signature.	J Invest Dermatol. 2012 Jul;132(7):1825-32.	increased OPN compared with healthy skin		CD44v7 upregulated in Gottron's lesions			homo sapiens	OPN did not induce CD44v7, stretching dermal fibroblasts in the presence of OPN increased monocyte binding via CD44v7	autoimmunity, inflammation	dermatomyositis
Chen G, Zhang X, Li R, Fang L, Niu X, Zheng Y, He D, Xu R, Zhang JZ.	Role of osteopontin in synovial Th17 differentiation in rheumatoid arthritis.	Arthritis Rheum. 2010 Oct;62(10):2900-8.	levels correlated with IL-17 production and frequency of Th17 cells; anti-OPN blocked IL-17 production by T cells		OPN-dependent IL17A promoter acetylation mainly via CD44 binding in CD4+ T cells	the IL-17 response specifically involved the OPN receptors CD44 and CD29	mononuclear cells, CD4+ T-cells	homo sapiens	OPN induced H3 acetylation of the IL17A gene promoter, allowing the interaction of the IL17A gene locus with ROR	IL-17 secretion	arthritis, rheumatoid
Cheng C, Zhang FJ, Tian J, Tu M, Xiong YL, Luo W, Li YS, Song BB, Gao SG, Lei GH.	Osteopontin inhibits HIF-2α mRNA expression in osteoarthritic chondrocytes.	Exp Ther Med. 2015 Jun;9(6):2415-2419.	recombinant human OPN, OPN small interfering RNA (siRNA)		endogenous		chondrocyte	homo sapiens	CD44 blocking mAb suppressed the inhibitory effect of OPN on HIF-2α mRNA expression		arthritis, osteo
Weber GF, Zawaideh S, Hikita S, Kumar VA, Cantor H, Ashkar S.	Phosphorylation-dependent interaction of osteopontin with its receptors regulates macrophage migration and activation.	J Leukoc Biol. 2002 Oct;72(4):752-61.	recombinant OPN, phosphorylated by kinases, cleaved by thrombin	N-terminus engages integrin phosphorylation-dependently, C-terminus engages CD44 phosphorylation-independently	endogenous	division of labor between integrin and CD44 receptors for distinct OPN domains	macrophage	mouse	OPN C-terminal domain, phosphorylation-independent	chemotaxis	inflammation, DTH, wound healing
Gao C, Guo H, Wei J, Kuo PC.	Osteopontin inhibits expression of cytochrome c oxidase in RAW 264.7 murine macrophages.	Biochem Biophys Res Commun. 2003 Sep 12;309(1):120-5.	exogenous murine OPN		endogenous	blockage of OPN-integrin binding by GRGDSP had no effect	macrophage RAW 264.7	mouse	effect blocked by the CD44 competitive ligand, hylauronate	inhibited gene expression of cytochrome c oxidase	inflammation
Nagasaka A, Matsue H, Matsushima H, Aoki R, Nakamura Y, Kambe N, Kon S, Uede T, Shimada S.	Osteopontin is produced by mast cells and affects IgE-mediated degranulation and migration of mast cells.	Eur J Immunol. 2008 Feb;38(2):489-99.	spontaneously produced, inducible by ionomycin and FcepsilonRI aggregation		endogenous		mast cells	mouse	neutralizing antibody partially blocks degranulation, completely blocks migration	degranulation, chemotaxis	inflammation
Marcondes MC, Poling M, Watry DD, Hall D, Fox HS.	In vivo osteopontin-induced macrophage accumulation is dependent on CD44 expression.	Cell Immunol. 2008;254(1):56-62.	native OPN and its thrombin-cleaved form		macrophage numbers in CD44-/- reduced within the cavity, but not along subcutaneous lining		macrophage	mouse	loss of function in CD44 knockout mice	leukocyte accumulation	inflammation (subcutaneous air pouch)
Bollyky PL, Wu RP, Falk BA, Lord JD, Long SA, Preisinger A, Teng B, Holt GE, Standifer NE, Braun KR, Xie CF, Samuels PL, Vernon RB, Gebe JA, Wight TN, Nepom GT.	ECM components guide IL-10 producing regulatory T-cell (TR1) induction from effector memory T-cell precursors.	Proc Natl Acad Sci U S A. 2011 May 10;108(19):7938-43.	OPN suppressed TR1 induction		CD44 signaling served as a nexus for TR1 induction		reguatory T-cells		CD44 cross-linking, signaling through p38 and ERK1/2 mediated TR1 induction	blocking of fate decision regarding TR1 induction	inflammation
Iqbal J, McRae S, Mai T, Banaudha K, Sarkar-Dutta M, Waris G.	Role of hepatitis C virus induced osteopontin in epithelial to mesenchymal transition, migration and invasion of hepatocytes.	PLoS One. 2014 Jan 31;9(1):e87464.	parasite infection induced OPN release from macrophages		endogenous	OPN binding to integrin αVβ3 and CD44	macrophages, hepatocytes	mouse	receptor engagement led to activation of FAK, SRC, AKT	migration and invasion	infection HCV

Iqbal J, Sarkar-Dutta M, McRae S, Ramachandran A, Kumar B, Waris G.	Osteopontin Regulates Hepatitis C Virus (HCV) Replication and Assembly by Interacting with HCV Proteins and Lipid Droplets and by Binding to Receptors αVβ3 and CD44.	J Virol. 2018 Jun 13;92(13). pii: e02116-17.	upregulation in HCV-associated HCC; endogenous OPN correlated with HCV proteins (NS3, NS5A, NS4A/B, NS5B, core)		endogenous	critical role of secreted OPN in human hepatoma cell migration/invasion through receptors integrin αVβ3 and CD44	liver cells		reduction in HCV replication, assembly, and infectivity in HCV-infected cells transfected with siRNA against OPN, αVβ3, and CD44	HCV replication	infection hepatitis C
Ashkar S, Weber GF, Panoutsakopoulou V, Sanchirico ME, Jansson M, Zawaideh S, Rittling SR, Denhardt DT, Glimcher MJ, Cantor H.	Eta-1 (osteopontin): an early component of type-1 (cell-mediated) immunity.	Science. 2000 Feb 4;287(5454): 860-4.	endogenous, recombinant	N-terminus engages integrin, C-terminus engages CD44	endogenous, knockout	distinct domains for integrin and CD44	macrophage	mouse	CD44 interaction independent of OPN phosphorylation	IL-10 suppression	infection, intracellular pathogens
Santamaría MH, Corral RS.	Osteopontin-dependent regulation of Th1 and Th17 cytokine responses in Trypanosoma cruzi-infected C57BL/6 mice.	Cytokine. 2013 Feb;61(2):491-8.	anti-OPN antibody lowered Th1 and Th17 responses and increased parasitemia		endogenous	Induction of IFN-γ, IL-12 p70, IL-17A inhibitable by anti-integrin β3, not by anti-CD44; IL-10 suppression inhibitable by anti-CD44, not by anti-integrin β3		mouse		suppression of IL-10 secretion	infection, Trypanosoma cruzi
Zhu Y, Wei Y, Chen J, Cui G, Ding Y, Kohanawa M, Xu X, Diao H.	Osteopontin Exacerbates Pulmonary Damage in Influenza-Induced Lung Injury.	Jpn J Infect Dis. 2015;68(6):467-73.		OPN decreased mRNA expression, promoted MMP-7 expression of ENaC α	endogenous	MMP-7 expression via interaction with integrin β1 and CD44, decreased mRNA expression through integrin β3 and CD44	lung epithelial cells	human		MMP-7 upregulation, epithelial sodium channel downregulation	infection, bacterial lung
Raheja LF, Genetos DC, Yellowley CE.	Hypoxic osteocytes recruit human MSCs through an OPN/CD44-mediated pathway.	Biochem Biophys Res Commun. 2008 Feb 22;366(4):1061-6.	conditioned medium, recombinant OPN		endogenous		osteocytes, MSCs		OPN and CD44 neutralizing antibodies reduced MSC migration.	mesenchymal stem cell migration	hypoxia
Pardo A, Gibson K, Cisneros J, Richards TJ, Yang Y, Becerril C, Yousem S, Herrera I, Ruiz V, Selman M, Kaminski N.	Up-regulation and profibrotic role of osteopontin in human idiopathic pulmonary fibrosis.	PLoS Med. 2005 Sep;2(9):e251.	upregulated, localized to alveolar epithelial cells, elevated in bronchoalveolar lavage		endogenous	epithelial growth inhibited by GRGDS and anti-CD44, fibroproliferation inhibited by GRGDS and anti-αvβ3, migration (by either) inhibited by GRGDS and anti-CD44 and anti-αvβ3	lung fibroblasts, alveolar epithelial cells	homo sapiens		migration and proliferation	lung, idiopathic pulmonary fibrosis
Seo KW, Lee SJ, Ye BH, Kim YW, Bae SS, Kim CD.	Mechanical stretch enhances the expression and activity of osteopontin and MMP-2 via the Akt1/AP-1 pathways in VSMC.	J Mol Cell Cardiol. 2015 Aug;85:13-24.	endogenous, recombinant OPN		endogenous	MMP-2 expression/activity were attenuated with anti-CD44 antibody, but not by a RGD peptide	vascular smooth muscle cells	rat, mouse		MMP-2 expression	cardiovascular, hypertension
Zheng Y, Wang Z, Deng L, Yuan X, Ma Y, Zhang G, Gantier MP, Liu JP, Shen L, Xu D.	Osteopontin promotes inflammation in patients with acute coronary syndrome through its activity on IL-17 producing cells.	Eur J Immunol. 2012 Oct;42(10):2803-14.	direct effect of OPN on T cells		CD4(+) CD45RA(-) FOXP3(lo) T cells	mediated through recruitment of the OPN receptors CD29 and CD44	T-lymphocytes	homo sapiens	The OPN effect is dependent on STAT3 and the RORγt pathway, but not IL-6 production	increased IL-17 production	cardiovascular, acute coronary syndrome
Poggio P, Branchetti E, Grau JB, Lai EK, Gorman RC, Gorman JH 3rd, Sacks MS, Bavaria JE, Ferrari G.	Osteopontin-CD44v6 interaction mediates calcium deposition via phospho-Akt in valve interstitial cells from patients with noncalcified aortic valve sclerosis.	Arterioscler Thromb Vasc Biol. 2014 Sep;34(9):2086-94.	OPN accumulates within the aortic leaflets		CD44v6		valve interstitial cells	mouse, human	osteopontin-CD44 interaction mediated calcium deposition via phospho-Akt	osteogenic transdifferentiation	cardiovascular, aortic valve stenosis
Dalal S, Zha Q, Daniels CR, Steagall RJ, Joyner WL, Gadeau AP, Singh M, Singh K.	Osteopontin stimulates apoptosis in adult cardiac myocytes via the involvement of CD44 receptors, mitochondrial death pathway, and endoplasmic reticulum	Am J Physiol Heart Circ Physiol. 2014 Apr 15;306(8):H1182-91.	OPN increase associated with myocyte apoptosis, myocardial dysfunction		endogenous	OPN co-immunoprecipitated with CD44, not with β1 or β3 integrins.	ventricular myocytes	rat, mouse	Proximity ligation assay; neutralizing anti-CD44 antibodies inhibited OPN-stimulated apoptosis	apoptosis	cardiovascular, myocardial dysfunction
Zheng YH, Tian C, Meng Y, Qin YW, Du YH, Du J, Li HH	Osteopontin stimulates autophagy via integrin/CD44 and p38 MAPK signaling pathways in vascular smooth muscle cells.	J Cell Physiol. 2012 Jan;227(1):127-35.	highest expressed gene in aortic abdominal aneurysm		endogenous	OPN effects abrogated by inhibition of integrin/CD44	vascular smooth muscle cells		p38 MAPK signaling pathways essential for OPN effects	increased formation of autophagosomes, expression of autophagy-related genes, cell death	cardiovacular, aneurysm
Neumann C, Garreis F, Paulsen F, Hammer CM, Birke MT, Scholz M.	Osteopontin is induced by TGF-β2 and regulates metabolic cell activity in cultured human optic nerve head astrocytes.	PLoS One. 2014 Apr 9;9(4):e92762.	OPN is a TGF-β2 responsive factor in optic nerve head astrocytes		CD44, integrins αV, α4, α 5, α6, α9, β1, β3, β5 expressed, unaffected by TGF	OPN signaling via CD44 repressed metabolic activity, signaling via integrin had pro-metabolic effect	optic nerve astrocytes	homo sapiens		metabolic activity	nervous system, glaucoma
Ries A, Goldberg JL, Grimpe B.	A novel biological function for CD44 in axon growth of retinal ganglion cells identified by a bioinformatics approach.	J Neurochem. 2007 Nov;103(4):1	substrate for adhesion		expression in retinal ganglion cells	integrin/CD44 synergy possible to interact with OPN/laminin	retinal ganglion cells	rat	deoxyribozyme to CD44 causes reduced axon initiation of retinal ganglion cells on all substrates	axon growth (additive with laminin)	nervous system, axon growth
Plantman S.	Osteopontin is upregulated after mechanical brain injury and stimulates neurite growth from hippocampal neurons through β1 integrin and CD44.	Neuroreport. 2012 Aug 1;23(11):647-52.				growth on OPN was mediated through β1 intregrins and CD44	hippocampal neurons, cortical astrocytes	rat	injury-induced OPN may support neurite sprouting	neuron growth, formation of primary neurites	nervous system, mechanical brain injury

Ailane S, Long P, Jenner P, Rose S.	Expression of integrin and CD44 receptors recognising osteopontin in the normal and LPS-lesioned rat substantia nigra.	Eur J Neurosci. 2013 Aug;38(3):2468-76.	expressed in the substantia nigra		upregulated expression in LPS-lesioned substantia nigra	integrin αv, β3, β1 and CD44 expressed on neurons; up-regulation of β3, CD44 after lesion	neurons	rat	anti-Igαv, -Igβ1, -Igβ3, -CD44 immunoprecipitated OPN from striatum and substantia nigra	protection of nigral dopaminergic neurons against toxic insult	nervous system, Parkinson's disease
Morisaki Y, Niikura M, Watanabe M, Onishi K, Tanabe S, Moriwaki Y, Okuda T, Ohara S, Murayama S, Takao M, Uchida S, Yamanaka K, Misawa H.	Selective Expression of Osteopontin in ALS-resistant Motor Neurons is a Critical Determinant of Late Phase Neurodegeneration Mediated by Matrix Metalloproteinase-9.	Sci Rep. 2016 Jun 6;6:27354.	accumulated as extracellular granules in ECM; selectively expressed by FR and S motor neurons and ALS-resistant motor pools		expression of CD44 mRNA and protein increased during disease progression	MMP-9 up-regulation via αvβ3 integrin in Neuro2a cells; CD44-mediated astrocyte migration, microglial phagocytosis	astrocytes, Neuro2a, microglia	mouse, human		astrocyte migration and invasion, microglial phagocytosis	nervous system, amyotrophic lateral sclerosis
Seth D, Duly A, Kuo PC, McCaughan GW, Haber PS.	Osteopontin is an important mediator of alcoholic liver disease via hepatic stellate cell activation.	World J Gastroenterol. 2014 Sep 28;20(36):13088-104.	OPN expression correlated with disease severity		CD44v6	increased expression of αvβ3-integrin and CD44, stronger inhibition of LX2 migration by blocking OPN than CD44v6 or αvβ3-integrin	hepatic stellate cells, LX2	mouse, human	reversal by blocking antibody to CD44v6, reversal by siRNA, aptamer, neutralizing antibodies to OPN	plasmin, activation of fibrinolysis is mediated via OPN in hepatic stellate cells	alcoholic liver disease
Chen CH, Cheng CY, Chen YC, Sue YM, Liu CT, Cheng TH, Hsu YH, Chen TH.	MicroRNA-328 inhibits renal tubular cell epithelial-to-mesenchymal transition by targeting the CD44 in pressure-induced renal fibrosis.	PLoS One. 2014 Jun 11;9(6):e99802.	effect reversible by neutralizing antibody		expression up-regulated at the initial pressurization stage		renal tubular cells NRK-52E	rat	hyaluronan binding peptide pep-1 and ant-OPN inhibited pressure-induced EMT	epithelial-mesenchymal transition	renal fibrosis
Sadvakassova G, Dobocan MC, Congote LF.	Osteopontin and the C-terminal peptide of thrombospondin-4 compete for CD44 binding and have opposite effects on CD133+	BMC Res Notes. 2009 Oct 23;2:215.	recombinant human osteopontin		CD44E, CD44v4, CD44R1		skin fibroblasts, 293T	human	C21 thrombospondin fragment and OPN competed for CD44 binding; common binding sites are likely	inhibition of CD133+ cell colony formation	stem cell biology
Zohar R, Suzuki N, Suzuki K, Arora P, Glogauer M, McCulloch CA, Sodek J.	Intracellular osteopontin is an integral component of the CD44-ERM complex involved in cell migration.	J Cell Physiol. 2000 Jul;184(1):118-30.	intracellular OPN, no uptake of exogenous OPN		CD44(cytoplasmic tail)-ERM		fetal fibroblasts, periodontal ligament cells, macrophages, metastatic breast cancer cells	mouse, rat, human	physical association with OPN, CD44, and ERM, but not with vinculin or alpha-actin	cell migration	
Lee JL, Wang MJ, Sudhir PR, Chen JY.	CD44 engagement promotes matrix-derived survival through the CD44-SRC-integrin axis in lipid rafts.	Mol Cell Biol. 2008 Sep;28(18):5710-23.	purified exogenous OPN		present in lipid rafts	enrichment of integrin β1 in lipid rafts is coupled to CD44 ligation-elicited raft reorganization	HT29, AZ521	rat, human	signal transduction through CD44-Src-integrin axis	matrix-derived survival	

author	title	journal	OPN	CD44	cells	species	properties	effect	condition
Kato Y, Windle JJ, Koop BA, Mundy GR, Bonewald LF.	Establishment of an osteocyte-like cell line, MLO-Y4.	J Bone Miner Res. 1997 Dec;12(12):2014-23.	osteopontin expression	CD44 expression	osteocyte cell line	mouse			bone
Suzuki K, Zhu B, Rittling SR, Denhardt DT, Goldberg HA, McCulloch CA, Sodek J.	Colocalization of intracellular osteopontin with CD44 is associated with migration, cell fusion, and resorption in osteoclasts.	J Bone Miner Res. 2002 Aug;17(8):1486-97.	endogenous	endogenous	osteoblast	mouse	prominent in cell processes, including filopodia and pseudopodia	In OPN- and CD44- osteoclasts, reduced cell spreading and protrusion of pseudopodia and impaired cell fusion	bone
Thalmeier K, Meissner P, Moosmann S, Sagebiel S, Wiest I, Huss R.	Mesenchymal differentiation and organ distribution of established human stromal cell lines in NOD/SCID mice.	Acta Haematol. 2001;105(3):159-65.	variable expression	expression	osteoblastic stromal fibroblasts	homo sapiens		differentiation	bone
Rammelt S, Schulze E, Witt M, Petsch E, Biewener A, Pompe W, Zwipp H.	Collagen type I increases bone remodelling around hydroxyapatite implants in the rat tibia.	Cells Tissues Organs. 2004;178(3):146-57.	osteoblast-like cells express OPN at the interface 2 days after implantation	osteoblast-like cells express CD44 at the interface 2 days after implantation		rat		bone remodeling	bone
Chitteti BR, Cheng YH, Kacena MA, Srour EF.	Hierarchical organization of osteoblasts reveals the significant role of CD166 in hematopoietic stem cell maintenance and function.	Bone. 2013 May;54(1):58-67	osteoblast lineage marker	CD44 osteoblast lineage marker	LSK	mouse		hematopoietic stem cell maintenance and function	bone
Kusuma GD, Menicanin D, Gronthos S, Manuelpillai U, Abumaree MH, Pertile MD, Brennecke SP, Kalionis B.	Ectopic Bone Formation by Mesenchymal Stem Cells Derived from Human Term Placenta and the Decidua.	PLoS One. 2015 Oct 20;10(10):e0141246.	expression upon differentiation to bone	CD44 expression	mesenchymal stem cells	homo sapiens		ectopic bone formation	bone
Jäger M, Krauspe R.	Antigen expression of cord blood derived stem cells under osteogenic stimulation in vitro.	Cell Biol Int. 2007 Sep;31(9):950-7.	up under treatment with osteogenic mixture	up under treatment with osteogenic mixture	cord blood-derived stem cells	homo sapiens		differentiation	bone development
Henstock JR, Rotherham M, Rose JB, El Haj AJ.	Cyclic hydrostatic pressure stimulates enhanced bone development in the foetal chick femur in vitro.	Bone. 2013 Apr;53(2):468-77.	osteogenic gene OPN and type-II collagen in epiphyses and diaphysis upregulated	osteocyte maturation marker CD44 and type-II collagen in epiphyses and diaphysis upregulated	osteochondral cells and their progenitors	chicken	increased expression of OPN and CD44	bone formation	bone development, cyclic hydrostatic presure
Otsuru S, Tamai K, Yamazaki T, Yoshikawa H, Kaneda Y.	Circulating bone marrow-derived osteoblast progenitor cells are recruited to the bone-forming site by the CXCR4/stromal cell-derived factor-1 pathway.	Stem Cells. 2008 Jan;26(1):223-34.		bone marrow-derived osteoblast progenitor cells express CD44	bone marrow-derived osteoblast progenitor cells	mouse	CD44 and CXCR$ on bone marrow-derived osteoblast progenitor cells facilitate homing	ectopic bone formation	bone formation
Berbéri A, Al-Nemer F, Hamade E, Noujeim Z, Badran B, Zibara K.	Mesenchymal stem cells with osteogenic potential in human maxillary sinus membrane: an in vitro study.	Clin Oral Investig. 2017 Jun;21(5):1599-1609.	osteogenic differentiation upregulated osteogenic markers, including OPN	very high expression of mesenchymal progenitor cell markers such as CD44	osteogenic progenitor cell population	homo sapiens		differentiation	bone formation
Duan W, Lopez MJ.	Effects of Cryopreservation on Canine Multipotent Stromal Cells from Subcutaneous and Infrapatellar Adipose Tissue.	Stem Cell Rev. 2016 Apr;12(2):257-68.	expression increased in fresh and cryopreserved P3 adipose derived multipotent stromal cells	most fresh adipose-derived multipotent stromal cells are CD44+	multipotent stromal cells	dog		expansion, multipotentiality	bone, differentiation
Hu Y, Tang XX, He HY.	Gene expression during induced differentiation of sheep bone marrow mesenchymal stem cells into osteoblasts.	Genet Mol Res. 2013 Dec 11;12(4):6527-34.	stage-specifically expressed osteoblast marker	CD44 expressed in bone marrow mesenchymal stem cells	bone marrow mesenchymal stem cells	sheep		osteoinduction	bone, osteoblast differentiation
de Girolamo L, Sartori MF, Albisetti W, Brini AT.	Osteogenic differentiation of human adipose-derived stem cells: comparison of two different inductive media.	J Tissue Eng Regen Med. 2007 Mar-Apr;1(2):154-7.	expression during differentiation	expression during differentiation	adipose-derived stem cells	homo sapiens		differentiation	bone, osteogenic differentiation
Kim HK, Kim JH, Abbas AA, Kim DO, Park SJ, Chung JY, Song EK, Yoon TR.	Red light of 647 nm enhances osteogenic differentiation in mesenchymal stem cells.	Lasers Med Sci. 2009 Mar;24(2):214-22.	expressed during differentiation	expressed during differentiation	mesenchymal stem cells	mouse		differentiation	bone, osteogenic differentiation
Liu F, Akiyama Y, Tai S, Maruyama K, Kawaguchi Y, Muramatsu K, Yamaguchi K.	Changes in the expression of CD106, osteogenic genes, and transcription factors involved in the osteogenic differentiation of human bone marrow mesenchymal stem cells.	J Bone Miner Metab. 2008;26(4):312-20.	expressed during differentiation	expressed in stem cells	mesenchymal stem cells	homo sapiens		differentiation	bone, osteogenic differentiation
Maurice S, Srouji S, Livne E.	Isolation of progenitor cells from cord blood using adhesion matrices.	Cytotechnology. 2007 Jun;54(2):121-33.	expressed	expressed	mesenchymal progenitor cells	homo sapiens			bone, osteogenic differentiation
Kim HK, Park SJ, Kim JH, Kim J, Lee KY, Yoon TR.	Nanoemulsion-eicosapentaenoic acid enhanced alkaline phosphatase, calcium contents, and surface molecules expression during osteogenesis using mouse multipotent bone marrow stromal cells.	J Nanosci Nanotechnol. 2010 May;10(5):3284-8.	expression during differentiation	expression during differentiation	bone marrow stromal cells	mouse		osteogenesis	bone, osteogenic differentiation
Rada T, Reis RL, Gomes ME.	Distinct stem cells subpopulations isolated from human adipose tissue exhibit different chondrogenic and osteogenic differentiation potential.	Stem Cell Rev. 2011 Mar;7(1):64-76.	expression during differentiation	expression during differentiation	adipose derived stem cells	homo sapiens		differentiation	bone, osteogenic differentiation
Galindo-Moreno P, Hernández-Cortés P, Aneiros-Fernández J, Camara M, Mesa F, Wallace S, O'Valle F.	Morphological evidences of Bio-Oss® colonization by CD44-positive cells.	Clin Oral Implants Res. 2014 Mar;25(3):366-371.	expression on the interstitial boundary of bone with ABB particles, within osteocyte lacunae and bone canaliculi	expression correlated with number of blood vessels		ox		osteone formation, vascularization	bone healing
Liu H, Cui J, Sun J, Du J, Feng W, Sun B, Li J, Han X, Liu B, Yimin, Oda K, Amizuka N, Li M.	Histochemical evidence of zoledronate inhibiting c-src expression and interfering with CD44/OPN-mediated osteoclast adhesion in the tibiae of mice.	J Mol Histol. 2015 Jun;46(3):313-23.		endogenous	osteoclast, osteoblast	mouse	no expression of CD44 or OPN in osteoclasts of the zolendronate group	osteoclast adhesion	bone remodeling
Yamazaki M, Nakajima F, Ogasawara A, Moriya H, Majeska RJ, Einhorn TA.	Spatial and temporal distribution of CD44 and osteopontin in fracture callus.	J Bone Joint Surg Br. 1999 May;81(3):508-15.	OPN in remodelling areas of the hard callus; osteocytes, osteoclasts, osteoprogenitor cells, but not cuboidal osteoblasts	CD44 in remodelling callus; intensely in osteocyte lacunae, along canaliculi, on basolateral osteoclast membrane, not in cuboidal osteoblasts	bone	rat	OPN, not HA, may be the major CD44 ligand on bone cells during remodelling/fracture healing	skeletal tissue repair	bone, fracture

Rammelt S, Neumann M, Hanisch U, Reinstorf A, Pompe W, Zwipp H, Biewener A.	Osteocalcin enhances bone remodeling around hydroxyapatite/collagen composites.	J Biomed Mater Res A. 2005 Jun 1;73(3):284-94.	OPN at the implant interface enhanced by osteocalcin	CD44 at the implant interface enhanced by osteocalcin		rat	Osteocalcin activates osteoclasts and osteoblasts during early bone formation	bone remodeling	bone, remodeling
Liu H, Li D, Liu S, Liu Z, Li M.	Histochemical evidence of IGF2 mRNA-binding protein 2-mediated regulation of osteoclast function and adhesive ability.	Histochem Cell Biol. 2018 Apr;149(4):343-351.	in IMP2−/− mice, OPN was weakly positive in osteoclasts	faint immunoreactivity in IMP-/- mice		mouse	inhibited osteoclast adhesion in IMP2-deficient micce, owing to defects in the CD44-osteopontin signaling pathway	osteoclast adhesion	bone, remodeling
Luo T, Liu H, Feng W, Liu D, Du J, Sun J, Wang W, Han X, Guo J, Amizuka N, Li X, Li M.	Adipocytes enhance expression of osteoclast adhesion-related molecules through the CXCL12/CXCR4 signalling pathway.	Cell Prolif. 2017 Jun;50(3).			adipocytes, osteoclasts, ST-2, RAW264.7	mouse	CXCL12 caused increase in osteoclast adhesion molecules, including β3 integrin, CD44 and osteopontin		bone, resorption
Arslan YE, Sezgin Arslan T, Derkus B, Emregul E, Emregul KC.	Fabrication of human hair keratin/jellyfish collagen/eggshell-derived hydroxyapatite osteoinductive biocomposite scaffolds for bone tissue engineering: From waste to regenerative medicine products.	Colloids Surf B Biointerfaces. 2017 Jun 1;154:160-170.	expressed in osteogenic differentiation of adipose mesenchymal stem cells	undifferentiated adipose mesenchymal stem cells were highly positive for CD44		homo sapiens		tissue engineering	bone, tissue engineering
Nakamura H, Saruwatari L, Aita H, Takeuchi K, Ogawa T.	Molecular and biomechanical characterization of mineralized tissue by dental pulp cells on titanium.	J Dent Res. 2005 Jun;84(6):515-20.	up-regulated on titanium	dental pulp cells express CD44	dental pulp cells	rat		implant	bone, titanium implants
Duff MD, Mestre J, Maddali S, Yan ZP, Stapleton P, Daly JM.	Analysis of gene expression in the tumor-associated macrophage.	J Surg Res. 2007 Sep;142(1):119-28.	upregulation	upregulation	tumor-associated macrophage	mouse		presumed increase in inhibitory pathways	cancer
Fok TC, Lapointe H, Tuck AB, Chambers AF, Jackson-Boeters L, Daley TD, Darling MR.	Expression and localization of osteopontin, homing cell adhesion molecule/CD44, and integrin αvβ3 in pleomorphic adenoma, polymorphous low-grade adenocarcinoma, and adenoid cystic carcinoma.	Oral Surg Oral Med Oral Pathol Oral Radiol. 2013 Dec;116(6):743-51.	expression increased in low-grade adenocarcinoma and intermediate-/high-grade adenoid cyctic carcinoma	expression reduced in pleomorphic adenoma, polymorphous low-grade adenocarcinoma and adenoid cystic carcinoma		homo sapiens	OPN expression levels were moderately correlated with CD44 in PLGA; OPN was expressed in salivary gland tumors but does not correlate well with CD44 and αvβ3		cancer, adenoma
Fok TC, Lapointe H, Tuck AB, Chambers AF, Jackson-Boeters L, Daley TD, Darling MR.	Expression and localization of osteopontin, homing cell adhesion molecule/CD44, and integrin αvβ3 in mucoepidermoid carcinoma and acinic cell adenocarcinoma of salivary gland origin	Oral Surg Oral Med Oral Pathol Oral Radiol. 2014 Sep;118(3):320-9.	expression was increased in adenocarcinoma and mucoepidermoid carcinoma	CD44 expression was increased in adenocarcinoma, but reduced in mucoepidermoid carcinoma and pleomorphc adenoma		homo sapiens			cancer, salivary gland tumors
Wang YP, Liu BY.	Expression of osteopontin and its receptors in ameloblastomas.	Oral Oncol. 2009 Jun;45(6):538-42.	most cases positive	most cases positive for CD44v6		homo sapiens	high expression of OPN and CD44v6 in ameloblast-like and stellate reticulum-like cells		cancer, ameloblastoma
Qi S, Huang G, Pan J, Li J, Zhang X, Fang L, Liu B, Meng W, Zhang Y, Liu X.	Involvement of osteopontin as a core protein in craniopharyngioma calcification formation.	J Neurooncol. 2010 May;98(1):21-30.		expression of CD44v6		homo sapiens	OPN expression was paralleled by cell surface reactivity for CD44v6	calcification	cancer, craniopharyngioma
Miller CT, Lin L, Casper AM, Lim J, Thomas DG, Orringer MB, Chang AC, Chambers AF, Giordano TJ, Glover TW, Beer DG.	Genomic amplification of MET with boundaries within fragile site FRA7G and upregulation of MET pathways in esophageal adenocarcinoma.	Oncogene. 2006 Jan 19;25(3):409-18.	increased expression on days 7 and day 14 in TSP2 knockout cells	overexpression of CD44		homo sapiens	OPN is in the MET pathway and associated with invasive growth, CD44 is involved in MET autophosphorylation upon HGF stimulation		cancer, esophageal adenocarcinoma
Ue T, Yokozaki H, Kitadai Y, Yamamoto S, Yasui W, Ishikawa T, Tahara E.	Co-expression of osteopontin and CD44v9 in gastric cancer.	Int J Cancer. 1998 Apr 17;79(2):127-32.	OPN RNA upregulated over gastric mucosa	CD44v9 high		homo sapiens	positivity for OPN and CD44v9 correlated with lymphatic vessel invasion/long distant lymph node metastases	invasion/metastasis	cancer, gastric
Hsu KH, Tsai HW, Lin PW, Hsu YS, Shan YS, Lu PJ.	Osteopontin expression is an independent adverse prognostic factor in resectable gastrointestinal stromal tumor and its interaction with CD44 promotes tumor proliferation.	Ann Surg Oncol. 2010 Nov;17(11):3043-52.	increased expression is poor prognostic factor for recurrence and disease-free survival			homo sapiens	association between OPN and CD44	progression	cancer, gastrointestinal stromal tumor
Xu H, Posner GH, Stevenson M, Campbell FC.	Apc(MIN) modulation of vitamin D secosteroid growth control.	Carcinogenesis. 2010 Aug;31(8):1434-41.	expression levels increased in APC-, enhanced by vitamin D	expression levels increased in APC-, decreased by vitamin D		mouse	vitamin D enhanced OPN but inhibited expression of CD44	differential response to vitamin D	cancer, colon
Seo KJ, Kim M, Kim J.	Prognostic implications of adhesion molecule expression in colorectal cancer.	Int J Clin Exp Pathol. 2015 Apr 1;8(4):4148-57.	expression associated with poor tumor differentiation	CD44 expression associated with poor tumor differentiation		homo sapiens		prognosis	cancer, colorectal
Nakamura H, Hiraga T, Ninomiya T, Hosoya A, Fujisaki N, Yoneda T, Ozawa H.	Involvement of cell-cell and cell-matrix interactions in bone destruction induced by metastatic MDA-MB-231 human breast cancer cells in nude mice.	J Bone Miner Metab. 2008;26(6):642-7.		breast cancer cells and osteoclast-like cells positive	MDA-MB-231	homo sapiens	Hyaluronan and OPN were occasionally colocalized with CD44.	bone metastasis	cancer, breast
Ling LJ, Wang S, Liu XA, Shen EC, Ding Q, Lu C, Xu J, Cao QH, Zhu HQ, Wang F.	A novel mouse model of human breast cancer stem-like cells with high CD44+CD24-/lower phenotype metastasis to human bone.	Chin Med J (Engl). 2008 Oct 20;121(20):1980-6.	strong expression	strong expression	cancer stem cells	mouse		bone metastasis	cancer, breast
Gao YL, Xing LQ, Ren TJ, Hou JF, Xue Q, Liu C, Han YM.	The expression of osteopontin in breast cancer tissue and its relationship with p21ras and CD44V6 expression.	Eur J Gynaecol Oncol. 2016;37(1):41-7.	expression of OPN and p21ras were positively correlated			homo sapiens	expression of OPN and CD44v6 were positively correlated	prognostic markers	cancer, breast
Mo B, Vendrov AE, Palomino WA, DuPont BR, Apparao KB, Lessey BA.	ECC-1 cells: a well-differentiated steroid-responsive endometrial cell line with characteristics of luminal epithelium.	Biol Reprod. 2006 Sep;75(3):387-94.	expressed	expressed	ECC-1, well-differentiated, steroid-responsive endometrial cell	homo sapiens			cancer, endometrial
Hira VVV, Wormer JR, Kakar H, Breznik B, van der Swaan B, Hulsbos R, Tigchelaar W, Tonar Z, Khurshed M, Molenaar RJ, Van Noorden CJF.	Periarteriolar Glioblastoma Stem Cell Niches Express Bone Marrow Hematopoietic Stem Cell Niche Proteins.	J Histochem Cytochem. 2018 Mar;66(3):155-173.	endogenous expression	endogenous expression	glioblastoma stem cell niche	homo sapiens	hematopoietic stem cell niches are retained by binding of SDF-1α and OPN to their receptors CXCR4 and CD44		cancer, glioblastoma

Gao YF, Li X, Xie QX, Gui SY, Wang Y, Zhou Q, Li JB, Jiang XP.	[Expressions of osteopontin and CD44v6 in hepatocellular carcinoma and their clinical significance].	Zhonghua Gan Zang Bing Za Zhi. 2005 Mar;13(3):227-8.				homo sapiens			cancer, hepatocellular carcinoma
Yang GH, Fan J, Xu Y, Qiu SJ, Yang XR, Shi GM, Wu B, Dai Z, Liu YK, Tang ZY, Zhou J.	Osteopontin combined with CD44, a novel prognostic biomarker for patients with hepatocellular carcinoma undergoing curative resection.	Oncologist. 2008 Nov;13(11):1155-65.	predictor for overall survival and disease-free survival	predictor for overall survival and disease-free survival		homo sapiens	extended predictive range and improved sensitivity by considering OPN and CD44		cancer, hepatocellular carcinoma
Beckebaum S, Chen X, Sotiropoulos GC, Radtke A, Daoudaki M, Baba HA, Wohlschlaeger J, Broelsch CE, Gerken G, Cicinnati VR.	Role of osteopontin and CD44s expression for patients with hepatocellular carcinoma undergoing liver transplantation or resection.	Transplant Proc. 2008 Nov;40(9):3182-4.	elevated expression in tumor; overexpression associated with high grade	elevated expression of CD44s in tumor		homo sapiens	correlation between OPN and CD44s expression		cancer, hepatocellular carcinoma
Philips GM, Chan IS, Swiderska M, Schroder VT, Guy C, Karaca GF, Moylan C, Venkatraman T, Feuerlein S, Syn WK, Jung Y, Witek RP, Choi S, Michelotti GA, Rangwala F, Merkle E, Lascola C, Diehl AM.	Hedgehog signaling antagonist promotes regression of both liver fibrosis and hepatocellular carcinoma in a murine model of primary liver cancer.	PLoS One. 2011;6(9):e23943.		expressed on progenitor cells		mouse	stem/progenitor cell populations for many types of cancer, including HCC, are thought to be enriched with cells that express CD44, a receptor for the stem cell growth factor, OPN	inhibition of the Hh pathway decreased OPN and CD44 expresion in tumor cells	cancer, hepatocellular carcinoma
Gao X, Sheng Y, Yang J, Wang C, Zhang R, Zhu Y, Zhang Z, Zhang K, Yan S, Sun H, Wei J, Wang X, Yu X, Zhang Y, Luo Q, Zheng Y, Qiao P, Zhao Y, Dong Q, Qin L.	Osteopontin alters DNA methylation through up-regulating DNMT1 and sensitizes CD133+/CD44+ cancer stem cells to 5 azacytidine in hepatocellular carcinoma.	J Exp Clin Cancer Res. 2018 Jul 31;37(1):179.	expression alters DNA methylation through up-regulating DNMT1	marker for stemness together with CD133		homo sapiens	OPN expression correlated with CD44 expression (TCGA)		cancer, hepatocellular carcinoma
Augustin F, Fiegl M, Schmid T, Pomme G, Sterlacci W, Tzankov A.	Receptor for hyaluronic acid-mediated motility (RHAMM, CD168) expression is prognostically important in both nodal negative and nodal positive large cell lung cancer.	J Clin Pathol. 2015 May;68(5):368-73.				homo sapiens		survival	cancer, large cell lung
Celetti A, Testa D, Staibano S, Merolla F, Guarino V, Castellone MD, Iovine R, Mansueto G, Somma P, De Rosa G, Galli V, Melillo RM, Santoro M.	Overexpression of the cytokine osteopontin identifies aggressive laryngeal squamous cell carcinomas and enhances carcinoma cell proliferation and invasiveness.	Clin Cancer Res. 2005 Nov 15;11(22):8019-27.	expression elevated in all invasive carcinomas	highly expressed	squamous carcinoma cells	homo sapiens	OPN expression was paralleled by intense cell surface reactivity for CD44v6	increased proliferation and matrigel invasion	cancer, laryngeal squamous cell carcinoma
Yang L, Shang X, Zhao X, Lin Y, Liu J.	[Correlation study between OPN, CD44v6, MMP-9 and distant metastasis in laryngeal squamous cell carcinoma].	Lin Chung Er Bi Yan Hou Tou Jing Wai Ke Za Zhi. 2012		CD44v6		homo sapiens	high level of expression of OPN, CD44v6 and MMP-9	metastasis	cancer, laryngeal squamous cell carcinoma
Shin S, Wangensteen KJ, Teta-Bissett M, Wang YJ, Mosleh-Shirazi E, Buza EL, Greenbaum LE, Kaestner KH.	Genetic lineage tracing analysis of the cell of origin of hepatotoxin-induced liver tumors in mice.	Hepatology. 2016 Oct;64(4):1163-1177.	expression positive	expression positive	hepatocytes	mouse		carcinogenesis	cancer, liver
Li M, Amizuka N, Takeuchi K, Freitas PH, Kawano Y, Hoshino M, Oda K, Nozawa-Inoue K, Maeda T.	Histochemical evidence of osteoclastic degradation of extracellular matrix in osteolytic metastasis originating from human lung small carcinoma (SBC-5) cells.	Microsc Res Tech. 2006 Feb;69(2):73-83	stromal tissues were markedly positive	osteoclasts expressed immunoreactivity for CD44		homo sapiens		osteoclast migration, degradation of unmineralized extracellular matrix	cancer, lung
Yang Y, Cheng D, Li X, Fang X.	[Expression and clinicopathological significance of OPN and CD44v6 in lung cancer].	Zhongguo Fei Ai Za Zhi. 2007 Apr 20;10(2):98-101.	expressed in NSCLC, but not in small cell lung cnacer	expressed in NSCLC, but not in small cell lung cancer		homo sapiens	positive correlation between the expression of OPN and CD44v6	expression closely related to TNM stage	cancer, lung
Sun BS, Li Y, Zhang ZF, You J, Wang CL.	Osteopontin combined with CD44v6, a novel prognostic biomarker in non-small cell lung cancer undergoing curative resection.	Ann Thorac Surg. 2013 Dec;96(6):1943-51.	independent predictor for overall survival and disease-free survival	CD44v6 independent predictor for overall survival and disease-free survival		homo sapiens	exteded predictive range and improved sensitivity by OPN and CD44v6 together		cancer, lung
Yu J, Pan T, Li J, Wei X, Chen T, Hu M, Song D, Liu L, Chen P.	[Expression and clinicopathologic significance of OPN, CD44v6 and MMP-2 in squamous cell carcinoma and adenocarcinoma of the lung].	Zhongguo Fei Ai Za Zhi. 2006;9(4):325-8. doi: 10.3779/j.	related to histological classification	related to histological classification		homo sapiens	positive correlation between OPN and CD44v6 expression		cancer, lung carcinoma
Le QT, Chen E, Salim A, Cao H, Kong CS, Whyte R, Donington J, Cannon W, Wakelee H, Tibshirani R, Mitchell JD, Richardson D, O'Byrne KJ, Koong AC, Giaccia AJ.	An evaluation of tumor oxygenation and gene expression in patients with early stage non-small cell lung cancers.	Clin Cancer Res. 2006 Mar 1;12(5):1507-14.	correlation between tumor/lung pO2 with plasma OPN and CA IX expression	expression is a predictor for relapse, confirmed by tissue staining of CD44v6		homo sapiens	tumor hypoxia, elevated osteopontin levels, CD44 expression correlated with poor prognosis		cancer, non-small cell lung
Higashi A, Dohi Y, Uraoka N, Sentani K, Uga S, Kinoshita H, Sada Y, Kitagawa T, Hidaka T, Kurisu S, Yamamoto H, Yasui W, Kihara Y.	The Potential Role of Inflammation Associated with Interaction between Osteopontin and CD44 in a Case of Pulmonary Tumor Thrombotic Microangiopathy Caused by Breast Cancer.	Intern Med. 2015;54(22):2877-80.	expression of OPN in macrophages that had migrated into the PTTM lesions	expression of CD44 in macrophages that had migrated into the PTTM lesions	macrrophage	homo sapiens		microangiopathy	cancer, lung, pulmonary tumor thrombotic microangiopathy
Liu D, Li Y, Wang X, Wang Y, Ma Y, Chen Y, Ming H.	[Astragalus polysaccharide combined with cisplatin inhibits growth of recurrent tumor and down-regulats the expression of CD44, CD62P and osteopontin in tumor tissues in mice bearing Lewis lung cancer].	Xi Bao Yu Fen Zi Mian Yi Xue Za Zhi. 2018 Dec;34(12):1105-1110.	decreased expression	decreased expression	Lewis lung carcinoma	mouse		cancer treatment with Astragalus polysaccharide plus cisplatin	cancer, lung, treatment
Li HZ, Gong HD, Wang C, Li JK.	The role of osteopontin and its receptor in meningioma development and progression.	J Biol Regul Homeost Agents. 2018 Jan-Feb;32(1):69-74.	expression negative in normal meninx, increased in meningioma according to grade	CD44 expression correlated with grade and invasiveness, no correlation with the degree of peritumoral edema		homo sapiens	increased expressions of OPN and CD44 in all grades of meningioma, both were correlated with its development	progression	cancer, meningioma
Bachmann IM, Ladstein RG, Straume O, Naumov GN, Akslen LA.	Tumor necrosis is associated with increased alphavbeta3 integrin expression and poor prognosis in nodular cutaneous melanomas.	BMC Cancer. 2008 Dec 5;8:362.	expression correlated with increased tumor cell proliferation; much increased in metastatic lesions	Apaf-1, a marker of the mitochondrial apoptotic pathway, strongly associated with increased expression of CD44		homo sapiens	OPN expression was associated with β3-integrin; CD44 was co-expressed with αv-integrin		cancer, nodular cutaneous melanoma

Galic Jerman K, Kobal B, Jakimovska M, Verdenik I, Cerne K.	Control values of ovarian cancer tumor markers and standardisation of a protocol for sampling peritoneal fluid and performing washing during laparoscopy.	World J Surg Oncol. 2014 Sep 4;12:278.		sCD44v6	peritoneal lavage	homo sapiens	concentrations of sOPN, sCD44-v6 and sVCAM-1 in peritoneal fluid correlate with peritoneal washing	abundance of tumor markers	cancer, ovarian
Gaviraghi M, Tunici P, Valensin S, Rossi M, Giordano C, Magnoni L, Dandrea M, Montagna L, Ritelli R, Scarpa A, Bakker A.	Pancreatic cancer spheres are more than just aggregates of stem marker-positive cells.	Biosci Rep. 2011 Feb;31(1):45-55.	marker of aggressiveness	CD44v6 marker of aggressiveness	PANC-1, CFPAC-1, PancTu-1, PSN-1	homo sapiens		sphere formation	cancer, pancreatic
Matusan K, Dordevic G, Mozetic V, Lucin K.	Expression of osteopontin and CD44 molecule in papillary renal cell tumors.	Pathol Oncol Res. 2005;11(2):108-13.	present distal tubular epithelial cells, expressed in all adenomas and all papillary renal cell carcinomas	normal renal tissue was negative for CD44s and v6, both forms were upregulated in carcinomas		homo sapiens	CD44s correlated with lower stage		cancer, papillary renal cell cancer
Guarino V, Faviana P, Salvatore G, Castellone MD, Cirafici AM, De Falco V, Celetti A, Giannini R, Basolo F, Melillo RM, Santoro M.	Osteopontin is overexpressed in human papillary thyroid carcinomas and enhances thyroid carcinoma cell invasiveness.	J Clin Endocrinol Metab. 2005 Sep;90(9):5270-8.	overexpressed in most papillary thyroid carcinomas	overexpressed in most papillary thyroid carcinomas		homo sapiens		OPN correlated with lymph node metastases and tumor size	cancer, thyroid carcinoma
Ferrari E, Wittig A, Basilico F, Rossi R, De Palma A, Di Silvestre D, Sauerwein WAG, Mauri PL.	Urinary Proteomics Profiles Are Useful for Detection of Cancer Biomarkers and Changes Induced by Therapeutic Procedures.	Molecules. 2019 Feb 22;24(4). pii: E794.	redued in urinary proteome	redued in urinary proteome		homo sapeins	boron neutron capture therapy of head and neck squamous cell carcinoma, thyroid cancer	Galectin-3 Binding Protein also reduced	cancer, thyroid + head and neck
Gaumann A, Petrow P, Mentzel T, Mayer E, Dahm M, Otto M, Kirkpatrick CJ, Kriegsmann J.	Osteopontin expression in primary sarcomas of the pulmonary artery.	Virchows Arch. 2001 Nov;439(5):668-74.	strong staining in tumor cells and the adjacent extracellular matrix	expressed on the cellular surface of tumor-associated lymphocytes		homo sapiens	OPN from macrophages and tumor cells may interact with CD44 on lymphocytes		cancer, sarcoma
Elli M, Dagdemir A, Bozkurt C, Pinarli FG, Duzgun A, Ozmen ZC, Ertem U, Acar S.	Serum osteopontin and CD44 levels in lymphoreticular malignancies in children.	Bratisl Lek Listy. 2012;113(9):534-8.	higher in patients than in controls	higher in patients than in controls, higher with advanced stges		homo sapiens	OPN and CD44 levels were increased in parallel		cancer, pediatric lymphoretic ular malignancy
Al-Asadi MG, Brindle G, Castellanos M, May ST, Mills KI, Russell NH, Seedhouse CH, Pallis M.	A molecular signature of dormancy in CD34+CD38- acute myeloid leukaemia cells.	Oncotarget. 2017 Nov 30;8(67):111405-111418.	most upregulated gene in dormant leukemia cells	upregulation of CD44	CD34+CD38- AML cells	homo sapiens		dormancy	cancer, acute myeloid leukemia
Nagel S, Hirschmann P, Dirnhofer S, Günthert U, Tzankov A.	Coexpression of CD44 variant isoforms and receptor for hyaluronic acid-mediated motility (RHAMM, CD168) is an International Prognostic Index and C-MYC gene status-independent predictor of poor outcome in diffuse large B-cell lymphomas.	Exp Hematol. 2010 Jan;38(1):38-45.	endogenous	endogenous		homo sapiens	CD44v-RHAMM coexpression was most prevalent in non-germinal center DLBCL cases and coincided with OPN expression		cancer, diffuse large B-cell lymphoma
Atkins K, Berry JE, Zhang WZ, Harris JF, Chambers AF, Simpson RU, Somerman MJ.	Coordinate expression of OPN and associated receptors during monocyte/macrophage differentiation of HL-60 cells.	J Cell Physiol. 1998 May;175(2):229-37.	upregulated by PMA but not by retinoic acid	cells express CD44, PMA (but not retinic acid) upregulates CD44	HL-60	homo sapiens	cells expressed integrin β1; PMA induced αvβ3 and increased β1 expression	adhesion following monocytic differentiation	cancer, promyelocy tic leukemia
Pacheco-Rodriguez G, Steagall WK, Crooks DM, Stevens LA, Hashimoto H, Li S, Wang JA, Darling TN, Moss J.	TSC2 loss in lymphangioleiomyomatosis cells correlated with expression of CD44v6, a molecular determinant of metastasis.		increased plasma concentration	CD44v6 present in lung nodules, but not in normal vascular smooth muscle cells	smooth muscle-like cells, Eker rat cells	rat, human	CD44v6+ cells showed loss of heterozygosity at the TSC2 locus	dissemination	lymphangiol eiomyomat osis
Infanger M, Kossmehl P, Shakibaei M, Bauer J, Kossmehl-Zorn S, Cogoli A, Curcio F, Oksche A, Wehland M, Kreutz R, Paul M, Grimm D.	Simulated weightlessness changes the cytoskeleton and extracellular matrix proteins in papillary thyroid carcinoma cells.	Cell Tissue Res. 2006 May;324(2):267-77.	increasingly expressed after 48 hours	increasingly expressed after 48 hours	thyroid cancer cells, ONCO-DG 1	homo sapiens	weightlessness affects the cytoskeleton of papillary thyroid carcinoma cells	recovery of multicellular spheroids	microgravit y/cancer, thyroid
Grosse J, Wehland M, Pietsch J, Schulz H, Saar K, Hübner N, Eilles C, Bauer J, Abou-El-Ardat K, Baatout S, Ma X, Infanger M, Hemmersbach R, Grimm D.	Gravity-sensitive signaling drives 3-dimensional formation of multicellular thyroid cancer spheroids.	FASEB J. 2012 Dec;26(12):5124-40.	up-regulated in adherent cells but not in multicellular tumor spheroids in microgravity	CD44 up-regulated in adherent cells but not in multicellular tumor spheroids in microgravity	FTC-133 thyroid cancer cells	homo sapiens		spheroid formation	microgravit y/cancer, thyroid
Ventorp F, Barzilay R, Erhardt S, Samuelsson M, Träskman-Bendz L, Janelidze S, Weizman A, Offen D, Brundin L.	The CD44 ligand hyaluronic acid is elevated in the cerebrospinal fluid of suicide attempters and is associated with increased blood-brain barrier permeability.	J Affect Disord. 2016 Mar 15;193:349-54.		decreased cerebrospinal CD44 levels in depressed individuals, CD44 is possible risk gene for suicidal behavior		homo sapiens	correlation between age and sCD44, HA and OPN	no differences in CSF levels of MMP3, MMP1, OPN between suicide attempters and controls	suicide
Kang WS, Choi JS, Shin YJ, Kim HY, Cha JH, Lee JY, Chun MH, Lee MY.	Differential regulation of osteopontin receptors, CD44 and the alpha(v) and beta(3) integrin subunits, in the rat hippocampus following transient forebrain ischemia.	Brain Res. 2008 Sep 4;1228:208-16.	rapid and transient induction of CD44 and OPN in activated microglia/macrophages	induced at day 1 after reperfusion, reached a peak at day 3, and returned to basal levels by day 7		rat	OPN and CD44 induction in a subset of activated microglia at sites of intense neural damage (ischemic hippocampus)		CNS, forebrain ischemia
Marcondes MC, Ojakian R, Bortell N, Flynn C, Conti B, Fox HS.	Osteopontin expression in the brain triggers localized inflammation and cell death when immune cells are activated by pertussis toxin.	Mediators Inflamm. 2014;2014:358218.		CD44v6 induced by pertussis toxin		mouse	inflammatory infiltrate induced by OPN plus peripheral treatment with pertussis toxin, which activated peripheral cells to express CD44v6	cell death	CNS, inflammation
Ailane S, Long P, Jenner P, Rose S.	Expression of integrin and CD44 receptors recognising osteopontin in the normal and LPS-lesioned rat substantia nigra.	Eur J Neurosci. 2013 Aug;38(3):2468-76.	OPN interacted with integrin and CD44	upregulated expression in LPS-lesioned substantia nigra		rat	up-regulation of integrin β3 and CD44 in lesions	neurodegeneration	CNS, Parkinson's disease
Hasegawa-Ishii S, Takei S, Inaba M, Umegaki H, Chiba Y, Furukawa A, Kawamura N, Hosokawa M, Shimada A.	Defects in cytokine-mediated neuroprotective glial responses to excitotoxic hippocampal injury in senescence-accelerated mouse.	Brain Behav Immun. 2011 Jan;25(1):83-100.	the most strongly upregulated cytokine	CD44 was strongly upregulated in the neuropil, especially on neurons and astrocytes		mouse	the OPN-CD44 system is essential for neuroprotection and remodeling	well-orchestrated cytokine-mediated glial interactions in the injured hippocampus	CNS, senescence
Borges K, Gearing M, Rittling S, Sorensen ES, Kotloski R, Denhardt DT, Dingledine R.	Characterization of osteopontin expression and function after status epilepticus.	Epilepsia. 2008 Oct;49(10):1675-85.	immunoreactivity in neurons undergoing degeneration (2-3 days), degenerating axons in the thalamus (10 to 31 days)	in the supragranular layer above the dentate granule cell layer and the extracellular matrix; upregulation on the RNA level		mouse		no regulation of neurodegeneration and inflammation	CNS, status epilepticus

Powell MA, Black RT, Smith TL, Reeves TM, Phillips L Ph.D.	Matrix metalloproteinase 9 and osteopontin interact to support synaptogenesis in the olfactory bulb following mild traumatic brain injury.	J Neurotrauma. 2018 Nov 16.	bimodal induction, 1 and 7 days post injury, proteolytic fragments	increase peaked at 3 days		mouse	CD44 upregulation delayed relative to MMP9/OPN response	wound healing	CNS, brain injury
Sugiyama Y, Oishi T, Yamashita A, Murata Y, Yamamoto T, Takashima I, Isa T, Higo N.	Neuronal and microglial localization of secreted phosphoprotein 1 (osteopontin) in intact and damaged motor cortex of macaques.	Brain Res. 2019 Jul 1;1714:52-64.	immunoreactivity in the microglia colocalized with CD44	immunoreactivity in the microglia colocalized with SPP1	motor cortex	macaque	M1 motor cortex lesion	SPP1 immunoreactivity in perilesional neurons correlated with success in small-object-retrieval task	CNS, brain injury
Almolda B, Villacampa N, Manders P, Hidalgo J, Campbell IL, González B, Castellano B.	Effects of astrocyte-targeted production of interleukin-6 in the mouse on the host response to nerve injury.	Glia. 2014 Jul;62(7):1142-61.	alterations in osteopontin expression in motor neurons	alterations in CD44 in lymphocytes and microglia		mouse	microglial activation alters expression levels of OPN, CD44, CD49e	glial reactivity, lymphocyte infiltration	nerve injury
Marfia G, Navone SE, Di Vito C, Tabano S, Giammattei L, Di Cristofori A, Gualtierotti R, Tremolada C, Zavanone M, Caroli M, Torchia F, Miozzo M, Rampini P, Riboni L, Campanella R.	Gene expression profile analysis of human mesenchymal stem cells from herniated and degenerated intervertebral discs reveals different expression of osteopontin.	Stem Cells Dev. 2015 Feb 1;24(3):320-8.	markedly increased (more than 400-fold) in MSCs from degenerated compared to herniated intervertebral disk	higher expression of CD44 in degenerated compared to herniated intervertebral discs	mesenchymal stem cells	homo sapiens		disc degeneration	herniated and degenerated intervertebral discs
Murugaiyan G, Mittal A, Weiner HL.	Increased osteopontin expression in dendritic cells amplifies IL-17 production by CD4+ T cells in experimental autoimmune encephalomyelitis and in multiple sclerosis.	J Immunol. 2008 Dec 1;181(11):7480-8.	in subjects with MS, increased expression of OPN in dendritic cells	increased expression of CD44 on T cells		homo sapiens		inhibition of IL-10 secretion	autoimmunity
Kim MD, Cho HJ, Shin T.	Expression of osteopontin and its ligand, CD44, in the spinal cords of Lewis rats with experimental autoimmune encephalomyelitis.	J Neuroimmunol. 2004 Jun;151(1-2):78-84.	(constitutively expresed in astrocytes) significantly increased at the early and peak stage and slightly declined thereafter	(constitutively expressed in astrocytes) expressed in inflammatory cells, as well as increased expression in astrocytes in EAE	spinal cord	rat	OPN may interact with CD44 on astrocytes and inflammatory cells in autoimmune encephalomyelitis		autoimmunity, encephalomyelitis
Moon C, Shin T.	Increased expression of osteopontin in the spinal cords of Lewis rats with experimental autoimmune neuritis.	J Vet Sci. 2004 Dec;5(4):289-93.	increased in some astrocytes adjacent to the pia mater and neurons, present in some inflammatory cells (including macrophages) in the subarachnoid space	weak in normal spinal cord tissue, increased in the entire spinal cord parenchyma in autoimmune neuritis, as well as in inflammatory cells		rat	inflammatory cells and reactive astrocytes are major sources of OPN and CD44 in the spinal cord		autoimmunity, experimental autoimmune neuritis
Tahir S, Fukushima Y, Sakamoto K, Sato K, Fujita H, Inoue J, Uede T, Hamazaki Y, Hattori M, Minato N.	A CD153+CD4+ T follicular cell population with cell-senescence features plays a crucial role in lupus pathogenesis via osteopontin production.	J Immunol. 2015 Jun 15;194(12):5725-35.	CD153+ cells (PD1+CD44hiCD4+) preferentially secreting abundant OPN on TCR stimulation	PD-1+ CD44highCD4+ T cell population increases with age		mouse		autoimmunity	autoimmunity, lupus erythematosus
Yoshida T, Hanawa H, Toba K, Watanabe H, Watanabe R, Yoshida K, Abe S, Kato K, Kodama M, Aizawa Y.	Expression of immunological molecules by cardiomyocytes and inflammatory and interstitial cells in rat autoimmune myocarditis.	Cardiovasc Res. 2005 Nov 1;68(2):278-88.	expressed in CD11b+ cell and non-cardiomyocytic non-inflammatory cell fractions	mainly in a αβ-T cell fraction, smaller amounts in CD11b+ cells and non-cardiomyocytic non-inflammatory cell fractions		rat		inflammation	autoimmunity, myocarditis
Takada H, Nakazawa T, Ohara H, Ando T, Hayashi K, Naito I, Okumura F, Tanaka H, Yamada T, Takahashi S, Joh T.	Role of osteopontin in calcification in autoimmune pancreatitis.	Dig Dis Sci. 2009 Apr;54(4):793-801.	elevated expression in centroacinar cells in chronic pancreatitis with calcification and in autoimmune pancreatitis	some cases of chronic pancreatitis and autoimmune pancreatitis expressed CD44 in centroacinar cells and ductal cells		rat, human			autoimmunity, pancreatitis
Xu G, Nie H, Li N, Zheng W, Zhang D, Feng G, Ni L, Xu R, Hong J, Zhang JZ.	Role of osteopontin in amplification and perpetuation of rheumatoid synovitis.	J Clin Invest. 2005 Apr;115(4):1060-7.	OPN induction by IL-10, overexpression in synovial CD4+ T cells, elevation in synovial fluid	overexpression in synovial T cells	T-lymphocytes	homo sapiens	OPN overexpression pattern was confined to rheumatoid synovium, correlated with OPN receptors integrin αV, β1, CD44 in synovial T cells	amplification and perpetuation of rheumatoid synovitis	autoimmunity, rheumatoid arthritis
Khajoee V, Saito M, Takada H, Nomura A, Kusuhara K, Yoshida SI, Yoshikai Y, Hara T.	Novel roles of osteopontin and CXC chemokine ligand 7 in the defence against mycobacterial infection.	Clin Exp Immunol. 2006 Feb;143(2):260-8.	highly expressed after stimulation with BCG	expressed on induced macrophages	macrophage	homo sapiens		response to infection	infection
Martchenko M, Jeong SY, Cohen SN.	Heterodimeric integrin complexes containing beta1-integrin promote internalization and lethality of anthrax toxin.	Proc Natl Acad Sci U S A. 2010 Aug 31;107(35):15583-8.			macrophage, RAW264.7	mouse	toxin effects are asociated with the integrin-activating proteins OPN and CD44	integrin β1 can mediate and potentiate anthrax toxin endocytosis	infection, anthrax toxicity
Zhang J, Yamada O, Kida S, Matsushita Y, Yamaoka S, Chagan-Yasutan H, Hattori T.	Identification of CD44 as a downstream target of noncanonical NF-κB pathway activated by human T-cell leukemia virus type 1-encoded Tax protein.	Virology. 2011 May 10;413(2):244-52.	Human T-cell leukemia virus type 1 Tax induces OPN expression by transactivating its promoter	Tax, but not its NF-κB-defective mutant, increased CD44 promoter activity	Huh-7, HeLa cells	homo sapiens		T-cell leukemia virus type 1 Tax induces OPN and CD44 expression	infection, HTLV
Shiratori B, Zhao J, Okumura M, Chagan-Yasutan H, Yanai H, Mizuno K, Yoshiyama T, Idei T, Ashino Y, Nakajima C, Suzuki Y, Hattori T.	Immunological Roles of Elevated Plasma Levels of Matricellular Proteins in Japanese Patients with Pulmonary Tuberculosis.	Int J Mol Sci. 2016 Dec 22;18(1). pii: E19.	elevated in plasma	sCD44 elevated		homo sapiens	positive correlation between OPN and sCD44	response to infection	infection, tuberculosis
Zhu B, Suzuki K, Goldberg HA, Rittling SR, Denhardt DT, McCulloch CA, Sodek J.	Osteopontin modulates CD44-dependent chemotaxis of peritoneal macrophages through G-protein-coupled receptors: evidence of a role for an intracellular form of osteopontin.	J Cell Physiol. 2004 Jan;198(1):155-67.	OPN co-distributed with CD44 inside macrophages at cell edges and in cell processes in a mutually dependent manner	reduced cell-surface expression of CD44 in OPN-/- cells	peritoneal macrophages	mouse	fewer processes in OPN- and CD44- macrophages, reduced cell fusion, impaired chemotaxis; requirement for CD44-associated intracellular OPN in CD44 cell-surface expression	chemotaxis	inflammation
Arafat HA, Wein AJ, Chacko S.	Osteopontin gene expression and immunolocalization in the rabbit urinary tract.	J Urol. 2002 Feb;167(2 Pt 1):746-52.	expressed in adult renal pelvis, ureter, bladder and urethra, and neonatal bladder		urinary tract	rabbit	OPN expression in all layers of transitional bladder epithelium, co-localization with αvβ3-integrin mainly in superficial layers and with CD44 mainly in basal layers		kidney
Freese A, Wehland M, Freese F, Bamberg C, Kreutz R, Rothermund L.	Genetic low nephron number hypertension is associated with altered expression of osteopontin and CD44 during nephrogenesis.	J Perinat Med. 2013 May;41(3):295-9.	postpartum increased expression (fetal: decreased RNA and protein)	postpartum and fetal: renal RNA expression increased		rat		nephron number	kidney, low nephron number hypertension

Lan HY, Yu XQ, Yang N, Nikolic-Paterson DJ, Mu W, Pichler R, Johnson RJ, Atkins RC.	De novo glomerular osteopontin expression in rat crescentic glomerulonephritis.	Kidney Int. 1998 Jan;53(1):136-45.	expression in glomerular visceral and parietal epithelial cells (some expression in healthy kidney); expression in macrophage multinucleated giant cells	co-expression with osteopontin in intrinsic renal cells	parietal epithelial cells	rat	OPN expression preceded and correlate with macrophage infiltration; tubular OPN may promote interstitial macrophage infiltration (macrophages and T-cells were CD44-positive)	macrophage infiltration	kidney, crescentic glomerulonephritis
Xie Y, Nishi S, Iguchi S, Imai N, Sakatsume M, Saito A, Ikegame M, Iino N, Shimada H, Ueno M, Kawashima H, Arakawa M, Gejyo F.	Expression of osteopontin in gentamicin-induced acute tubular necrosis and its recovery process.	Kidney Int. 2001 Mar;59(3):959-74.	upregulated in PCNA positive cells	expression upregulated in the renal cortical tubular epithelium		rat		proliferation and regeneration of tubular epithelial cells	kidney, gentamicin-induced acute tubular necrosis
Nakamura H, Kitazawa K, Honda H, Sugisaki T.	Roles of and correlation between alpha-smooth muscle actin, CD44, hyaluronic acid and osteopontin in crescent formation in human glomerulonephritis.	Clin Nephrol. 2005 Dec;64(6):401-11.	significant expression of OPN in the cellular crescents compared with fibrocellular and fibrous crescents	significant expression of CD44 in the cellular crescents compared with fibrocellular and fibrous crescents		homo sapiens	CD44 expression in cellular crescents correlated with OPN and CD68, and with HA deposition	myofibroblasts and cell-matrix interactions mediated by CD44-OPN and CD44-HA may play roles in crescent formation and progression	kidney, glomerulonephritis
Okamoto T, Sasaki S, Yamazaki T, Sato Y, Ito H, Ariga T.	Prevalence of CD44-positive glomerular parietal epithelial cells reflects podocyte injury in adriamycin nephropathy.	Nephron Exp Nephrol. 2013;124(3-4):11-8.	OPN plays a significant role in the progression of glomerulosclerosis	increased expression		mouse	co-expression with the CD44 ligand OPN suggesteed interactions between these molecules	podocyte injury	kidney, glomerulosclerosis
Sano N, Kitazawa K, Sugisaki T.	Localization and roles of CD44, hyaluronic acid and osteopontin in IgA nephropathy.	Nephron. 2001 Dec;89(4):416-21.	expressed in tubular cells and interstitial infiltrating cells	expressed in mesangial cells, crescents, tubular cells and interstitial infiltrating cells		homo sapiens	correlation between glomerular/interstitial hyaluronate deposition and CD44 expression; correlation of interstitial CD44 and OPN; interstitium CD44 and OPN correlated with tubulointerstitial damage	co-expression in areas of tubulointerstitial injury	kidney, IgA nephropathy
Wang JT, Jiao P, Zhou Y, Liu Q.	Protective Effect of Dihydromyricetin Against Lipopolysaccharide-Induced Acute Kidney Injury in a Rat Model.	Med Sci Monit. 2016 Feb 11;22:454-9.	decrease in the expression levels	decrease in the expression of CD44 levels		rat		acute injury response	kidney, injury
Sibalic V, Fan X, Loffing J, Wüthrich RP.	Upregulated renal tubular CD44, hyaluronan, and osteopontin in kdkd mice with interstitial nephritis.	Nephrol Dial Transplant. 1997 Jul;12(7):1344-53.	renal expression correlates with disease severity	following injury, CD44s and variants are expressed by tubular epithelial cells, lymphocytes, macrophages	kidney	mouse		HA accumulates interstitially, particularly in cortical areas	kidney, tubular injury
Iguchi S, Nishi S, Ikegame M, Hoshi K, Yoshizawa T, Kawashima H, Arakawa M, Ozawa H, Gejyo F.	Expression of osteopontin in cisplatin-induced tubular injury.	Nephron Exp Nephrol. 2004;97(3):e96-105.	acute injury phase: induced in damaged tubular lumens; late recovery phase: present in dilated and flattened tubular epithelial cells with regenerative appearance	immunostainng matches OPN	renal tubule cells	mouse	PCNA staining co-localized with OPN and CD44	tissue repair	kidney, cisplatin-induced tubular injury
Lewington AJ, Padanilam BJ, Martin DR, Hammerman MR.	Expression of CD44 in kidney after acute ischemic injury in rats.	Am J Physiol Regul Integr Comp Physiol. 2000 Jan;278(1):R247-54.	OPN, not normally expressed in the renal proximal tubule, induced in regenerating tubules 3 days after acute ischemic injury	no expression in nonischemic kidneys, several mRNAs for CD44 within 1 day after injury	renal tubules	rat		tissue repair	kidney, ischemic injury
Wang L, Chen W, Peng Z, Liu C, Zhang C, Guo Z.	Vorinostat protects against calcium oxalate-induced kidney injury in mice.	Mol Med Rep. 2015 Sep;12(3):4291-4297.	expression levels in the HDAC inhibitor-treated group were lower	expression levels of CD44 in the HDAC inhibitor-treated group were lower		mouse		stone formation	kidney, injury
Vervaet BA, D'Haese PC, De Broe ME, Verhulst A.	Crystalluric and tubular epithelial parameters during the onset of intratubular nephrocalcinosis: illustration of the 'fixed particle' theory in vivo.	Nephrol Dial Transplant. 2009 Dec;24(12):3659-68.	increase with crystal retention	increase with crystal retention		rat	an altered epithelial phenotype with crystal-binding properties precedes crystal adhesion	crystal adhesion	kidney, intratubular nephrocalcinosis
Khan SR, Glenton PA, Byer KJ.	Modeling of hyperoxaluric calcium oxalate nephrolithiasis: experimental induction of hyperoxaluria by hydroxy-L-proline.	Kidney Int. 2006 Sep;70(5):914-23.	intense in cells lining the tubules that contained crystals	intense in cells lining the tubules that contained crystals		rat		nephrolithiasis	kidney, stones
Verhulst A, Asselman M, Persy VP, Schepers MS, Helbert MF, Verkoelen CF, De Broe ME.	Crystal retention capacity of cells in the human nephron: involvement of CD44 and its ligands hyaluronic acid and osteopontin in the transition of a crystal binding- into a nonadherent epithelium.	J Am Soc Nephrol. 2003 Jan;14(1):107-15.	expressed at the apical membrane of proliferating tubular cells, lost in confluent cells	expressed at the apical membrane of proliferating tubular cells, basolateral in confluent cells	tubular/collecting duct cells	homo sapiens		crystal retention may depend on the expression of CD44-, OPN-, and-HA rich cell coats by damaged distal tubular epithelium	kidney, stones
Peng Z, Chen W, Wang L, Ye Z, Gao S, Sun X, Guo Z.	Inhalation of hydrogen gas ameliorates glyoxylate-induced calcium oxalate deposition and renal oxidative stress in mice.	Int J Clin Exp Pathol. 2015 Mar 1;8(3):2680-9.	renal expression of OPN markedly increased in glyoxylate-treated mice	renal expression of CD44 markedly increased in glyoxylate-treated mice		mouse	H2 significantly attenuated the increase of OPN, CD44		kidney, stones
Okada A, Yasui T, Fujii Y, Niimi K, Hamamoto S, Hirose M, Kojima Y, Itoh Y, Tozawa K, Hayashi Y, Kohri K.	Renal macrophage migration and crystal phagocytosis via inflammatory-related gene expression during kidney stone formation and elimination in mice: Detection by association analysis of stone-related gene expression and microstructural observation.	J Bone Miner Res. 2010 Dec;25(12):2701-11.	expression on the apical side of tubular cells, seemed to be contained in crystal depositions	CD44 and MHC class II were upregulated around crystal-formation sites		mouse		crystal phagocytosis	kidney, stones
Taguchi K, Okada A, Kitamura H, Yasui T, Naiki T, Hamamoto S, Ando R, Mizuno K, Kawai N, Tozawa K, Asano K, Tanaka M, Miyoshi I, Kohri K.	Colony-stimulating factor-1 signaling suppresses renal crystal formation.	J Am Soc Nephrol. 2014 Aug;25(8):1680-97.	increased expression over 6 days	increased CD44 expression over 6 days		mouse	migrating macrophages eliminate renal crystals in hyperoxaluria	crystal formation	kidney, stones
Taguchi K, Okada A, Hamamoto S, Unno R, Moritoki Y, Ando R, Mizuno K, Tozawa K, Kohri K, Yasui T.	M1/M2-macrophage phenotypes regulate renal calcium oxalate crystal development.	Sci Rep. 2016 Oct 12;6:35167.	expression reduced by M2 macrophage treatment	CD44 expression reduced by M2 macrophage treatment	renal tubular cells	mouse	M2 macrophages reduced expression of crystal-related genes, M1 increased expression of pro-inflammatory genes	crystal phagocytic rate	kidney, stones
Asselman M, Verhulst A, De Broe ME, Verkoelen CF.	Calcium oxalate crystal adherence to hyaluronan-, osteopontin-, and CD44-expressing injured/regenerating tubular epithelial cells in rat kidneys.	J Am Soc Nephrol. 2003 Dec;14(12):3155-66.	after 4-8 d expressed at the luminal membranes of injured/regenerating tubular epithelial cells	after 4-8 d expressed at the luminal membranes of injured/regenerating tubular epithelial cells	tubular epithelial cells	rat		hyaluronan, OPN, and CD44 play important roles in calcium oxalate crystal binding	kidney, stones

Qi S, Wang Q, Xie B, Chen Y, Zhang Z, Xu Y.	P38 MAPK signaling pathway mediates COM crystal-induced crystal adhesion change in rat renal tubular epithelial cells.	Urolithiasis. 2019 Jun 10.	induced by crystals	induced by crystals	renal tubular epithelial cells	rat		calcium oxalate monohydrate (COM) crystals	kidney, stones
Chen W, Liu WR, Hou JB, Ding JR, Peng ZJ, Gao SY, Dong X, Ma JH, Lin QS, Lu JR, Guo ZY.	Metabolomic analysis reveals a protective effect of Fu-Fang-Jin-Qian-Chao herbal granules on oxalate-induced kidney injury.	Biosci Rep. 2019 Feb 22;39(2). pii: BSR20181833.	decreased expression under treatment	decreased expression under treatment		mouse		kidney injury, treatment with Fu-Fang-Jin-Qian-Chao granules	kidney, stones
Li Y, Zhang J, Liu H, Yuan J, Yin Y, Wang T, Cheng B, Sun S, Guo Z.	Curcumin ameliorates glyoxylate-induced calcium oxalate deposition and renal injuries in mice.	Phytomedicine. 2019 Aug;61:152861.	high expression attenuated by curcumin	high expression attenuated by curcumin		mouse	antioxidant curcumin acts through the Nrf2 signaling pathway.	nephrolithiasis	kidney, stones
Martínez-Martínez E, Ibarrola J, Fernández-Celis A, Calvier L, Leroy C, Cachofeiro V, Rossignol P, López-Andrés N.	Galectin-3 pharmacological inhibition attenuates early renal damage in spontaneously hypertensive rats.	J Hypertens. 2018 Feb;36(2):368-376.	elevated	CD44 elevated		rat	elevation of the inflammatory mediators OPN and cd44 in spontaneous hypertension	renal damage	hypertension
Kang JA, Zhou Y, Weis TL, Liu H, Ulaszek J, Satgurunathan N, Zhou L, van Besien K, Crispino J, Verma A, Low PS, Wickrema A.	Osteopontin regulates actin cytoskeleton and contributes to cell proliferation in primary erythroblasts.	J Biol Chem. 2008 Mar 14;283(11):6997-7006.	highly expressed and secreted during differentiation	expressed	erythroblast	mouse, human		proliferation	hematopoiesis
Xiao HB, Lu XY, Sun ZL, Zhang HB.	Kaempferol regulates OPN-CD44 pathway to inhibit the atherogenesis of apolipoprotein E deficient mice.	Toxicol Appl Pharmacol. 2011 Dec 15;257(3):405-11.	plasma OPN, aortic OPN expression up in ApoE- mice, reduced by Kaempferol	aortic CD44 expression up in ApoE- mice, reduced by Kaempferol		mouse		atherogenesis	atherosclerosis
Anwar A, Li M, Frid MG, Kumar B, Gerasimovskaya EV, Riddle SR, McKeon BA, Thukaram R, Meyrick BO, Fini MA, Stenmark KR.	Osteopontin is an endogenous modulator of the constitutively activated phenotype of pulmonary adventitial fibroblasts in hypoxic pulmonary hypertension.	Am J Physiol Lung Cell Mol Physiol. 2012 Jul 1;303(1):L1-L11.	increased expression of OPN	increased expression of CD44	fibroblast	ox, human		vascular remodeling	lung, hypoxic pulmonary hypertension
Lee YH, Petkova AP, Granneman JG.	Identification of an adipogenic niche for adipose tissue remodeling and restoration.	Cell Metab. 2013 Sep 3;18(3):355-67.	high expression in M2 marcophages	CD44 expression in PDGFRα+ progenitors		mouse		preadipocyte proliferation	adipose tissue remodeling
Bertola A, Deveaux V, Bonnafous S, Rousseau D, Anty R, Wakkach A, Dahman M, Tordjman J, Clément K, McQuaid SE, Frayn KN, Huet PM, Gugenheim J, Lotersztajn S, Le Marchand-Brustel Y, Tran A, Gual P.	Elevated expression of osteopontin may be related to adipose tissue macrophage accumulation and liver steatosis in morbid obesity.	Diabetes. 2009 Jan;58(1):125-33.	fat loading in HepG2 promotes OPN expression; expression in adipose tissue was enhanced independently of insulin resistance and hepatic steatosis		HepG2, adipocytes	mouse, human	hepatic OPN and CD44 expressions were related to insulin resistance and steatosis		adiposity
De Koster J, Strieder-Barboza C, de Souza J, Lock AL, Contreras GA.	Effects of body fat mobilization on macrophage infiltration in adipose tissue of early lactation dairy cows.	J Dairy Sci. 2018 Aug;101(8):7608-7613.	local expression of OPN during macrophage infiltration	local expression of CD44 during macrophage infiltration		cow		fat mobilization	adiposity
Liu LF, Kodama K, Wei K, Tolentino LL, Choi O, Engleman EG, Butte AJ, McLaughlin T.	The receptor CD44 is associated with systemic insulin resistance and proinflammatory macrophages in human adipose tissue.	Diabetologia. 2015 Jul;58(7):1579-86.	elevated expression	serum concentrations and tissue gene expression of CD44 increased	adipose tissue macrophages	homo sapiens		localized inflammation	diabetes
Zhao X, Chen X, Zhang Y, George J, Cobbs A, Wang G, Li L, Emmett N.	Kidney Injury Molecule-1 Is Upregulated in Renal Lipotoxicity and Mediates Palmitate-Induced Tubular Cell Injury and Inflammatory Response.	Int J Mol Sci. 2019 Jul 11;20(14). pii: E3406.	induced by administration of saturated fatty acid palmitate	induced by administration of saturated fatty acid palmitate	primary renal tubular epithelial cells	rat	KIM-1 is an inducer		diabetic nephropathy
Judd LM, Andringa A, Rubio CA, Spicer Z, Shull GE, Miller ML.	Gastric achlorhydria in H/K-ATPase-deficient (Atp4a(-/-)) mice causes severe hyperplasia, mucocystic metaplasia and upregulation of growth factors.	J Gastroenterol Hepatol. 2005 Aug;20(8):1266-78.	upregulated	upregulated		mouse	upregulation of Reg IIIγ and δ, TFF3, OPN and CD44, reduction in Reg IIIβ and TFF1		gastric achlorhydria
Seth D, Duly A, Kuo PC, McCaughan GW, Haber PS.	Osteopontin is an important mediator of alcoholic liver disease via hepatic stellate cell activation.	World J Gastroenterol. 2014 Sep 28;20(36):13088-104.	OPN expression positively correlated with disease severity	CD44 expression increased in alcoholic liver disease, and in vivo and in vitro with alcohol administration.	hepatic stellate cell	mouse, human			liver, alcoholic disease
Schulien I, Hockenjos B, Schmitt-Graeff A, Perdekamp MG, Follo M, Thimme R, Hasselblatt P.	The transcription factor c-Jun/AP-1 promotes liver fibrosis during non-alcoholic steatohepatitis by regulating Osteopontin expression.	Cell Death Differ. 2019 Sep;26(9):1688-1699.	expressed	expressed	non-parenchymal liver cells	homo sapiens, mouse	regulated by c-JUN	fibrosis	fatty liver disease, non-alcoholic
Nowwarote N, Sukarawan W, Pavasant P, Foster BL, Osathanon T.	Basic fibroblast growth factor regulates phosphate/pyrophosphate regulatory genes in stem cells isolated from human exfoliated deciduous teeth.	Stem Cell Res Ther. 2018 Dec 10;9(1):345.	induced by mineralization	expressed mesenchymal marker	primary tooth cells	homo sapiens			tooth development
Salehinejad J, Saghafi S, Sharifi N, Zare-Mahmoodabadi R, Saghravanian N, Ghazi N, Shakeri MT.	Evaluation of osteopontin and CD44v6 expression in odontogenic cystic lesions by immunohistochemistry.	Pathol Res Pract. 2012 Jul 15;208(7):410-4.	OPN immunostaining in all lining epithelial cells	CD44v6 immunostaining in all lining epithelial cells		homo sapiens			odontogenic cycst
Wang YP, Liu BY.	High expression of osteopontin and CD44v6 in odontogenic keratocysts.	J Formos Med Assoc. 2009 Apr;108(4):286-92.	strong cytoplasmic immunostaining in lining epithelial cells	diffuse and strong membranous CD44v6 staining in nearly all lining epithelial cells		homo sapiens	binding of OPN to odontogenic keratocyst lining epithelial cells via CD44v6	migration, invasion, spread of lining epithelial cells into cancellous bone	odontogenic keratocycst
Zheng H, Li S.	Reduced miRNA‑214 expression in oral mucosa contributes to the pathogenesis of oral lichen planus by targeting CD44.	Mol Med Rep. 2018 Jan;17(1):1919-1925.	overexpression of OPN	overexpression of CD44	HeLa, Raj, Jurkat	homo sapiens	OPN suppresses the apoptosis of activated CD8+ T cells via CD44	T‑cell mediated autoimmunity and affects the skin, scalp, nails and mucosa	oral lichen planus
Santarelli A, Mascitti M, Rubini C, Bambini F, Zizzi A, Offidani A, Ganzetti G, Laino L, Cicciù M, Lo Muzio L.	Active inflammatory biomarkers in oral lichen planus.	Int J Immunopathol Pharmacol. 2015 Dec;28(4):562-8.	correlation between osteopontin expression and a high degree of inflammation			homo sapiens	expression of osteopontin and CD44 was higher in OLP than in controls	inflammation	oral lichen planus
Staibano S, Merolla F, Testa D, Iovine R, Mascolo M, Guarino V, Castellone MD, Di Benedetto M, Galli V, Motta S, Melillo RM, De Rosa G, Santoro M, Celetti A.	OPN/CD44v6 overexpression in laryngeal dysplasia and correlation with clinical outcome.	Br J Cancer. 2007 Dec 3;97(11):1545-51.	elevated in all severe dysplasias, but not in hyperplasias	CD44v6 overexpressin in dysplasia		homo sapiens	OPN expression was paralleled by cell surface reactivity for CD44v6	OPN correlated with degree of dysplasia and reduced disease-free survival; CD44v6 correlated negatively with disease-free survival	laryngeal dysplasia

Poole DH, Ndiaye K, Pate JL.	Expression and regulation of secreted phosphoprotein 1 in the bovine corpus luteum and effects on T lymphocyte chemotaxis.	Reproduction. 2013 Oct 21;146(6):527-37	expression in corpus luteum with maturation; upregulated by PGF2α or TNFα	expression of CD44 increased during the estrous cycle but did not change during luteal regression	luteal cells	ox		lymphocyte chemotaxis, luteal regression	pregnancy
Gnainsky Y, Granot I, Aldo P, Barash A, Or Y, Mor G, Dekel N.	Biopsy-induced inflammatory conditions improve endometrial receptivity: the mechanism of action.	Reproduction. 2015 Jan;149(1):75-85.	expression by dendritic cells upon stimulation	expression by dendritic cells upon stimulation	endometrial epithelial cells	homo sapiens	TNFα-induced endometrial stromal cell cytokine expression, attracting monocytes, inducing their DC differentiation	embryo attachment	pregnancy
Marei WFA, Wathes DC, Raheem KA, Mohey-Elsaeed O, Ghafari F, Fouladi-Nashta AA.	Influence of hyaluronan on endometrial receptivity and embryo attachment in sheep.	Reprod Fertil Dev. 2017 Sep;29(9):1763-1773.	HA infusion increased OPN expression	HA infusion increased CD44v6 expression		sheep		embryo attachment	pregnancy
Shin T, Ahn M, Kim H, Moon C, Kang TY, Lee JM, Sim KB, Hyun JW.	Temporal expression of osteopontin and CD44 in rat brains with experimental cryolesions.	Brain Res. 2005 Apr 11;1041(1):95-101.	increased at days 4 and 7 post-injury, then slightly declined; in activated microglia/macrophages, some neurons	abundant in the majority of macrophages and in reactive astrocytes, but not in neurons, 4-14 days		rat	OPN production during the early stage; may interact with CD44 in the damaged cerebral cortex	response to tissue damage	cryolesion
Shaik S, Wu X, Gimble J, Devireddy R.	Effects of Decade Long Freezing Storage on Adipose Derived Stem Cells Functionality.	Sci Rep. 2018 May 25;8(1):8162.	negative impact on osteogenic potential, decrease in OPN gene expression	expression above 95%	adipose-derived stem cells	homo sapiens		long-term cryopreservation	cryostorage
Pop DM, SoriŢău O, Şuşman S, Rus-Ciucă D, Groza IŞ, Ciortea R, Mihu D, Mihu CM.	Potential of placental-derived human mesenchymal stem cells for osteogenesis and neurogenesis.	Rom J Morphol Embryol. 2015;56(3):989-96.	OPN+	chorionic and amniotic mesenchymal stem cells are CD44+	placental-derived mesenchymal stem cells	homo sapiens		differentiation	differentiation, stem cell
Zohar R, Sodek J, McCulloch CA.	Characterization of stromal progenitor cells enriched by flow cytometry.	Blood. 1997 Nov 1;90(9):3471-81.	osteopontin expression	CD44 expression	stromal progenitor cells (fetal periosteum) after attachment	rat	suspensions of S cells do not express differentiation-associated markers (e.g. OPN)	attachment	differentiation
Ghilzon R, McCulloch CA, Zohar R.	Stromal mesenchymal progenitor cells.	Leuk Lymphoma. 1999 Jan;32(3-4):211-21.	expression after attachment	expression after attachment	stromal mesenchymal progenitor cells	rat		differentiation	differentiation
Zhang H, Miao Z, He Z, Yang Y, Wang Y, Feng M.	The existence of epithelial-to-mesenchymal cells with the ability to support hematopoiesis in human fetal liver.	Cell Biol Int. 2005 Mar;29(3):213-9.	expression	expression	fetal liver	human		epithelial-to-mesenchymal transition	development
Hu P, Pu Y, Li X, Zhu Z, Zhao Y, Guan W, Ma Y.	Isolation, in vitro culture and identification of a new type of mesenchymal stem cell derived from fetal bovine lung tissues.	Mol Med Rep. 2015 Sep;12(3):3331-3338.	positive for the expression of OPN following osteogenic induction	CD44 expression	fetal lung-derived mesenchymal stem cells	ox		repair and regeneration	tissue remodeling
Rouschop KM, Roelofs JJ, Sylva M, Rowshani AT, Ten Berge IJ, Weening JJ, Florquin S.	Renal expression of CD44 correlates with acute renal allograft rejection.	Kidney Int. 2006 Sep;70(6):1127-34.	plasma OPN upregulated	increased sCD44 plasma levels, correlation with tubular CD44 expression		homo sapiens	during acute rejection, upregulation of CD44, HA, and OPN in the graft	graft rejection	transplantation
Zohar R, Suzuki N, Suzuki K, Arora P, Glogauer M, McCulloch CA, Sodek J.	Intracellular osteopontin is an integral component of the CD44-ERM complex involved in cell migration.	J Cell Physiol. 2000 Jul;184(1):118-30.	intracellular OPN, no uptake of exogenous OPN	CD44(cytoplasmic tail)-ERM	fetal fibroblasts, periodontal ligament cells, macrophages, breast cancer cells	mouse, rat, human	increased OPN and CD44 expression in migrating cells	cell migration	

Table S1C

author	title	journal	OPN	CD44	cells	species	properties	effect	condition
Chellaiah MA, Kizer N, Biswas R, Alvarez U, Strauss-Schoenberger J, Rifas L, Rittling SR, Denhardt DT, Hruska KA.	Osteopontin deficiency produces osteoclast dysfunction due to reduced CD44 surface expression.	Mol Biol Cell. 2003 Jan;14(1):173-89.	expressed in basolateral, clear zone, and ruffled border membranes; deposited in the resorption pits	expression on the osteoclast surface stimulated by OPN; CD44 is required for osteoclast motility and bone resorption	osteoclast	mouse		OPN induces CD44 expression on the cell surface	bone remodelling
Chellaiah MA, Biswas RS, Rittling SR, Denhardt DT, Hruska KA.	Rho-dependent Rho kinase activation increases CD44 surface expression and bone resorption in osteoclasts.	J Biol Chem. 2003 Aug 1;278(31):29086-97.	55-60% decrease in basal level ROK-alpha phosphorylation in OPN-/- compared with wild type	CD44 expression was promoted by ROK-α, CD44 and ERM phosphorylation, CD44/ERM/actin complex	osteoclast	mouse	OPN/αvβ3 generated Rho signaling was required for CD44 expression; anti-αv, -β3, or -CD44 inhibited migration and bone resorption	migration and bone resorption	bone resorption
Khan SA, Cook AC, Kappil M, Günthert U, Chambers AF, Tuck AB, Denhardt DT.	Enhanced cell surface CD44 variant (v6, v9) expression by osteopontin in breast cancer epithelial cells facilitates tumor cell migration: novel post-transcriptional, post-translational regulation.	Clin Exp Metastasis. 2005;22(8):663-73.		OPN induces CD44v6, v9 expression; up-regulation of CD44s mRNA, but no change in CD44v6, v8, v9, v10; elevated CD44s, v6, v9 protein; at the level of total protein, increaase in CD44s, v6	21NT breast tumor	human	OPN-mediated cell migration blocked by anti-pan CD44 antibody, anti-CD44v6, anti-CD44v9	migration	cancer, breast
Todaro M, Gaggianesi M, Catalano V, Benfante A, Iovino F, Biffoni M, Apuzzo T, Sperduti I, Volpe S, Cocorullo G, Gulotta G, Dieli F, De Maria R, Stassi G.	CD44v6 is a marker of constitutive and reprogrammed cancer stem cells driving colon cancer metastasis.	Cell Stem Cell. 2014 Mar 6;14(3):342-56.			cancer stem cells		cytokines hepatocyte growth factor (HGF), osteopontin (OPN), and stromal-derived factor 1α (SDF-1), secreted from tumor associated cells, increase CD44v6 expression in CR-CSCs by activating the Wnt/β-catenin pathway		cancer, colon
Huang J, Pan C, Hu H, Zheng S, Ding L.	Osteopontin-enhanced hepatic metastasis of colorectal cancer cells.	PLoS One. 2012;7(10):e47901.	highly expressed in metastatic hepatic lesions from CRC compared to primary CRC tissue and adjacent normal mucosa	strongly expressed in hepatocytes from normal liver	liver metastasis		OPN knockdown decreases CD44 expression, OPN transection increases CD44 expression		cancer, colorectal
Chang PL, Hsieh YH, Wang CC, Juliana MM, Tsuruta Y, Timares L, Elmets C, Ho KJ.	Osteopontin facilitates ultraviolet B-induced squamous cell carcinoma development.	J Dermatol Sci. 2014 Aug;75(2):121-32.	OPN protected basal keratinocytes from undergoing apoptosis upon UVB exposure				the expression of CD44 and FAK in WT mice epidermis was greater than that of OPN-null mice		cancer, cutaneous squamous cell carcinoma
Kim MS, Park MJ, Moon EJ, Kim SJ, Lee CH, Yoo H, Shin SH, Song ES, Lee SH.	Hyaluronic acid induces osteopontin via the phosphatidylinositol 3-kinase/Akt pathway to enhance the motility of human glioma cells.	Cancer Res. 2005 Feb 1;65(3):686-91.	OPN ia a transcriptional target of HA		glioma	human	HA binds to CD44 and induces OPN expression; in PTEN deficient cells but not in wild-type cells	motility is induced	cancer, glioma
Marroquin CE, Downey L, Guo H, Kuo PC.	Osteopontin increases CD44 expression and cell adhesion in RAW 264.7 murine leukemia cells.	Immunol Lett. 2004 Aug 15;95(1):109-12.		OPN upregulates plasma membrane total CD44 via prolonging protein half-life; CD44v6 is not altered		mouse	blockade of alphavbeta3-integrin ablates the OPN-dependent increase in CD44		cancer, leukemia
Gao C, Guo H, Downey L, Marroquin C, Wei J, Kuo PC.	Osteopontin-dependent CD44v6 expression and cell adhesion in HepG2 cells.	Carcinogenesis. 2003 Dec;24(12):1871-8.		OPN up-regulated plasma membrane CD44v6 protein expression(but not CD44s) in a concentration- and time-dependent fashion	hepatocellular carcinoma cells	human	CD44v6 induction by OPN inhibitable with RGD peptide; OPN significantly increased CD44v6 protein synthesis, while simultaneously decreasing protein degradation. Steady-state mRNA levels of both CD44s and CD44v6 were unaltered in the presence of OPN stimulation	increased adhesion to HA	cancer, liver
Kwak TK, Sohn EJ, Kim S, Won G, Choi JU, Jeong K, Jeong M, Kwon OS, Kim SH.	Inhibitory effect of ethanol extract of Ocimum sanctum on osteopontin mediated metastasis of NCI-H460 non-small cell lung cancer cells.	BMC Complement Altern Med. 2014 Oct 27;14:419.	exogenous	expression induced by OPN	NCI-H460	human	OPN activated the expression of CD44	cell viability, adhesion, invasion	cancer, lung, small cell
Tajima K, Ohashi R, Sekido Y, Hida T, Nara T, Hashimoto M, Iwakami S, Minakata K, Yae T, Takahashi F, Saya H, Takahashi K.	Osteopontin-mediated enhanced hyaluronan binding induces multidrug resistance in mesothelioma cells.	Oncogene. 2010 Apr 1;29(13):1941-51.	OPN modulated CD44 isoform expression, which closely regulated HA binding	CD44v10 suppression increased multidrug resistance; enhanced p-Akt was diminished by CD44 siRNA	ACC-MESO-1	human	OPN is strongly involved in multidrug resistance by enhancing CD44 binding to HA	drug resistance	cancer, malignant pleural mesothelioma
Yang MC, Wang HC, Hou YC, Tung HL, Chiu TJ, Shan YS.	Blockade of autophagy reduces pancreatic cancer stem cell activity and potentiates the tumoricidal effect of gemcitabine.	Mol Cancer. 2015 Oct 12;14:179.	OPN stimulated LC3-II, ALDH1, CD44, and CD133 expression	CD44 expression	PANC-1	human		autophagy, cancer stem cell activity	cancer, pancreas
Samanna V, Wei H, Ego-Osuala D, Chellaiah MA.	Alpha-V-dependent outside-in signaling is required for the regulation of CD44 surface expression, MMP-2 secretion, and cell migration by osteopontin in human melanoma cells.	Exp Cell Res. 2006 Jul 15;312(12):2214-30.	exogenous OPN/transfected OPN	CD44 induction	melanoma cells	human	OPN signaling via integrin alphaV	OPN induces CD44 and MMP-2	cancer, melanoma
Desai B, Rogers MJ, Chellaiah MA.	Mechanisms of osteopontin and CD44 as metastatic principles in prostate cancer cells.	Mol Cancer. 2007 Mar 7;6:18.	siRNA, overexpression construct, mutant	increase in CD44 surface expression, interaction of CD44/MMP-9 on the cell surface	prostate cancer, PC3	human	RHO signaling; levels of MMP-9 activity reflect the CD44 surface expression pattern	migration	cancer, prostate
Desai B, Ma T, Zhu J, Chellaiah MA.	Characterization of the expression of variant and standard CD44 in prostate cancer cells: identification of the possible molecular mechanism of CD44/MMP9 complex formation on the cell surface.	J Cell Biochem. 2009 Sep 1;108(1):272-84.	stable transfection PC3-OPN	increase in the surface expression of v6, v10, sCD44 in PC3/OPN cells (no change in RNA or protein levels)	prostate cancer, PC3	human		surface expression, activation of MMP9	cancer, prostate
Robertson BW, Chellaiah MA.	Osteopontin induces beta-catenin signaling through activation of Akt in prostate cancer cells.	Exp Cell Res. 2010 Jan 1;316(1):1-11.	stable transfection PC3-OPN	OPN increases CD44 transcription through the nuclear import of β-catenin	PC3, DU145, LNCaP	human		CD44 expression, anti-apoptosis	cancer, prostate

Yang K, Tang Y, Habermehl GK, Iczkowski KA.	Stable alterations of CD44 isoform expression in prostate cancer cells decrease invasion and growth and alter ligand binding and chemosensitivity.	BMC Cancer. 2010 Jan 14;10:16.		enforced CD44s re-expression, knockdown of CD44v7-10 by RNAi	PC-3	human	Hyaluronan and OPN binding were strengthened by CD44s expression	growth, invasion	cancer, prostate
Erb U, Megaptche AP, Gu X, Büchler MW, Zöller M	CD44 standard and CD44v10 isoform expression on leukemia cells distinctly influences niche embedding of hematopoietic stem cells.	J Hematol Oncol. 2014 Mar 31;7:29.	EL4-v10, by catching OPN, migrated on bone marrow stroma	CD44v10	EL-4, EL-4-v10	mouse	EL4-v10 exosomes promoted OPN upregulation	CD44v10-stimulated OPN expression could drive hematopoietic stem cells out of the niche	cancer, thymoma
Abdulrahman N, Jaspard-Vinassa B, Fliegel L, Jabeen A, Riaz S, Gadeau AP, Mraiche F.	Na+/H+ exchanger isoform 1-induced osteopontin expression facilitates cardiac hypertrophy through p90 ribosomal S6 kinase.	Physiol Genomics. 2018 May 1;50(5):332-342.	upregulation of myocardial OPN	upregulation of CD44 regressed in OPN knockout mice		mouse	dilated hypertrophic phenotype expressed CD44 and p-RSK, effects regressed in the absence of OPN		cardiovascular, cardiac hypertrophy
Enkhjargal B, McBride DW, Manaenko A, Reis C, Sakai Y, Tang J, Zhang JH.	Intranasal administration of vitamin D attenuates blood-brain barrier disruption through endogenous upregulation of osteopontin and activation of CD44/P-gp glycosylation signaling after subarachnoid hemorrhage in rats.	J Cereb Blood Flow Metab. 2017 Jul;37(7):2555-2566.	neuroprotection by endogenous OPN; reversal by OPN siRNA	expression upregulated in disease, ameliorated by vitamin D3		rat	CD44 splice variant (55 kDa) upregulatred in diseease	blood-brain barrier disruption	CNS, subarachnoid hemorrhage
Marcondes MC, Ojakian R, Bortell N, Flynn C, Conti B, Fox HS.	Osteopontin expression in the brain triggers localized inflammation and cell death when immune cells are activated by pertussis toxin.	Mediators Inflamm. 2014;2014:358218.		CD44v6 induced by pertussis toxin		mouse	inflammatory infiltrate induced by OPN plus peripheral treatment with pertussis toxin, which activated peripheral cells to express CD44v6	cell death	CNS, inflammation
Nyström T, Dunér P, Hultgårdh-Nilsson A.	A constitutive endogenous osteopontin production is important for macrophage function and differentiation.	Exp Cell Res. 2007 Apr 1;313(6):1149-60.	OPN siRNA; cells with silenced OPN had a lower expression of IL-12 mRNA	reduced CD44 cell surface expression in siOPN cells; downregulation of CD44 mRNA expression in the siOPN cells	macrophage	mouse	impaired migration, increased rate of serum starvation-induced apoptosis in macrophages with silenced OPN	migration, apoptosis	immune system
Yuen L, Lam V, George J, Douglas MW, Ahlenstiel G.	KLRG1+ natural killer cells exert a novel antifibrotic function in chronic hepatitis B.	J Hepatol. 2019 Aug;71(2):252-264.		increased expression by osteopontin stimulation	terminally differentiated NK cells	human		hepatic stellate cell-derived osteopontin may cause KLRG1+ NK cell activation	hepatitis B
Yu XQ, Nikolic-Paterson DJ, Mu W, Giachelli CM, Atkins RC, Johnson RJ, Lan HY.	A functional role for osteopontin in experimental crescentic glomerulonephritis in the rat.	Proc Assoc Am Physicians. 1998 Jan-Feb;110(1):50-64.	anti-OPN inhibits upregulation of OPN	anti-OPN inhibits upregulation of CD44	kidney cells, immune cells	rat	anti-OPN treatment reduced glomerular injury and prevented a loss of renal function, reduced renal macrophage and T-cell accumulation	inflammation	kidney, crescentic glomerulonephritis
Choi SH, Kim AR, Nam JK, Kim JM, Kim JY, Seo HR, Lee HJ, Cho J, Lee YJ.	Tumour-vasculature development via endothelial-to-mesenchymal transition after radiotherapy controls CD44v6+ cancer cell and macrophage polarization.	Nat Commun. 2018 Nov 30;9(1):5108.			endothelial cells, KP lung tumor	mouse		Osteopontin stimulates proliferation in dormant CD44v6+ cells	radiotherapy
Kang HS, Liao G, DeGraff LM, Gerrish K, Bortner CD, Garantziotis S, Jetten AM.	CD44 plays a critical role in regulating diet-induced adipose inflammation, hepatic steatosis, and insulin resistance.	PLoS One. 2013;8(3):e58417.	expression greatly diminished in CD44KO-high fat diet liver	CD44-deficiency reduced susceptibility to high fat-diet-induced hepatic steatosis		mouse	inflammation induces OPN	inflammation	metabolic syndrome
Wang W, Li P, Li W, Jiang J, Cui Y, Li S, Wang Z.	Osteopontin activates mesenchymal stem cells to repair skin wound.	PLoS One. 2017 Sep 28;12(9):e0185346.	OPN stimulated mesenchymal stem cell migration	CD44 expression was decreased in the lesions of OPN knockout mice	mesenchymal stem cells	mouse		cell migration	wound healing
